# Cyclopentannulated Dihydrotetraazapentacenes

**DOI:** 10.1002/chem.202104203

**Published:** 2022-02-02

**Authors:** Robin Heckershoff, Steffen Maier, Thomas Wurm, Philipp Biegger, Kerstin Brödner, Petra Krämer, Marvin T. Hoffmann, Lukas Eberle, Jana Stein, Frank Rominger, Matthias Rudolph, Jan Freudenberg, Andreas Dreuw, A. Stephen K. Hashmi, Uwe H. F. Bunz

**Affiliations:** ^1^ Organisch-Chemisches Institut (OCI) Ruprecht-Karls-Universität Heidelberg Im Neuenheimer Feld 270 69120 Heidelberg Germany; ^2^ Interdisziplinäres Zentrum für Wissenschaftliches Rechnen (IWR) Ruprecht-Karls-Universität Heidelberg Im Neuenheimer Feld 205 A 69120 Heidelberg Germany; ^3^ Chemistry Department, Faculty of Science King Abdulaziz University Jeddah 21589 Saudi Arabia; ^4^ Centre for Advanced Materials (CAM) Im Neuenheimer Feld 225 69120 Heidelberg Germany

**Keywords:** aromaticity, azaarenes, catalysis, cyclopentannulation, cycloisomerization, silyl shift

## Abstract

The transition‐metal‐catalyzed cyclization of bissilylethynylated *N*,*N*’‐dihydrotetraazapentacene (TIPS‐TAP‐H_2_) into bissilylated cyclopenta[*fg*,*qr*]pentacenes is reported. Depending on the catalyst either none, one or two silyl groups migrate and change their positions in the formed five‐membered rings. The optoelectronic properties are quite similar, whereas the packing motifs differ dramatically. Control experiments and quantum chemical calculations were performed to investigate the mechanism of the reaction and the selectivity of the silyl shift.

## Graphical abstract

**The alkyne units** of bissilylethynylated *N*,*N’*‐dihydrotetraazapentacene are transferred into a dihydrotetraazapentacene core by a transition‐metal‐catalyzed cycloisomerization reaction to form a new type of cyclopentannulated tetraazapentacene. Depending on the applied catalyst systems, either silyl group retention or migration takes place, which gives rise to constitutional isomers that show comparable optoelectronic properties but completely different packing motifs in the solid states.

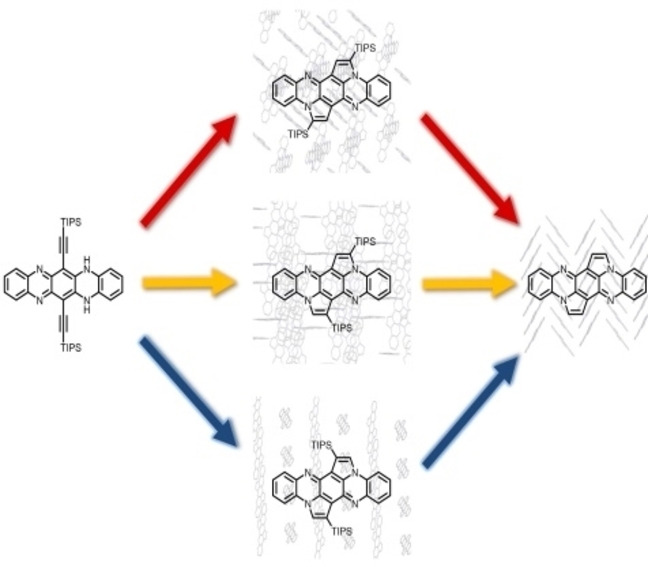

## Introduction

**Figure 1:**
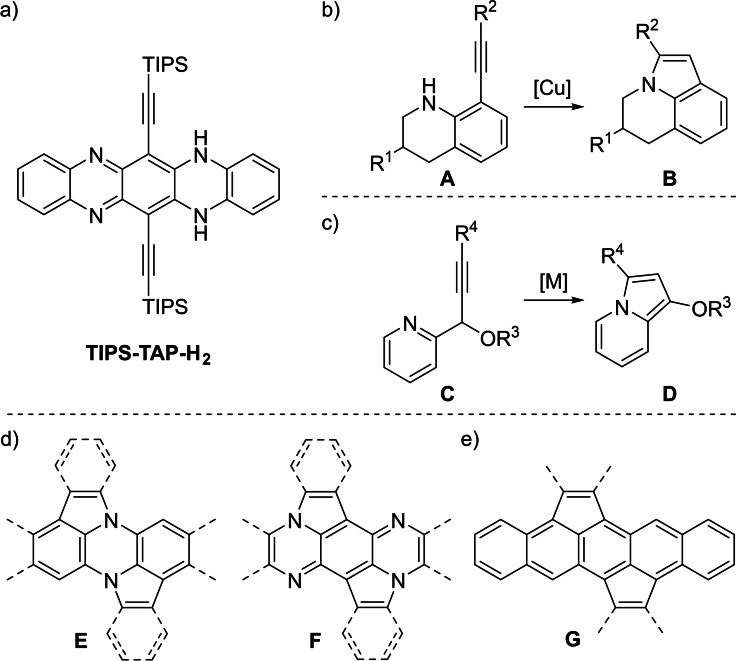
a) Structure of **TIPS‐TAP‐H_2_**; b) Cu‐catalyzed cycloisomerization of ethynyl‐substituted tetrahydroquinoline; c) transition metal catalyzed cycloisomerization of 2‐propargylpyridines; d) different modes of cyclopentannulation of dihydroazaacenes; e) cyclopentannulated pentacenes.

We herein present the transition‐metal‐catalyzed cyclization of a bissilylethynylated *N*,*N*’‐dihydrotetraazapentacene (TIPS‐TAP‐H_2_, Figure 1)^[1]^ into bissilylated cyclopenta[*fg*,*qr*]pentacenes. Cycloisomerization reactions of alkynes are atom economic tools to synthesize pyrrole‐, furan‐ or thiophene‐based heteroacenes.^[2]^ They also add five‐membered rings to already existing oligo‐ and polycycles. Attractive examples for the latter were reported by Pal et al. where tetrahydroquinoline **A** transforms into **B** under copper catalysis,^[3]^ as well some more examples involving dihydroquinolinones using Cu‐ and Pd‐based catalysts.^[4]^ While Gevorgyan et al. managed to synthesize fused pyrroles **D** by transition metal catalyzed transformation of 2‐propargyl pyridines **C**,^[5]^ these two pyrrole‐forming types of cycloisomerization are virtually unknown for larger annulated ring systems. Systems of the type **E** and **F** are underexplored, despite some early reports on dicarbazolylene (bisbenzannulated **E**) by Tomlinson et al. in the 1950 s^[6]^ and a few examples with the substructure of **E** in more recent patents.^[7]^ In the hydrocarbon analogues, cyclopentannulation stabilized acene‐types dicyclopenta[*fg,qr*]pentacenes **G** have been prepared by Chi et al.,^[8]^ Mastalerz et al.,^[9]^ Xiao et al.^[10]^ and others,^[11]^ yet not by cycloisomerizations. However, structures such as **E** and **F** are of interest as potential organic semiconductors, particularly if they are soluble/sublimable and processible; and their dicationic species could serve as models for the higher, inaccessible acenes.^[12]^

## Results and Discussion

**Scheme 1:**
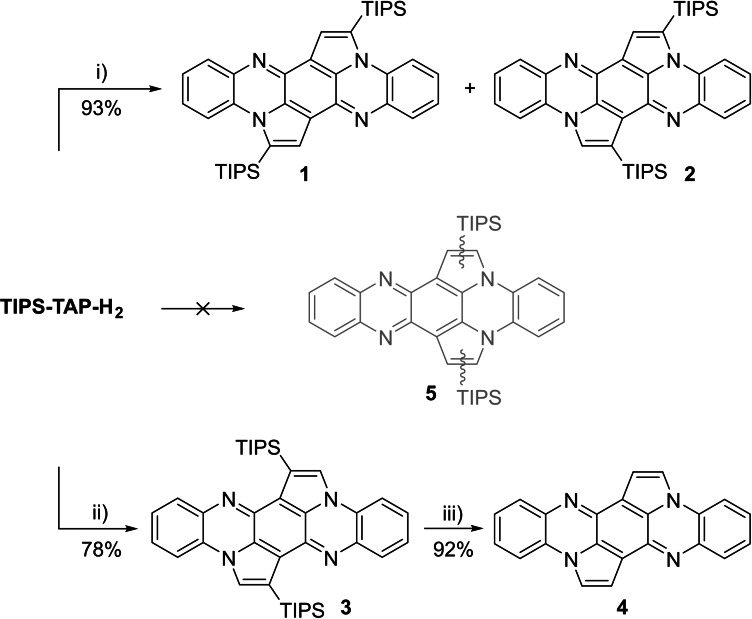
Synthetic route towards cyclopentannulated tetraazapentacenes **1**–**4**. Conditions: i) IPrAuNTf_2_ (10 mol%), DCE, 80 °C, 16 h; ii) PdCl_2_ (10 mol%), MeCN/CHCl_3_ 1 : 1, 80 °C, 18 h; iii) TBAF (10 equiv.), THF, 60 °C, 5 h.

When TIPS‐TAP‐H_2_ is treated with IPrAuNTf_2_ (10 mol%) in 1,2‐dichloroethane, not the expected product **5**, where both five‐membered rings are located on the same side of the molecule, is observed (Scheme 1). Instead three cycloisomerized species, where the five‐membered rings are located on opposite sides, are formed: **1** is the expected bispentannulated species, but in **2** one of the triisopropylsilyl (TIPS) groups migrated on the olefinic bond. In **3** both silyl groups underwent 1,2‐shifts. Silyl shifts in gold‐catalyzed cycloisomerization reactions were already observed for other silylethynyl annulations.^[13]^ When PdCl_2_ is used as catalyst in acetonitrile/chloroform, only isomer **3** is formed (78 %). Products in which both cyclopentannulations are observed at the same ring; i. e. **5**, are not observed under these conditions. Table 1 shows the results of our screening experiments (see Supporting Information chapter 1.3 for details). All Au(I) catalysts (entry 1–6) give predominantly **1** or **2**, but Au(III) catalysts (entry 7, 8) furnish **3**. Cu(acac)_2_ (entry 13) results in **1**, while PdCl_2_ in acetonitrile (entry 19) forms the doubly rearranged product **3**. Note that only our initially tested catalytic systems using IPrAuNTf_2_ (and some other Au(I) catalysts) or PdCl_2_ avoid side product formation and fully convert the starting material within one day. The raw yields in these cases are almost quantitative but in case of **3** some of the material is lost during workup due to the low solubility (∼0.7 mg/mL in DCM). **3** is non‐polar and elutes with petrol ether, while **1** and **2** are considerably more polar with higher solubility (∼45 mg/mL in DCM) and necessitate separation by preparative HPLC. These differences in solubility and polarity of the three isomers are likely caused by the different shielding of the nitrogens by the TIPS‐groups. Tetrabutylammonium fluoride in THF cleaves the silyl groups of **3** (analogous reactivity with **1** and **2**) to give the fully desilylated but insoluble (<12.0 μg/mL in DCM) species **4**. These compounds might be applicable for the use as organic semiconductors, **1**–**3** preferable for solution processing and **4** for vapor deposition methods.

**Table 1:** Catalyst screening for the conversion of **TIPS‐TAP‐H_2_**.^[a]^

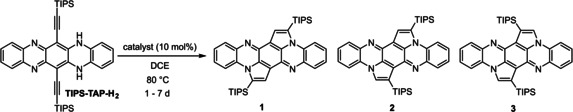						
Entry	Catalyst	t^[b]^	Comment^[c]^	Ratio of products^[d]^		
				1	2	3
1	**IPrAuNTf_2_**	1 d	–	**74**	**24**	**2**
2	XPhosAuCl/AgNTf_2_	5 d	–	**76**	22	2
3	IPr*AuCl/AgNTf_2_	5 d	–	**77**	22	1
4	Tris‐(2,4‐di‐tert.‐butylphenyl)‐phosphit‐goldchlorid/AgNTf_2_	1 d	–	50	45	5
5	PPh_3_AuCl/AgNTf_2_	1 d	–	13	**83**	4
6	Me_3_PAuCl/AgNTf_2_	1 d	–	51	44	5
7^[e]^	AuCl_3_ ⋅ 3H_2_O	3 d	S	0	0	**100**
8	PicAuCl_2_	1 d	S	0	0	**100**
9^[f]^	AgNTf_2_	7 d	E/S	86	14	traces
10	Pd(PPh_3_)_2_Cl_2_	3 d	S	20	59	21
11	Ni(PPh_3_)_2_Br_2_	3 d	E/S	0	0	traces
12	Cu(PPh_3_)_3_Br	7 d	E/S	traces	traces	traces
13	Cu(acac)_2_	7 d	E	99	1	0
14	ZnCl_2_‐TMEDA	7 d	E/S	traces	traces	traces
15	Pt(PPh_3_)_2_Cl_2_	7 d	E/S	0	traces	100
16	Rh(PPh_3_)_3_Cl	7 d	E/S	44	8	48
17	[Ir(cod)(PCy_3_)(py)]PF_6_	7 d	E/S	0	0	100
18	[Ru(Cp*)(CH_3_CN)_3_]PF_6_	7 d	E/S	0	0	traces
19^[e]^	**PdCl_2_**	1 d	–	0	0	**100**
20	*p*TsOH	7 d	E	0	0	0
21	HNTf_2_	7 d	E/S	0	0	0
22	–	7 d	E	0	0	0

[a] The reactions were performed using TIPS‐TAP‐H_2_ (15.5 μmol) and 10 mol% catalyst in 2.0 mL DCE, if not stated otherwise. [b] The reactions were run until TLC showed full conversion or for a maximum of 7 d. [c] E: a significant amount of educt was detected; S: a significant amount of side products was detected. [d] Determined by a combination of HPLC and ^1^H NMR analytics. [e] The reaction was performed in MeCN at 70 °C. [f] After 3 d very little conversion was detected and an additional 40 mol% AgNTf_2_ was added.

**Figure 2:**
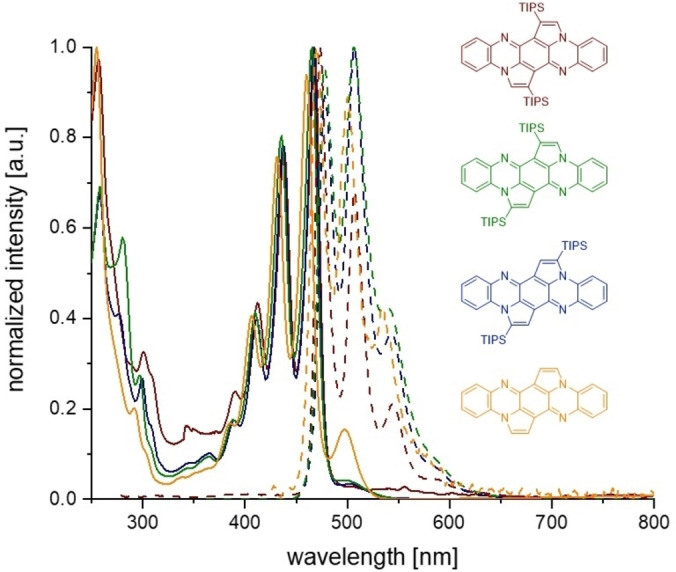
Normalized absorption spectra (full lines) and emission spectra (dotted lines) of **1**–**4** in DCM.

**1**–**3** are yellow (**4**: orange‐red) crystalline solids. They display strong yellow fluorescence in solution (quantum yields: 58–67 %) quenched upon aggregation. Figure 2 displays the normalized UV‐Vis spectra and the emission spectra of **1**–**4** in DCM solution. In the absorption spectra, the signals around 500 nm are probably due to an aggregation of the molecules in solution and disappear by addition of methanol to the DCM solutions (see the Supporting Information, Figure S11). We assume the formation of H‐aggregates which is supported by a red shift and decreasing intensities in the emission spectra. Absorbances are practically superimposable (**1**–**4**: *λ*_max, abs_=460–466 nm; *λ*_max, em_=470–478 nm). They are blue shifted by 93 nm compared to TIPS‐TAP‐H_2_ and differ only slightly in intensities of the vibronic fine‐structure of the emission spectra. The positions of the TIPS‐group(s) are negligible for the (opto)electronic properties of pentannulated **1**–**3**, which also is underlined by comparison to desilylated **4** (Table 2). As expected, Stokes shifts of the rigid compounds are small (318–539 cm^−1^). Compared to Miao's quinoid dihydrodimethyltetraazapentacene,^[14]^ absorption is significantly blue‐shifted by 85 nm as a consequence of pentannulation. At the molecular orbital level, this trend is also seen in the calculated HOMO‐LUMO gaps (Gaussian 16,^[15]^ DFT, B3LYP def2‐TZVP), which range between 3.02 and 3.07 eV for **3**–**1**, and are enlarged by ∼0.6 eV compared to the dimethylated compound.

**Table 2:** Experimental and calculated (gas‐phase) properties of **TIPS‐TAP‐H_2_**, **TIPS‐TAP** and the cyclized isomers **1**–**4**; calculated properties of the dication **3^2+^**.

Compound	*λ*_max, abs_ [nm]	*λ*_onset, abs_ [nm]	*λ*_max, em_ [nm]	Stokes shift [cm^−1^]	Quantum yield [%]	HOMO [eV]^[a]^	LUMO [eV]^[a]^	Gap Meas.^[b]^/calcd.^[a]^ [eV]
**TIPS‐TAP‐H_2_**	559	586	583		–	−5.24	−2.44	2.22/2.80
**TIPS‐TAP**	706	740	–	–	–	−5.60	−3.75	1.76/1.85
**1**	466	480	478/507/541	539	59	−5.41	−2.34	2.66/3.07
**2**	466	480	478/507/543	539	58	−5.44	−2.40	2.66/3.04
**3**	466	480	473/507/545	318	67	−5.48	−2.45	2.66/3.02
**4**	460	480	470/500/535	463	63	−5.49	−2.40	2.70/3.09
**3^2+^**	–	–	–	–	–	−12.7	−11.0	−/1.72

[a] Obtained from DFT calculations (Gaussian16,^[15]^ B3LYP/def2‐TZVP). [b] Obtained from UV‐Vis spectra using *λ*_max, abs_.

**Figure 3:**
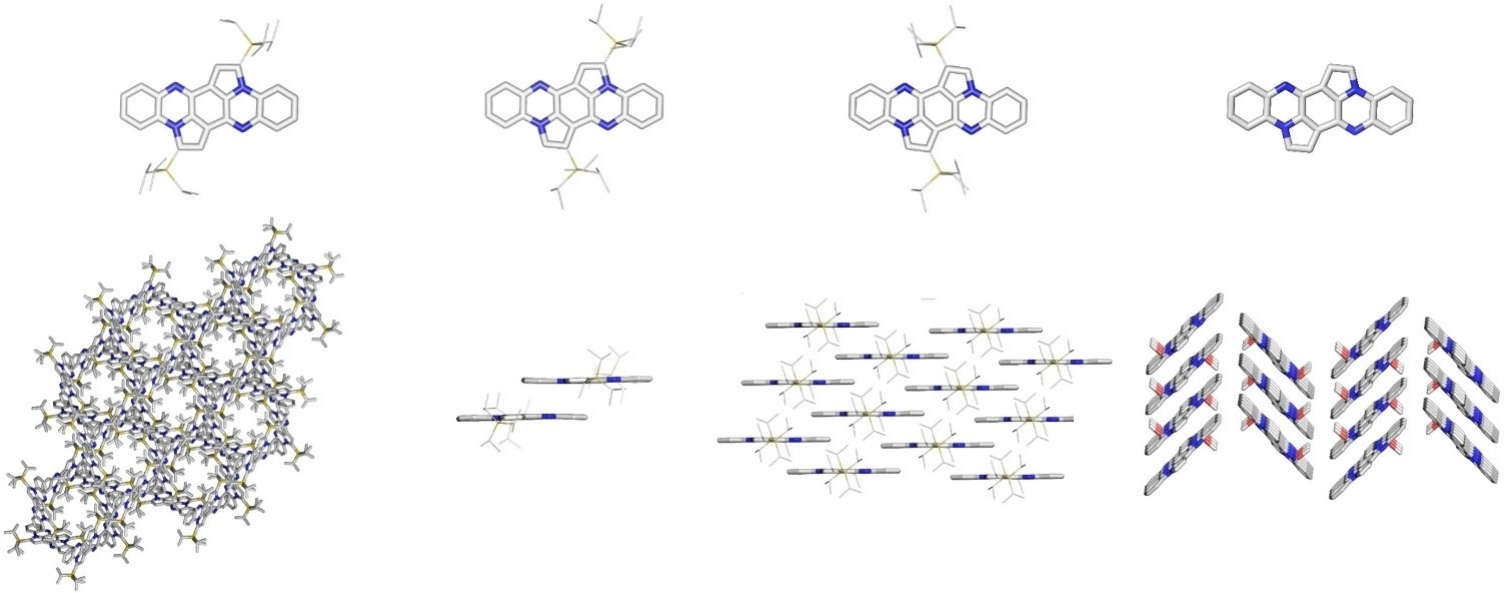
X‐ray structures (top) and packing motifs (bottom) from left to right: **1**, **2**, **3** and **4**. Silylethynyl substituents were reduced in size and solvent molecules omitted for clarity (except for **4**).

We obtained specimen suitable for single crystal analysis by vapor diffusion of methanol in a DCM solution (Figure 3). The molecular structures are in agreement with calculated bond lengths and bond angles at DFT level of theory (see the Supporting Information, Figure S15) – the position of the silyl groups significantly influences packing. **1** crystallizes with one independent molecule per unit cell. Each molecule has 8 closest neighbors in the 3D, honeycomb‐like structure, 4 of which are close to orthogonal and interact via CH ⋅⋅⋅ π short contacts involving the five‐membered rings with the central dihydrotetraazapentacene, while the remaining four interact with it via H ⋅⋅⋅ H contacts of the triisopropyl substituents (see Figure 3). π‐π stacking is not observed, the three channels per unit cell are filled with DCM. Mono‐silyl‐shifted **2** (one independent molecule per unit cell) crystallizes as isolated dimer pairs exhibiting π‐π‐stacking with a distance of 3.48 Å between the molecular backbones. The dimer pairs interact with the surrounding dimers via CH ⋅⋅⋅ π or H ⋅⋅⋅ H short contacts. Doubly shifted **3** displays a sandwich structure: The two independent molecules per unit cell are each part of a brickwall motif, which are rotated with respect to each other and separated by the triisopropylsilyl substituents. π‐stacking distances within the one dimensional declined stack amount to 3.58 Å to 4.11 Å. Inspecting the series **1**–**3**, for the desilylated compound **4** a herringbone motif was found with π‐π distances of 3.34 Å.

The tendency to form π‐stacks is increased the more square‐shaped the molecules are, similar what is observed when comparing acenes to their silylethynylated analogs.^[16]^

**Figure 4:**
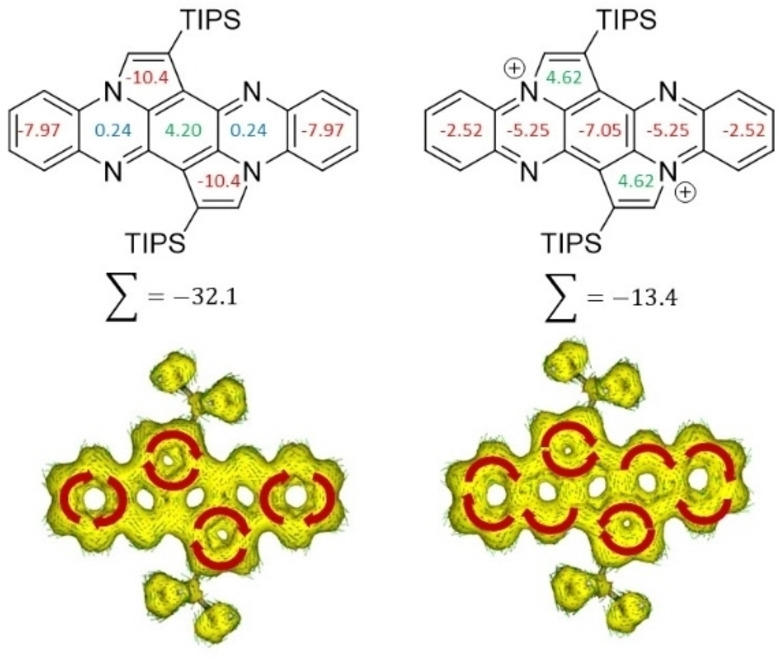
Top: Calculated NICS(1) values of **3** (left) and **3^2+^** (right). (Gaussian 16, geometry optimization: B3LYP/def2‐SVP/, B3LYP/def2‐TZVP; NICS calculations: GIAO‐method B3LYP/def2‐TZVP Bottom: AICD plot of **3** (left) and **3^2+^** (right). (TMS groups were used instead of TIPS; the red arrows indicate the ring current flow magnetic field, isovalue=0.02; magnetic field vector is orientated out of plane; AICD: CSGT‐method B3LYP/def2‐TZVP IOP(10/93=1)).

Figure 4 (top) displays the calculated nuclear‐independent chemical shifts (NICS(1))^[17]^ values of **3**. The outer benzene rings (−7.97 ppm) and the two five‐membered rings (−10.4 ppm) are aromatic, while the two hydropyrazine rings are non‐aromatic (0.24 ppm); the quinoid inner ring displays considerably reduced aromaticity (4.20 ppm). The anisotropy of the induced current density (AICD)^[18]^ plot of **3** also corroborates the results of the NICS calculations, exhibiting four diatropic (clockwise, highlighted in Figure 4, bottom) ring currents. Attempts to generate the dication of **3** using NOPF_6_, MnO_2_, PbO_2_, AgSbF_6_ fails even though a color change from yellow to red is observed upon addition of NOPF_6_ or AgSbF_6_. This coloration is reversible upon addition of water and is due to protonation or silver salt formation of **3**. Addition of HCl or TFA to solutions of **3** resulted in the same reversible color change (see the Supporting Information, Figure S12 for UV‐Vis spectra), proving that protonation is observed. Calculations underline that upon twofold oxidation not only one Clar sextet would be destroyed due to generation of the tetraazapentacene dicationic backbone, but also the five‐membered rings would gain antiaromatic character (similar to the cyclopentadienyl cation)^[19]^ explaining the difficulty in assessing this species.

**Scheme 2:**
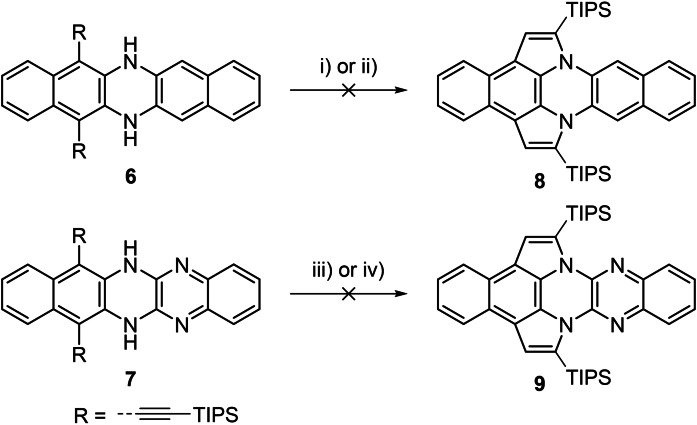
Control experiments to prove the suggested mechanism. Conditions: i) IPrAuNTf_2_ (10 mol%), DCE, 80 °C, 16 h; ii) PdCl_2_ (10 mol%), MeCN/CHCl_3_ 1 : 1, 80 °C, 18 h; iii) IPrAuNTf_2_ (10 mol%), DCE, 80 °C, 21 d; iv) PdCl_2_ (10 mol%), MeCN/CHCl_3_ 1 : 1, 80 °C, 21 d.

What is the mechanism of this 5‐endo‐dig cyclization? Calculations (see the Supporting Information, chapter 5) show that the hypothetical products **8** and **9** are thermodynamically more stable than their corresponding starting materials **6** and **7**. We treated **6** and **7** under optimized cycloisomerization conditions (Scheme 2), whereby **6** only gives the oxidized species^[20]^ and no cyclized product **8**, while **7** forms the singly cycloisomerized product (**10**, see the Supporting Information, chapter 1.2) after 3 weeks in 34 % yield, which decomposes upon longer reaction. The doubly cycloisomerized product **9** is not formed. These experiments suggest that the reaction initially takes place at the pyrazine and not at the dihydropyrazine ring.

**Scheme 3:**
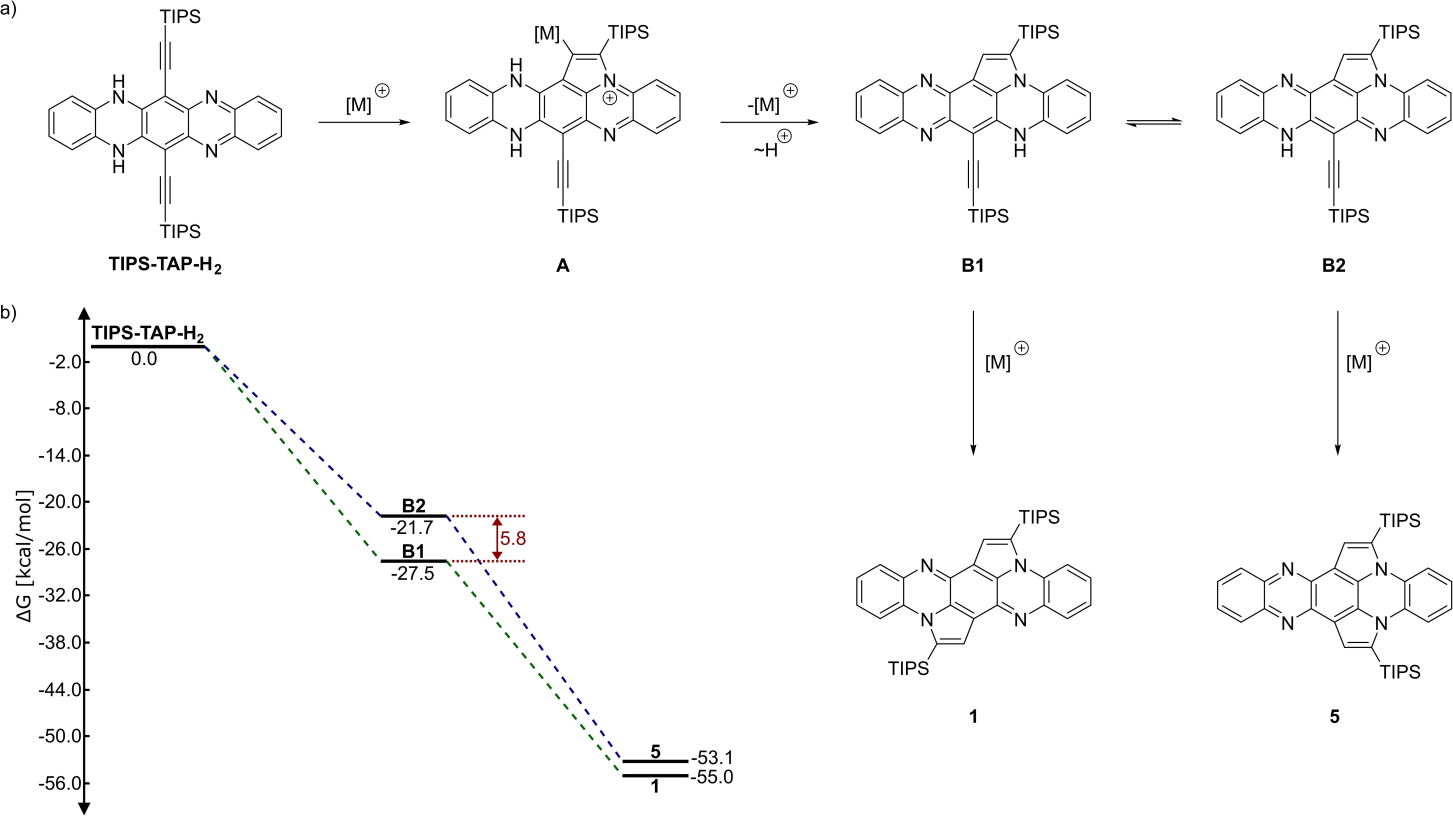
a) Proposed mechanism to explain the observed regioisomer **1**; b) reaction profile with calculated Gibbs energies (Q‐Chem 5.2.2,^[21]^ wB97X‐D3/pc‐1).

A rudimentary mechanism is depicted in Scheme 3. After activation of the alkyne triple bond via a transition metal, nucleophilic attack of the pyrazine nitrogen furnishes a cationic cyclopent‐annulated intermediate **A**. The preferred cyclization mode through the pyrazine‐substructure can be rationalized by an in plane orbital interaction between the lone pairs located on the nitrogen and the antibonding orbital of the alkyne, which seems to be favored over the out of plane interaction of the dihydropyrazine nitrogen. This reaction pathway is also supported by quantum chemical calculations, which have been conducted on a smaller model system with IPrAuNTf_2_ as catalyst. In addition, an alternative route is found through a gold vinylidene species which is not favorable (see Supporting Information, Figure S13/S14 for more details). After protodemetallation, an intermediate **B** is formed, which can exist as two tautomers, **B1** and **B2**. Calculations for the relative energies (Q‐Chem 5.2.2,^[21]^ wB97X‐D3/pc‐1) show **B1**, which leads to the observed product **1**, to be more stable (by 5.77 kcal/mol) than **B2**, which would lead to the regioisomeric compound **5**. Furthermore, the relative energies for **1** and **5** differ slightly (by 1.89 kcal/mol), which suggests a similar transition state for the reaction from **B1** to **1** and **B2** to **5**, respectively. Therefore a product distribution should be dominated by the ratio of **B1** to **B2** which heavily favors **B1** (Boltzmann population ratio at 353 K: ∼4000 : 1).

**Scheme 4:**
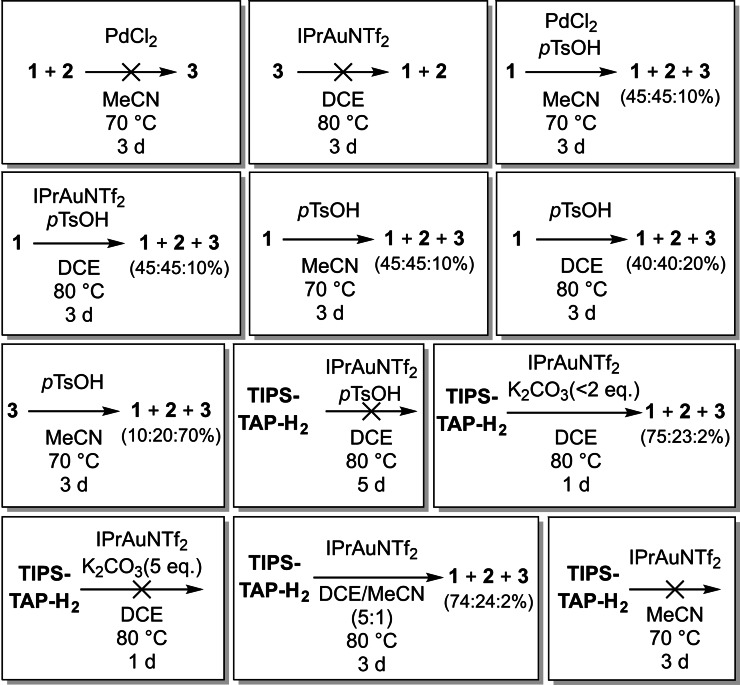
Control experiments for the silyl group shifts.

To further investigate the silyl migration, several control experiments were conducted. When **1**–**3** are treated under the optimized conditions (Scheme 4), the silyl groups of the cycloisomerized products do not shift. Adding catalytic amounts of *p*TsOH to solutions containing the catalyst and **1** under the optimized conditions results in a partial silyl shift to **2** and **3** (ratio **1 : 2 : 3** ∼45 : 45 : 10). Interestingly, the same reactivity can be induced by utilizing just *p*TsOH resulting in a similar product distribution (ratio **1 : 2**:**3**∼45 : 45 : 10 to 40 : 40 : 20, depending on the conditions; partial decomposition). Treatment of **3** with *p*TsOH results in a partial silyl shift to **1** and **2** (ratio **1 : 2 : 3** ∼10 : 20 : 70; partial decomposition). However, cycloisomerization of TIPS‐TAP‐H_2_ does not occur with *p*TsOH (Table 1, entry 20). If *p*TsOH is used as an additive for the gold‐catalyzed cyclization of TIPS‐TAP‐H_2_, the reaction is inhibited; mainly the oxidation product TIPS‐TAP and only trace amounts of the desired products can be detected. Adding base (20–200 mol% K_2_CO_3_) has no significant influence on the reaction. Only when a large excess of base is used, the reaction is inhibited, probably due to binding of the proton necessary for protodeauration. Adding MeCN as a co‐solvent to the IPrAuNTf_2_ conditions slows the reaction down (7 d instead of 1 d necessary for full conversion), although no significant difference in the product distribution can be observed. With pure MeCN as solvent only trace amounts of product along with unidentified side products are detected.

Obviously, after the protodemetallation step, the metal catalyst has no influence on the distribution of the isomers. However during the cyclization, it seems that only the catalyst determines the selectivity of the reaction while isomerization by acid after this step only delivers mixtures of different regioisomers.

**Scheme 5:**
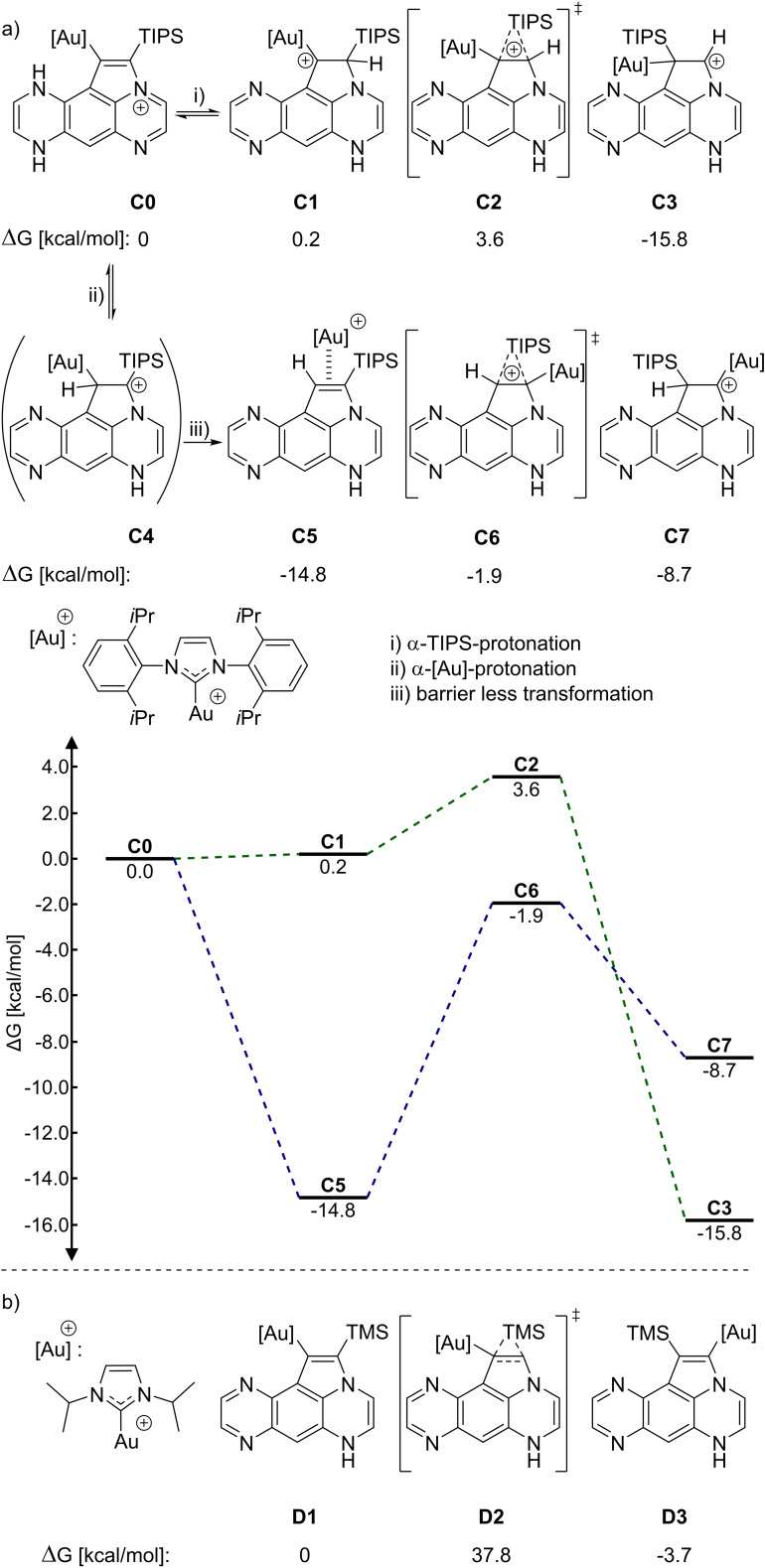
a) Silyl shift with protonation: calculated Gibbs energies (top)/reaction profile (bottom); b) silyl shift without protonation: calculated Gibbs energies (Orca 4.10,^[22]^ PBE−D3/def2‐SV(P), def2‐TZVPP effective‐core‐potentials on Gold and def2‐TZVPP on the alkyne moiety).

To get a more detailed insight into the mechanism and the catalyst dependency of the TIPS shift, quantum chemical calculations were conducted. By comparing the relative stabilities of the isomers **1**, **2** and **3**, the doubly shifted isomer is the most stable of the three isomers (ΔG compared to **1** [kcal/mol]: **2**=−5.20; **3**=−12.0) although, as seen in the experiments (Scheme 5), under thermodynamic control (acidic conditions/*p*TsOH) **3** is not formed exclusively.

Calculations using a model system with IPrAuNTf_2_ as catalyst (Orca 4.10,^[22]^ PBE‐D3/def2‐SV(P), def2‐TZVPP effective‐core‐potentials on Gold and def2‐TZVPP on the alkyne moiety) reveal two possible pathways for the TIPS shift (Scheme 5a). In line with the experimental results the barrier for protonation of the α‐TIPS‐carbon atom (**C1**) followed by a TIPS shift via a silicon bridged cationic transition state is energetically higher compared to the protonation of the α‐Au‐carbon (**C4**), which then leads to a barrierless transformation to a gold π‐complex and therefore preferring the non‐(less‐)shifted isomer **1** (**2**). A silyl shift starting from a neutral species **D1** shows a high barrier (37.8 kcal/mol) and is therefore unlikely (Scheme 5b). These results suggest that a silyl shift is only possible starting from a protonated species, either after the protonation step of the protodemetallation or through protonation after the cyclization, if the environment is acidic enough, which corresponds to the experimental observations.^[23]^

In conclusion, a new synthetic approach to bissilylated cyclopenta[*fg*,*qr*]pentacenes **1**–**3** was developed with readily available TIPS‐TAP‐H_2_ as starting material. Gold(I) catalysis resulted in **1** and **2** where either none or one silyl group is shifted on the five‐membered ring. PdCl_2_ as catalyst results in **3** in which two silyl groups are shifted. Regioisomerism does not affect optoelectronics but influences packing motifs and solubility of the species. Our combined experimental and theoretical approach showed that dihydrodiazaacenes are not able to undergo cyclizations under these conditions ‐ a pyrazinic feature is required. The selectivity and the mechanism of the reaction were investigated by quantum‐chemical calculations and highlighted the influence of the thermodynamic ratio of the intermediates **B1** and **B2** on the observed product distribution as well as the significance of the involvement of a protonated species within a possible reaction mechanism. Oxidation of **3** to the corresponding tetraazapentacene dication **3^2+^** was not successful, most likely due to the formal generation of two antiaromatic cyclopentadienyl‐type cations, a structural motif to be avoided when synthesis of cationic azaacenes is attempted. We are currently expanding the scope of our reported cyclization and investigate post‐functionalization of halogenated compounds which could be attractive semiconductors.

Data related to this article are available via heiDATA, the institutional research data repository of Heidelberg University, under the following DOI:10.11588/data/PZGMSS

### Deposition numbers

2122131 (for 1), 2122132 (for 2), 2122133 (for 3) and 2122134 (for 4) contain the supplementary crystallographic data for this paper. These data are provided free of charge by the joint Cambridge Crystallographic Data Centre and Fachinformationszentrum Karlsruhe Access Structures service.

## Conflict of interest

The authors declare no conflict of interest.

1

## Supporting information

As a service to our authors and readers, this journal provides supporting information supplied by the authors. Such materials are peer reviewed and may be re‐organized for online delivery, but are not copy‐edited or typeset. Technical support issues arising from supporting information (other than missing files) should be addressed to the authors.

Supporting Information

## Data Availability

After Acception of the manuscript the raw data of all expermients will uploaded on HeiData an university intern data repository (DOI will be avaible).

## References

[1] S. Miao , A. L. Appleton , N. Berger , S. Barlow , S. R. Marder , K. I. Hardcastle , U. H. F. Bunz , Chem. Eur. J. 2009, 15, 4990–4993. 19338039 10.1002/chem.200900324

[2] For an selected overview of metal-catalyzed cycloisomerization reactions of heteroatom-functionalized alkynes:

[2a] C. M. Hendrich , K. Sekine , T. Koshikawa , K. Tanaka , A. S. K. Hashmi , Chem. Rev. 2021, 121, 9113–9163; 33315377 10.1021/acs.chemrev.0c00824

[2b] C. Praveen , Chem. Rec. 2021, 21, 1697–1737; 34061426 10.1002/tcr.202100105

[2c] L.-H. Chung , C.-F. Yeung , C.-Y. Wong , Chem. Eur. J. 2020, 26,6102–6112; 31943425 10.1002/chem.201905506

[2d] L.-X. Wang , Y.-L. Tang , Eur. J. Org. Chem. 2017, 16, 2207–2213;

[2e] R. Mancuso , B. Gabriele , Molecules 2014, 19, 15687–15719; 25268722 10.3390/molecules191015687 PMC6271676

[2f] G. Fanga , X. Bi , Chem. Soc. Rev. 2015, 44, 8124–8173; 26222839 10.1039/c5cs00027k

[2g] A. V. Gulevich , A. S. Dudnik , N. Chernyak , V. Gevorgyan , Chem. Rev. 2013, 113, 3084–3213; 23305185 10.1021/cr300333u PMC3650130

[2h] L. Huang , M. Arndt , K. Gooßen , H. Heydt , L. J. Gooßen , Chem. Rev. 2015, 115, 2596–2697; 25721762 10.1021/cr300389u

[2i] G. Zeni , R. C. Larock , Chem. Rev. 2004, 104, 2285–2309. 15137792 10.1021/cr020085h

[3] M. Layek , A. V. D. Rao , V. Gajare , D. Kalita , D. K. Barange , A. Islam , K. Mukkanti , M. Pal , Tetrahedron Lett. 2009, 50, 4878–4881.

[4a] D. Rambabu , S. Srinivas , S. Basavoju , M. Layek , M. V. B. Rao , M. Pal , Mol. Cryst. Liq. Cryst. 2013, 577, 143–152;

[4b] M. Layek , A. Reddy M , A. V. D. Rao , M. Alvala , M. K. Arunasree , A. Islam , K. Mukkanti , J. Iqbal , M. Pal , Org. Biomol. Chem. 2011, 9, 1004–1007; 21180745 10.1039/c0ob00771d

[4c] M. J. Mphahlele , F. A. Oyeyiola , Turk. J. Chem. 2015, 39, 1216–1231.

[5] I. V. Seregin , A. W. Schammel , V. Gevorgyan , Org. Lett. 2007, 9, 3433–3436. 17637023 10.1021/ol701464j PMC2525811

[6] A. E. J. Herbert , M. Tomlinson , J. Chem. Soc. 1958, 4492–4494.

[7a] B. M. Jang, J. D. Yoo, J. G. Park, WO 2021153931, **2021**;

[7b] D. S. Choi, KR 2175379, **2020**;

[7c] N. Takahashi, Y. Iizumi, JP 2000260565, **2000**.

[8] A. Naibi Lakshminarayana , J. Chang , J. Luo , B. Zheng , K.-W. Huang , C. Chi , Chem. Commun. 2015, 51, 3604–3607. 10.1039/c4cc09812a 25634022

[9] X. Yang , M. Hoffmann , F. Rominger , T. Kirschbaum , A. Dreuw , M. Mastalerz , Angew. Chem. 2019, 131, 10760–10764; 10.1002/anie.201905666 31125478

[10a] L. Wang , Y. Han , J. Zhang , X. Li , X. Liu , J. Xiao , Org. Lett. 2020, 22, 1, 261–264; 31849229 10.1021/acs.orglett.9b04246

[10b] X. Deng , X. Liu , L. Wei , T. Ye , X. Yu , C. Zhang , J. Xiao , J. Org. Chem. 2021, 86, 15, 9961–9969; 10.1021/acs.joc.1c00332 34279110

[10c] L. Wei , X. Deng , X. Yu , X. Li , W. Wang , C. Zhang , J. Xiao , J. Org. Chem. 2021, 86, 17535–17542. 34643389 10.1021/acs.joc.1c00989

[11a] S. R. Bheemireddy , P. C. Ubaldo , P. W. Rose , A. D. Finke , J. Zhuang , L. Wang , K. N. Plunkett , Angew. Chem. 2015, 127, 15988–15992; 10.1002/anie.201508650 26768696

[11b] W. N. Lipscomb , J. M. Robertson , M. G. Rossmann , J. Chem. Soc. 1959, 2601–2607;

[11c] L. T. Scott , A. Necula , Tetrahedron Lett. 1997, 38, 1877–1880;

[11d] H. Dang , M. Levitus , M. A. Garcia-Garibay , J. Am. Chem. Soc. 2002, 124, 136–143; 11772070 10.1021/ja016189b

[11e] C. Lutke Eversloh , Y. Avlasevich , C. Li , K. Mullen , Chem. Eur. J. 2011, 17, 12756–12762. 21956345 10.1002/chem.201101126

[12] S. Dong , T. S. Herng , T. Y. Gopalakrishna , H. Phan , Z. L. Lim , P. Hu , R. D. Webster , J. Ding , C. Chi , Angew. Chem. 2016, 128, 9462–9466; 10.1002/anie.201603135 27356244

[13a] I. V. Seregin , V. Gevorgyan , J. Am. Chem. Soc. 2006, 128, 12050–12051; 16967938 10.1021/ja063278l PMC2523260

[13b] A. S. Dudnik , Y. Xia , Y. Li , V. Gevorgyan , J. Am. Chem. Soc. 2010, 132, 7645–7655; 20476771 10.1021/ja910290c PMC2896962

[13c] P. McGee , G. Bellavance , I. Korobkov , A. Tarasewicz , L. Barriault , Chem. Eur. J. 2015, 21, 9662–9665. 26037504 10.1002/chem.201501648

[14a] Q. Tang , J. Liu , H. S. Chan , Q. Miao , Chem. Eur. J. 2009, 15, 3965–3969; 19263453 10.1002/chem.200900160

[14b] Q. Miao , Synlett 2012, 23, 326–336.

[15] Gaussian 16, Revision C.01, M. J. Frisch, G. W. Trucks, H. B. Schlegel, G. E. Scuseria, M. A. Robb, J. R. Cheeseman, G. Scalmani, V. Barone, G. A. Petersson, H. Nakatsuji, X. Li, M. Caricato, A. V. Marenich, J. Bloino, B. G. Janesko, R. Gomperts, B. Mennucci, H. P. Hratchian, J. V. Ortiz, A. F. Izmaylov, J. L. Sonnenberg, D. Williams-Young, F. Ding, F. Lipparini, F. Egidi, J. Goings, B. Peng, A. Petrone, T. Henderson, D. Ranasinghe, V. G. Zakrzewski, J. Gao, N. Rega, G. Zheng, W. Liang, M. Hada, M. Ehara, K. Toyota, R. Fukuda, J. Hasegawa, M. Ishida, T. Nakajima, Y. Honda, O. Kitao, H. Nakai, T. Vreven, K. Throssell, J. A. Montgomery, Jr., J. E. Peralta, F. Ogliaro, M. J. Bearpark, J. J. Heyd, E. N. Brothers, K. N. Kudin, V. N. Staroverov, T. A. Keith, R. Kobayashi, J. Normand, K. Raghavachari, A. P. Rendell, J. C. Burant, S. S. Iyengar, J. Tomasi, M. Cossi, J. M. Millam, M. Klene, C. Adamo, R. Cammi, J. W. Ochterski, R. L. Martin, K. Morokuma, O. Farkas, J. B. Foresman, D. J. Fox, Gaussian, Inc., Wallingford CT, **2016**.

[16] J. E. Anthony , D. L. Eaton , S. R. Parkin , Org. Lett. 2002, 4, 15–18. 11772079 10.1021/ol0167356

[17] P. von Ragué Schleyer , C. Maerker , A. Dransfeld , H. Jiao , N. J. R. van Eikema Hommes , J. Am. Chem. Soc. 1996, 118, 6317–6318. 28872872 10.1021/ja960582d

[18a] D. Geuenich , R. Herges , J. Phys. Chem. A 2001, 105, 3214–3220;

[18b] D. Geuenich , K. Hess , F. Koehler , R. Herges , Chem. Rev. 2005, 105, 3758–3772. 16218566 10.1021/cr0300901

[19a] R. Breslow , Acc. Chem. Res. 1973, 6, 393–398;

[19b] K. B. Wiberg , Chem. Rev. 2001, 101, 1317–1332. 11710223 10.1021/cr990367q

[20] J. U. Engelhart , B. D. Lindner , O. Tverskoy , F. Rominger , U. H. F. Bunz , Chem. Eur. J. 2013, 19, 15089–15092. 24123393 10.1002/chem.201303277

[21] E. Epifanovsky , A. T. B. Gilbert , X. Feng , J. Lee , Y. Mao , N. Mardirossian , P. Pokhilko , A. F. White , M. P. Coons , A. L. Dempwolff , Z. Gan , D. Hait , P. R. Horn , L. D. Jacobson , I. Kaliman , J. Kussmann , A. W. Lange , K. U. Lao , D. S. Levine , J. Liu , S. C. McKenzie , A. F. Morrison , K. D. Nanda , F. Plasser , D. R. Rehn , M. L. Vidal , Z.-Q. You , Y. Zhu , B. Alam , B. J. Albrecht , A. Aldossary , E. Alguire , J. H. Andersen , V. Athavale , D. Barton , K. Begam , A. Behn , N. Bellonzi , Y. A. Bernard , E. J. Berquist , H. G. A. Burton , A. Carreras , K. Carter-Fenk , R. Chakraborty , A. D. Chien , K. D. Closser , V. Cofer-Shabica , S. Dasgupta , M. de Wergifosse , J. Deng , M. Diedenhofen , H. Do , S. Ehlert , P.-T. Fang , S. Fatehi , Q. Feng , T. Friedhoff , J. Gayvert , Q. Ge , G. Gidofalvi , M. Goldey , J. Gomes , C. E. González-Espinoza , S. Gulania , A. O. Gunina , M. W. D. Hanson-Heine , P. H. P. Harbach , A. Hauser , M. F. Herbst , M. Hernández Vera , M. Hodecker , Z. C. Holden , S. Houck , X. Huang , K. Hui , B. C. Huynh , M. Ivanov , Á. Jász , H. Ji , H. Jiang , B. Kaduk , S. Kähler , K. Khistyaev , J. Kim , G. Kis , P. Klunzinger , Z. Koczor-Benda , J. H. Koh , D. Kosenkov , L. Koulias , T. Kowalczyk , C. M. Krauter , K. Kue , A. Kunitsa , T. Kus , I. Ladjánszki , A. Landau , K. V. Lawler , D. Lefrancois , S. Lehtola , R. R. Li , Y.-P. Li , J. Liang , M. Liebenthal , H.-H. Lin , Y.-S. Lin , F. Liu , K.-Y. Liu , M. Loipersberger , A. Luenser , A. Manjanath , P. Manohar , E. Mansoor , S. F. Manzer , S.-P. Mao , A. V. Marenich , T. Markovich , S. Mason , S. A. Maurer , P. F. McLaughlin , M. F. S. J. Menger , J.-M. Mewes , S. A. Mewes , P. Morgante , J. W. Mullinax , K. J. Oosterbaan , G. Paran , A. C. Paul , S. K. Paul , F. Pavošević , Z. Pei , S. Prager , E. I. Proynov , Á. Rák , E. Ramos-Cordoba , B. Rana , A. E. Rask , A. Rettig , R. M. Richard , F. Rob , E. Rossomme , T. Scheele , M. Scheurer , M. Schneider , N. Sergueev , S. M. Sharada , W. Skomorowski , D. W. Small , C. J. Stein , Y.-C. Su , E. J. Sundstrom , Z. Tao , J. Thirman , G. J. Tornai , T. Tsuchimochi , N. M. Tubman , S. P. Veccham , O. Vydrov , J. Wenzel , J. Witte , A. Yamada , K. Yao , S. Yeganeh , S. R. Yost , A. Zech , I. Y. Zhang , X. Zhang , Y. Zhang , D. Zuev , A. Aspuru-Guzik , A. T. Bell , N. A. Besley , K. B. Bravaya , B. R. Brooks , D. Casanova , J.-D. Chai , S. Coriani , C. J. Cramer , G. Cserey , A. E. DePrince , R. A. DiStasio , A. Dreuw , B. D. Dunietz , T. R. Furlani , W. A. Goddard , S. Hammes-Schiffer , T. Head-Gordon , W. J. Hehre , C.-P. Hsu , T.-C. Jagau , Y. Jung , A. Klamt , J. Kong , D. S. Lambrecht , W. Liang , N. J. Mayhall , C. W. McCurdy , J. B. Neaton , C. Ochsenfeld , J. A. Parkhill , R. Peverati , V. A. Rassolov , Y. Shao , L. V. Slipchenko , T. Stauch , R. P. Steele , J. E. Subotnik , A. J. W. Thom , A. Tkatchenko , D. G. Truhlar , T. van Voorhis , T. A. Wesolowski , K. B. Whaley , H. L. Woodcock , P. M. Zimmerman , S. Faraji , P. M. W. Gill , M. Head-Gordon , J. M. Herbert , A. I. Krylov , J. Chem. Phys. 2021, 155, 84801.

[22] F. Neese , WIREs Comput. Mol. Sci. 2012, 2, 73–78.

[23] Preliminary investigations of a possible reaction mechanism involving PdCl_2_ were not yet met with success due to difficulties determining the reactive species. Further investigation is set for following publications.

