# Supplementary material for: Cyclopentannulated Dihydrotetraazapentacenes
Source: Chemistry. 2022 Feb 2;28(12):e202104203. doi: 10.1002/chem.202104203 (PMC9303544; doi:10.1002/chem.202104203)
Supplement: Supplementary file 1 — Supporting Information [file CHEM-28-0-s001.pdf]

Chemistry–A European Journal

Supporting Information

Cyclopentannulated Dihydrotetraazapentacenes

Robin Heckershoff, Steffen Maier, Thomas Wurm, Philipp Biegger, Kerstin Brödner, Petra Krämer, Marvin T. Hoffmann, Lukas Eberle, Jana Stein, Frank Rominger, Matthias Rudolph, Jan Freudenberg,* Andreas Dreuw,* A. Stephen K. Hashmi,* and Uwe H. F. Bunz*

Table of Contents

1 Experimental Procedures ... 2
1.1 General Information ... 2
1.2 Synthesis of Compounds ... 3
1.3 Catalyst Screening ... 6
2 NMR Spectra ... 7
3 UV-Vis Spectra ... 12
4 Crystallographic Data ... 14
5 Computational Details ... 18
5.1 Mechanism of the Cycloisomerization ... 18
5.1.1 Cyclisation Mode Through the Pyrazine-Substructure ... 18
5.1.2 Calculated Thermodynamic Data ... 19
5.1.3 Coordinates of the Optimized Geometries ... 20
5.2 Calculations of the Optoelectronic Properties ... 59
5.2.1 FMO Calculations ... 59
5.2.2 Calculated and Measured Bond Lengths ... 59
5.2.3 Coordinates of the Optimized Geometries ... 60
6 References ... 69

1 Experimental Procedures

1.1 General Information

Chemicals were bought from commercial suppliers (ABCR, Acros, Alfa Aesar, Carbolution, Chempur, Fluka, Merck, Sigma Aldrich and TCI) and used as delivered. Anhydrous solvents were dispensed from a solvent purification system MB SPS-800. Deuterated solvents were bought from Euriso Top and Sigma Aldrich.
Melting points (mp) were measured in open glass capillaries on a Stuart SMP10 melting point apparatus and are uncorrected.
R_f-values were determined by analytical thin layer chromatography (TLC) on aluminum sheets coated with silica gel produced by Macherey-Nagel (ALUGRAM® Xtra SIL G/25 UV_{254}). Detection was accomplished using UV-light (254 and 365 nm) or a TLC staining solution (vanillin).
Nuclear magnetic resonance (NMR) spectra were recorded at the chemistry department of Heidelberg University under the supervision of Dr. J. Graf on the following spectrometers: Bruker Avance III 300 (300 MHz), Bruker Avance DRX 300 (300 MHz), Bruker Fourier 300 (300 MHz), Bruker Avance III 400 (400 MHz), Bruker Avance III 500 (500 MHz) and Bruker Avance III 600 (600 MHz). $CDCl_3$ was filtered through a plug of aluminum oxide (ALOX) prior to use to remove acid impurities. Chemical shifts (δ) are given in ppm and coupling constants *J* in Hz. Spectra were referenced to residual solvent protons according to Fulmer *et al.*[1] The following abbreviations were used to describe the observed multiplicities: for ^{1}H NMR spectra: s = singlet, d = doublet, sept = septet, m = multiplet.
High-resolution mass spectra (HR-MS) were recorded at the chemistry department of Heidelberg University under the supervision of Dr. J. Gross on the following spectrometers: JEOL AccuTOF GCx (EI)**,** Bruker ApexQe hybrid 9.4 T FT-ICR (ESI, MALDI, DART), Finnigan LCQ (ESI) and Bruker AutoFlex Speed (MALDI). For MALDI, *trans*-2-[3-(4-*tert*-butylphenyl)-2-methyl-2-propenylidene]-malononitrile (DCTB) was used as matrix.
Infrared spectra were recorded from a neat powder on a FT/IR spectrometer (Bruker LUMOS or Jasco FT/IR-4100) with a Germanium ATR-crystal. For the most significant bands the wave numbers are given.
UV-Vis spectra were recorded on a Jasco UV-VIS V-670. Fluorescence spectra were recorded on a Jasco FP6500. Quantumyields (QY) were recorded on a Jasco FP-8600 fluorescence spectrometer equipped with a ILF-835 100 mm dia. integrating sphere.
X-ray crystallography was carried out at the chemistry department of Heidelberg University under the supervision of Dr. F. Rominger on the following instruments: Bruker Smart APEX II Quazar (with Mo-microsource) and Stoe Stadivari (with Co-microsource and Pilatus detector).
HPLC was carried out on an Agilent 1100 series HPLC system (column: Merck LiChrosorb® Si 60 (5 µm); solvent: hexane to hexane:DCM = 1:1, linear gradient; flow rate: 1 mL/min; UV detection at 254 nm). For flash column chromatography silica gel (Sigma-Aldrich, pore size 60 Å, 70-230 mesh, 63-200 µm) was used as stationary phase. As eluents different mixtures of petroleum ether (PE), ethyl acetate (EA), DCM or MeOH were used.
All reactions were performed under air, if not otherwise specified. For handling of air and moisture sensitive reagents, standard Schlenk techniques with flame-dried glassware under an argon or nitrogen atmosphere were used.

1.2 Synthesis of Compounds

6,13-Bis((triisopropylsilyl)ethynyl)-5,14-dihydroquinoxalino[2,3-*b*]phenazine (TIPS-TAP-H$_2$), 5,-14-bis((isopropylsilyl)ethynyl)-6,13-dihydrodibenzo[*b*,*i*]phenazine (6), 7,12-bis((triisopropylsilyl)-ethynyl)-6,13-dihydrobenzo[*g*]quinoxalino[2,3-*b*]quinoxaline (7)

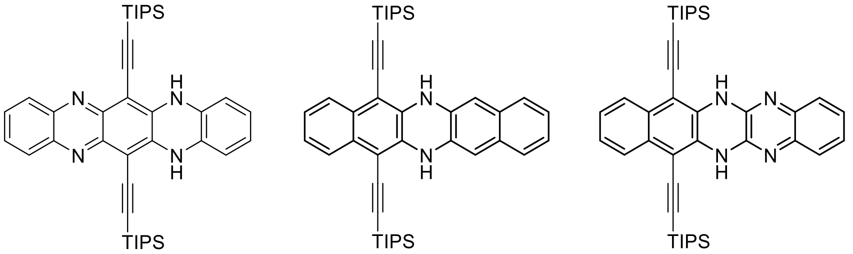

TIPS-TAP-H$_2$ **6** **7**

TIPS-TAP-H$_2$[2], **6**[3] and **7**[4] were synthesized following known procedures.

2,9-Bis(triisopropylsilyl)-2a,7,9a,14-tetraazadicyclopenta[*fg*,*qr*]pentacene (1), 2,8-bis(triisopropylsilyl)-2a,7,9a,14-tetraazadicyclopenta[*fg*,*qr*]pentacene (2)

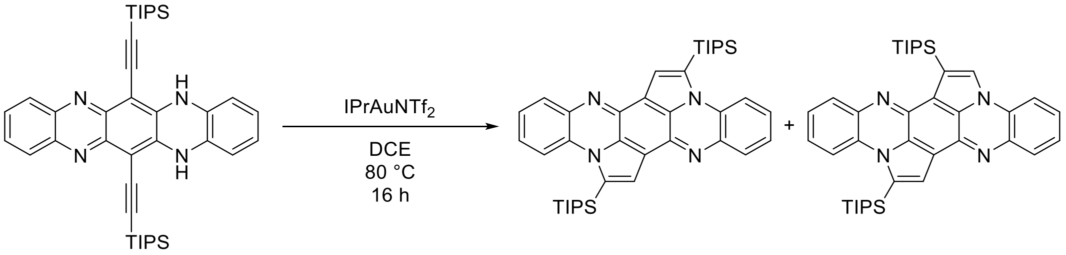

TIPS-TAP-H$_2$ **1** **2**

TIPS-TAP-H$_2$ (54.3 mg, 84.2 µmol, 1.00 eq.) and IPrAuNTf$_2$ (7.29 mg, 8.42 µmol, 0.10 eq.) were dissolved in DCE (2 mL). The reaction mixture was stirred for at 80 °C for 16 h. After cooling to room temperature, the solvent was removed under reduced pressure and the crude product was purified by flash column chromatography (silica gel, PE:EA = 40:1) to yield a mixture of the two isomers **1** and **2** (ratio ~ 3:1) as a yellow solid (50.3 mg, 78.0 µmol, 93%). To obtain the pure compounds a part of the mixture was separated by preparative HPLC (ReproSil-Pur 120 Si, PE:EA = 20:1) to yield pure **1** and **2** as yellow solids.

1:
Mp: >300 °C; **R*f***: 0.51 (silica gel, PE:EA = 10:1); **^{1}H NMR** (400 MHz, CDCl$_3$): δ = 8.07-8.01 (m, 4H), 7.87 (s, 2H), 7.51-7.43 (m, 4H), 1.74 (sept, *J* = 7.5 Hz, 6H), 1.23 (d, *J* = 7.5 Hz, 36H). **^{13}C{^{1}H} NMR** (101 MHz, CDCl$_3$): δ = 146.8, 140.4, 132.1, 130.2, 129.8, 129.5, 126.1, 126.0, 123.0, 116.7, 116.6, 19.4, 14.0; **HR-MS** (DART pos.) *m/z*: [M+H]$^+$: calcd. for [$C_{40}H_{53}N_4Si_2$]$^+$: 645.3803; found: 645.3784; **IR** (ATR): ν [cm^{-1}] = 2947, 2865, 1613, 1552, 1532, 1481, 1464, 1421, 1387, 1332, 1284, 1244, 1192, 1136, 1104, 1078, 1044, 1018, 966, 922, 881, 844, 753, 716, 671, 653, 625, 605. **UV-Vis** (DCM): λ [nm] (logε) = 389 (4.00), 410 (4.40), 435 (4.69), 466 (4.77); **fluorescence** (DCM): λ_{ex} = 342 nm, λ_{max} = 478, 507, 541 nm; **quantum yield** (DCM): Φ = 59%.

2:
Mp: >300 °C; **R*f***: 0.53 (silica gel, PE:EA = 10:1); **^{1}H NMR** (400 MHz, CDCl$_3$): δ = 8.04-8.01 (m, 2H), 7.98-7.95 (m, 1H), 7.89-7.87 (m, 3H), 7.50-7.43 (m, 4H), 2.01 (sept, *J* = 7.5 Hz, 3 H), 1.73 (sept, *J* =

7.5 Hz, 3H), 1.26-1.21 (m, 36H); **^{13}C{^{1}H} NMR** (101 MHz, $CDCl_3$): δ = 147.4, 146.5, 140.3, 139.3, 132.4, 130.3, 129.6, 129.5, 129.1, 127.2, 127.0, 126.3, 126.0, 122.7, 121.7, 118.3, 116.6, 115.8, 114.3, 19.4, 14.0, 12.5; **HR-MS** (DART pos.) *m/z*: $[M+H]^+$: calcd. for $[C_{40}H_{53}N_4Si_2]^+$: 645.3803; found: 645.3774; **IR** (ATR): ν [cm^{-1}] = 2925, 2863, 1612, 1557, 1522, 1505, 1484, 1464, 1426, 1384, 1293, 1249, 1196, 1160,1134, 1097, 1052, 1020, 952, 883, 858, 769, 750, 714, 681, 654, 624; **UV-Vis** (DCM): λ [nm] (logε) = 390 (4.04), 411 (4.38), 437 (4.66), 466 (4.75); **fluorescence** (DCM): λ_{ex} = 342 nm, λ_{max} = 478, 507, 543 nm; **quantum yield** (DCM): Φ = 58%.

1,8-Bis(triisopropylsilyl)-2a,7,9a,14-tetraazadicyclopenta[*fg*,*qr*]pentacene (3)

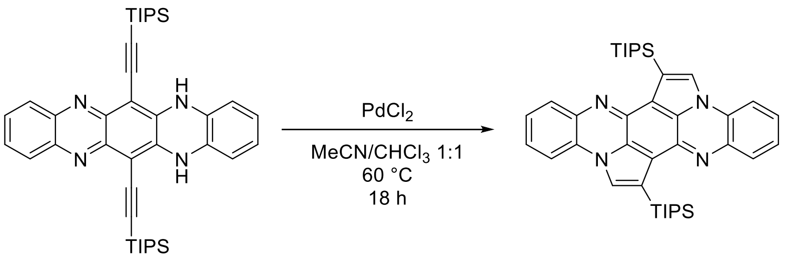

TIPS-TAP-H_2 3

TIPS-TAP-H_2 (400 mg, 620 µmol, 1.00 eq.) was dissolved in $CHCl_3$ (20 mL). Afterwards MeCN (20 mL) and palladium dichloride (11.0 mg, 62.0 µmol, 0.10 eq.) were added. The reaction mixture was stirred at 80 °C for 18 h. After cooling to room temperature the solvent was removed under reduced pressure and the crude product was purified by flash column chromatography (silica gel, PE) to yield the product as a yellow solid (310 mg, 465 µmol, 78%).

Mp: >300 °C; **R_f**: 0.93 (silica gel, PE:EA = 10:1); **^{1}H NMR** (600 MHz, $CDCl_3$): δ = 7.91-7.89 (m, 2H), 7.87-7.85 (m, 4H), 7.47-7.43 (m, 4H), 1.99 (sept, *J* = 7.5 Hz, 6H), 1.21 (d, *J* = 7.5 Hz, 36H); **^{13}C{^{1}H} NMR** (151 MHz, $CDCl_3$): δ = 147.2, 139.4, 129.9, 129.8, 126.9, 126.8, 126.0, 122.3, 120.9, 117.4, 114.1, 19.4, 12.4; **HR-MS** (DART pos.) *m/z*: $[M+H]^+$: calcd. for $[C_{40}H_{53}N_4Si_2]^+$: 645.3803; found: 645.3774; **IR** (ATR): ν [cm^{-1}] = 2954, 2922, 2856, 2341, 2336, 1653, 1558, 1499, 1488, 1464, 1457, 1427, 1378, 1194, 1157, 1133, 1111, 1098, 1093, 1082, 1077, 1073, 1054, 1030, 1019, 997, 882, 745, 678, 668, 657, 648; **UV-Vis** (DCM): λ [nm] (logε) = 391 (4.03), 413 (4.42), 439 (4.72), 466 (4.82); **fluorescence** (DCM): λ_{ex} = 259 nm, λ_{max} = 473, 507, 545 nm; **quantum yield** (DCM): Φ = 67%.

2a,7,9a,14-Tetraazadicyclopenta[*fg*,*qr*]pentacene (4)

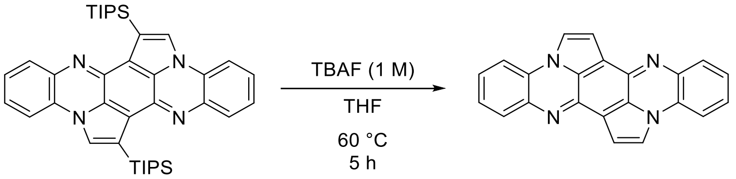

3 **4**

In a flame dried Schlenk flask **3** (60.0 mg, 93.0 µmol, 1.00 eq.) was dissolved in THF (10 mL). Tetra-*n*-butylammonium fluoride (1 M solution in THF, 930 µL, 930 µmol, 10.0 eq.) was added dropwise and the reaction mixture was stirred under nitrogen at 60 °C for 5 h. After cooling to room temperature, the solvent was removed under reduced pressure and the residue was washed with water, MeOH and a 1:1 mixture of DCM and pentane to yield the product as an orange-red solid (28.4 mg, 85.5 µmol, 92%).

Mp: >300 °C; **R*f***: 0.41 (silica gel, PE:EA = 1:1); **^{1}H NMR** (300 MHz, DMSO-d$_6$, 135 °C): δ = 8.35-8.34 (m, 2H), 8.23-8.20 (m, 2H), 7.97-7.94 (m, 2H), 7.61-7.52 (m, 4H), 7.44-7.43 (m, 2H); **HR-MS** (EI pos.) *m/z*: [M]$^+$: calcd. for [$C_{22}H_{12}N_4$]$^+$: 332.10565; found: 332.10394; **IR** (ATR): ν [cm^{-1}] = 1552, 1524, 1491, 1424, 1321, 1254, 1189, 1181, 1115, 769, 755, 742, 726, 667, 660; **UV-Vis** (DCM): λ [nm] (logε) = 385 (4.03), 407 (4.41), 431 (4.69), 460 (4.78); **fluorescence** (DCM): λ_{ex} = 406 nm, λ_{max} = 470, 500, 535 nm; **quantum yield** (DCM): Φ = 63%.

1-(Triisopropylsilyl)-7-((triisopropylsilyl)ethynyl)-8*H*-benzo[*g*]pyrrolo[1,2,3-*de*]quinoxalino[2,3-*b*]quinoxaline

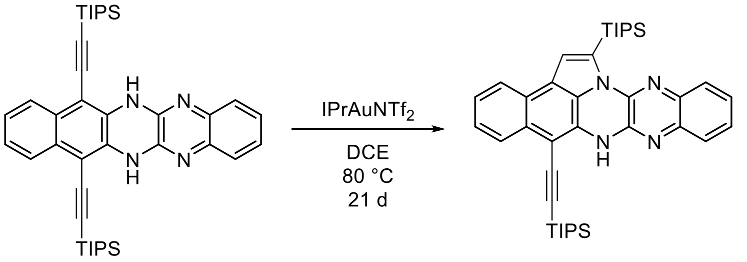

7 **10**

7 (50.0 mg, 77.5 µmol,1.00 eq.) and IPrAuNTf$_2$ (6.71 mg, 7.75 µmol, 0.10 eq.) were dissolved in DCE (5 mL). The reaction mixture was stirred at 80 °C for 21 d. After cooling to room temperature, the solvent was removed under reduced pressure and the crude product was purified by flash column chromatography (silica gel, PE:DCM = 1:1) to yield the product as an orange-red solid (18.5 mg, 28.7 µmol, 37%).

Mp: >300 °C; **R*f***: 0.25 (silica gel, PE:DCM = 1:1); **^{1}H NMR** (400 MHz, CD_2Cl_2): δ = 8.20-8.17 (m, 1H), 8.07-8.05 (m, 1H), 7.78-7.73 (m, 2H), 7.64-7.61 (m, 1H), 7.51-7.40 (m, 5H), 1.96 (sept, *J*= 7.56 Hz, 3 H), 1.28-1.23 (m, 39H); **^{13}C{^{1}H} NMR** (101 MHz, CD_2Cl_2): δ 142.2, 140.5, 138.2, 137.9, 134.6, 132.3, 131.0, 128.9, 128.1, 127.4, 126.8, 126.7, 126.3, 126.3, 125.2, 124.5, 124.1, 123.8, 120.9, 102.7, 100.6, 95.3, 19.5, 19.1, 13.9, 11.9; **HR-MS** (ESI pos.) *m/z*: [M+H]$^+$: calcd. for [$C_{40}H_{53}N_4Si_2$]$^+$: 645.38033; found: 645.37979; **IR** (ATR): ν [cm^{-1}] = 3397, 2943, 2865, 2126, 1634, 1602, 1573, 1537, 1515, 1461, 1389, 1361, 1238, 1145, 1124, 1102, 1052, 1019, 954, 903, 883, 823, 757, 703, 686, 671, 649, 622, 606.

5,14-Bis((triisopropylsilyl)ethynyl)dibenzo[*b*,*i*]phenazine (6-ox)

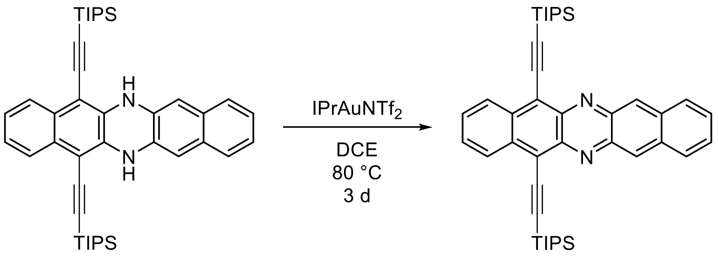

6 **6-ox**

6 (50.0 mg, 77.8 µmol,1.00 eq.) and IPrAuNTf$_2$ (6.73 mg, 7.78 µmol, 0.10 eq.) were dissolved in DCE (5 mL). The reaction mixture was stirred for at 80 °C for 3 d. After cooling to room temperature, the solvent was removed under reduced pressure and the crude product was purified by flash column chromatography (silica gel, PE:EA = 20:1) to yield the product as a green solid (48.5 mg, 75.4 µmol, 97%).

R*f*: 0.63 (silica gel, PE:EA = 9:1); **^{1}H NMR** (300 MHz, $CDCl_3$): δ = 8.86 (s, 2H), 8.74-8.71 (m, 2H), 8.07-8.03 (m, 2H), 7.63-7.60 (m, 2H), 7.41-7.38 (m, 2H), 1.40 (m, 42H).

The spectroscopic data correspond to those previously reported in the literature.[5]

1.3 Catalyst Screening

The aim of the screening was to determine the product distribution of **1**, **2**, and **3** in dependency of different catalysts. In a 4 ml screw cap vial equipped with a stirring bar **TIPS-TAP-H$_2$** (10.0 mg, 15.5 µmol) was dissolved in DCE or MeCN (2 mL) and 10 mol% of the catalyst was added. The reaction mixture was stirred at 80 °C (70 °C for MeCN) until TLC showed full conversion (detection by UV light, 365 nm, strong yellow fluorescence) or for a maximum of 7 d. After cooling to room temperature, the mixture was filtered through a plug of silica gel, eluated with DCM and the solvent was removed under reduced pressure.
A combination of HPLC and ^{1}H NMR analytics was used to determine the ratio between the substrate (**TIPS-TAP-H$_2$**) and each product (**1**, **2**, and **3**) and the occurrence of unidentified side products. Due to partly overlapping signals in the HPLC and ^{1}H NMR measurements, the HPLC data was used for the ratio of **TIPS-TAP-H$_2$**, **1**, **2** and side products while the ^{1}H NMR data allowed measuring the ratio of **3** to **1** and **2**.

2 NMR Spectra

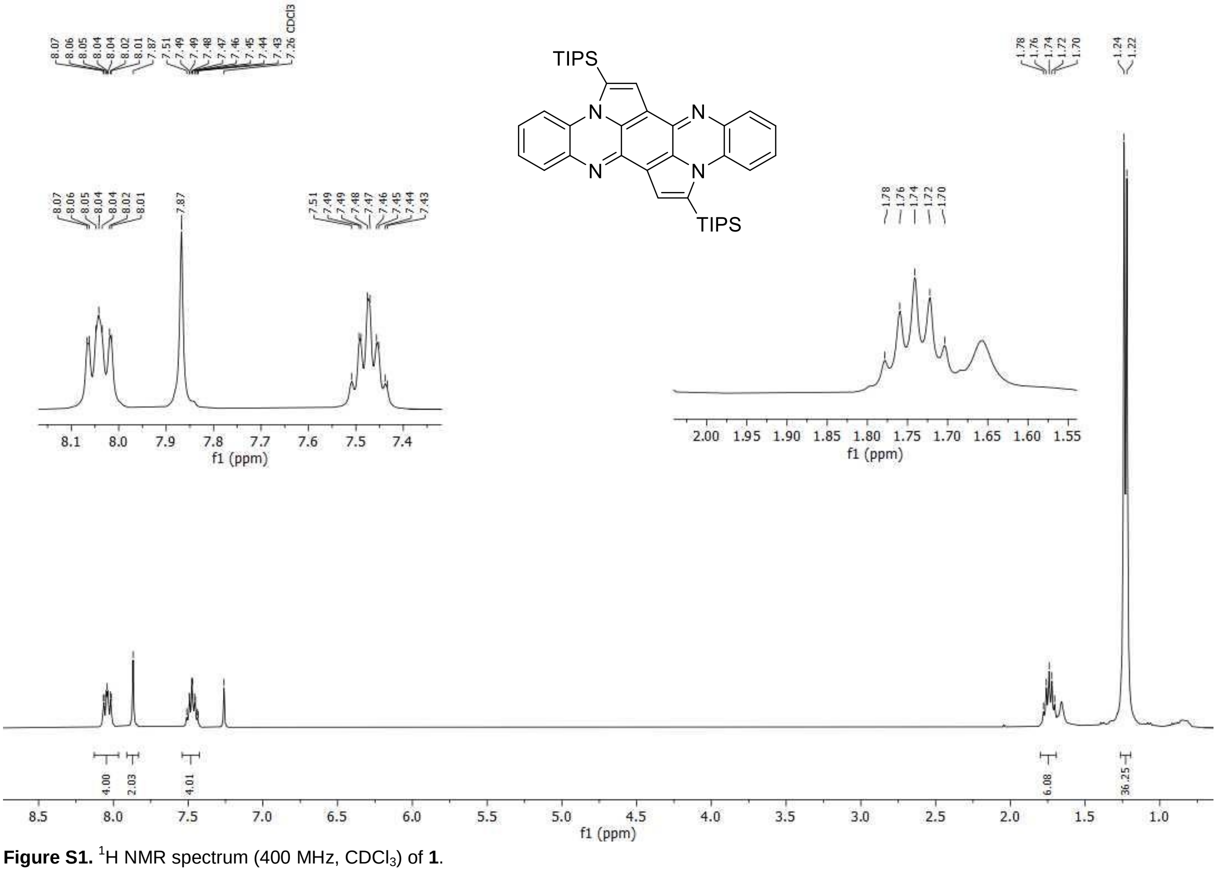

Figure S1. ^{1}H NMR spectrum (400 MHz, $CDCl_3$) of **1**.

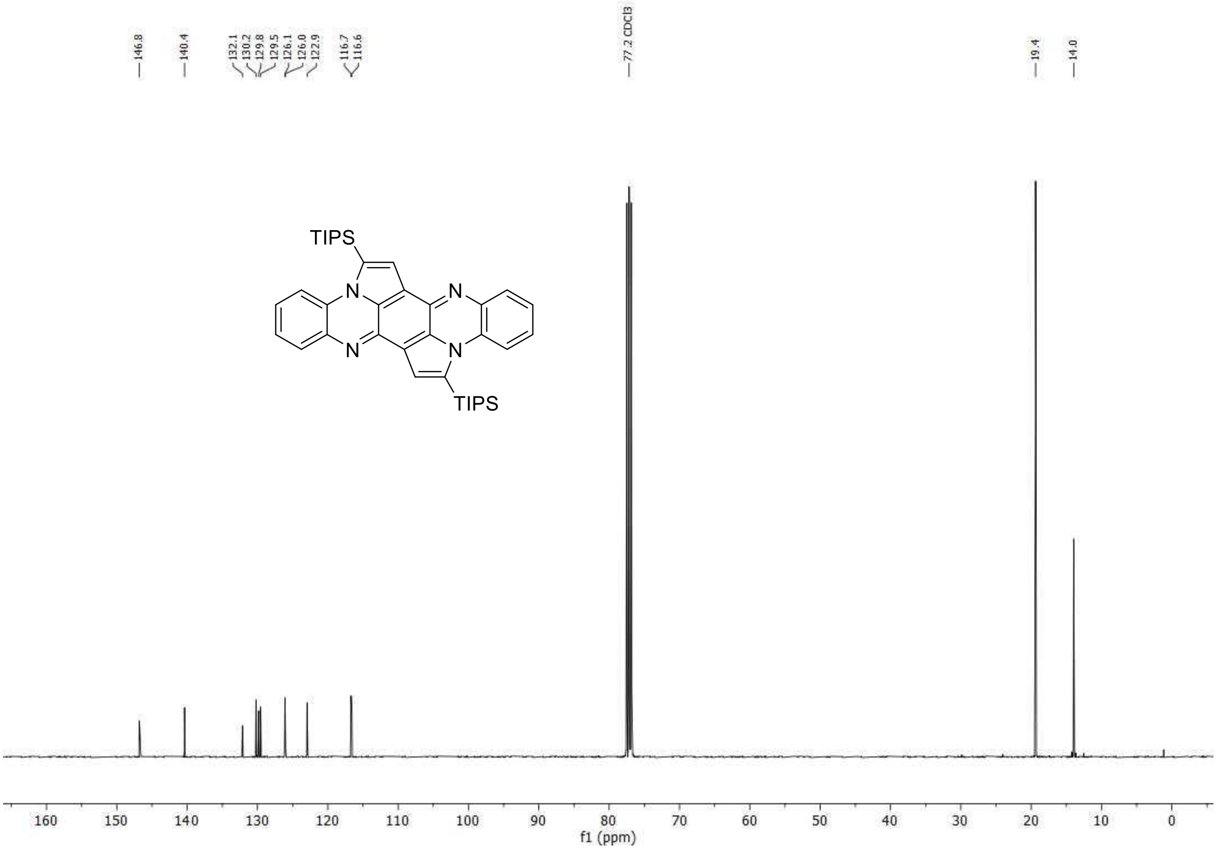

Figure S2. $^{13}C\{^{1}H\}$ NMR spectrum (101 MHz, $CDCl_3$) of **1**.

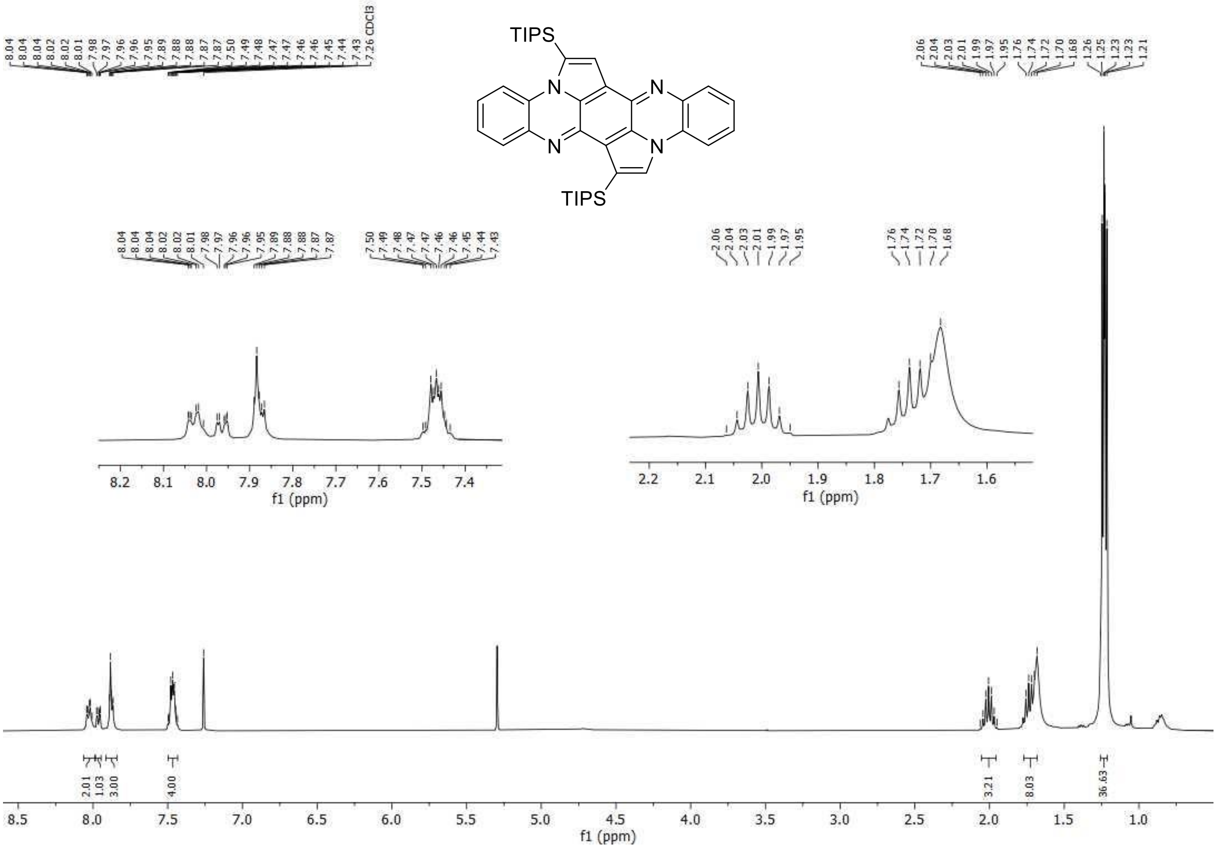

Figure S3. ^{1}H NMR spectrum (400 MHz, $CDCl_3$) of **2**.

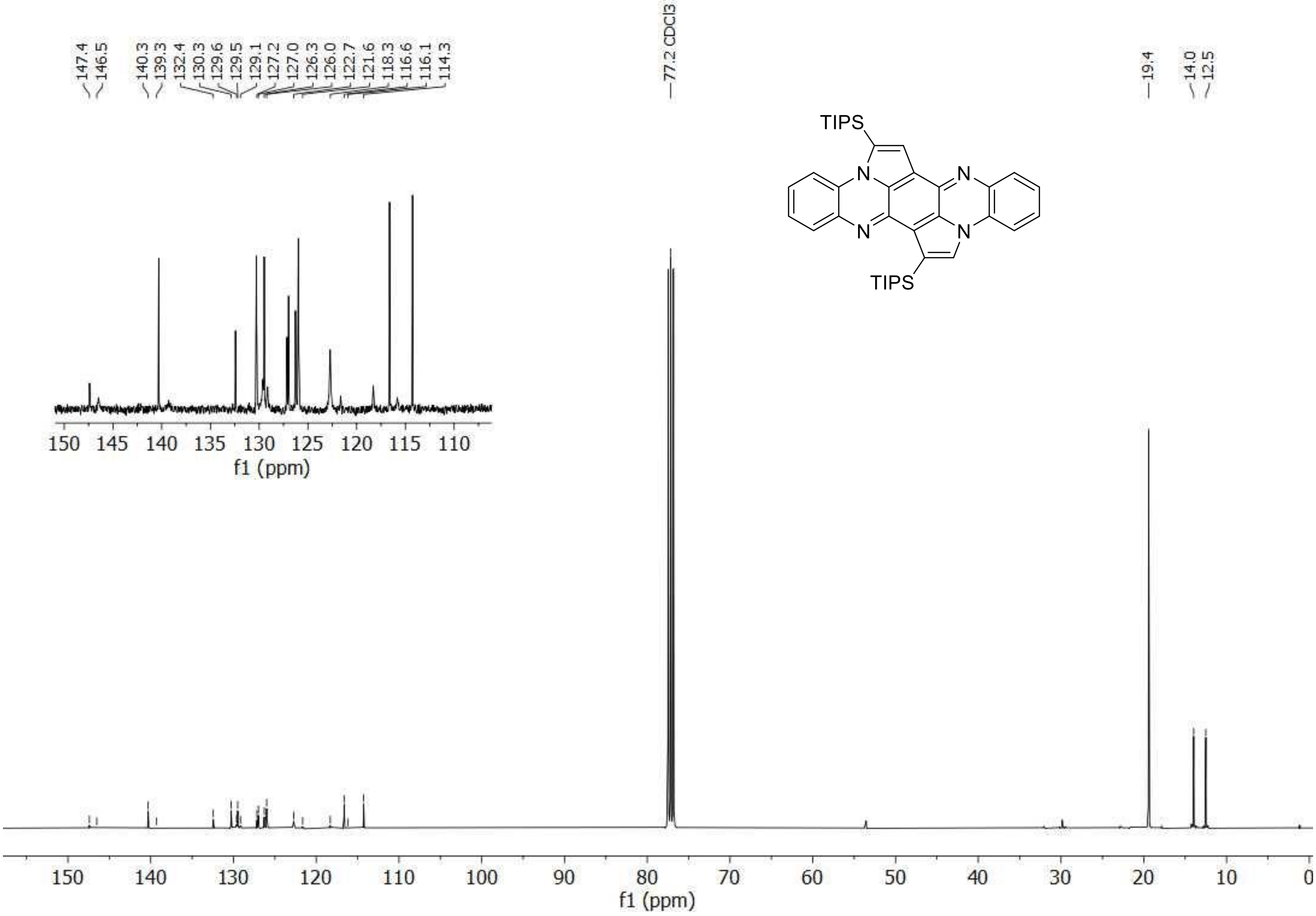

Figure S4. $^{13}C\{^{1}H\}$ NMR spectrum (151 MHz, $CDCl_3$) of **2**.

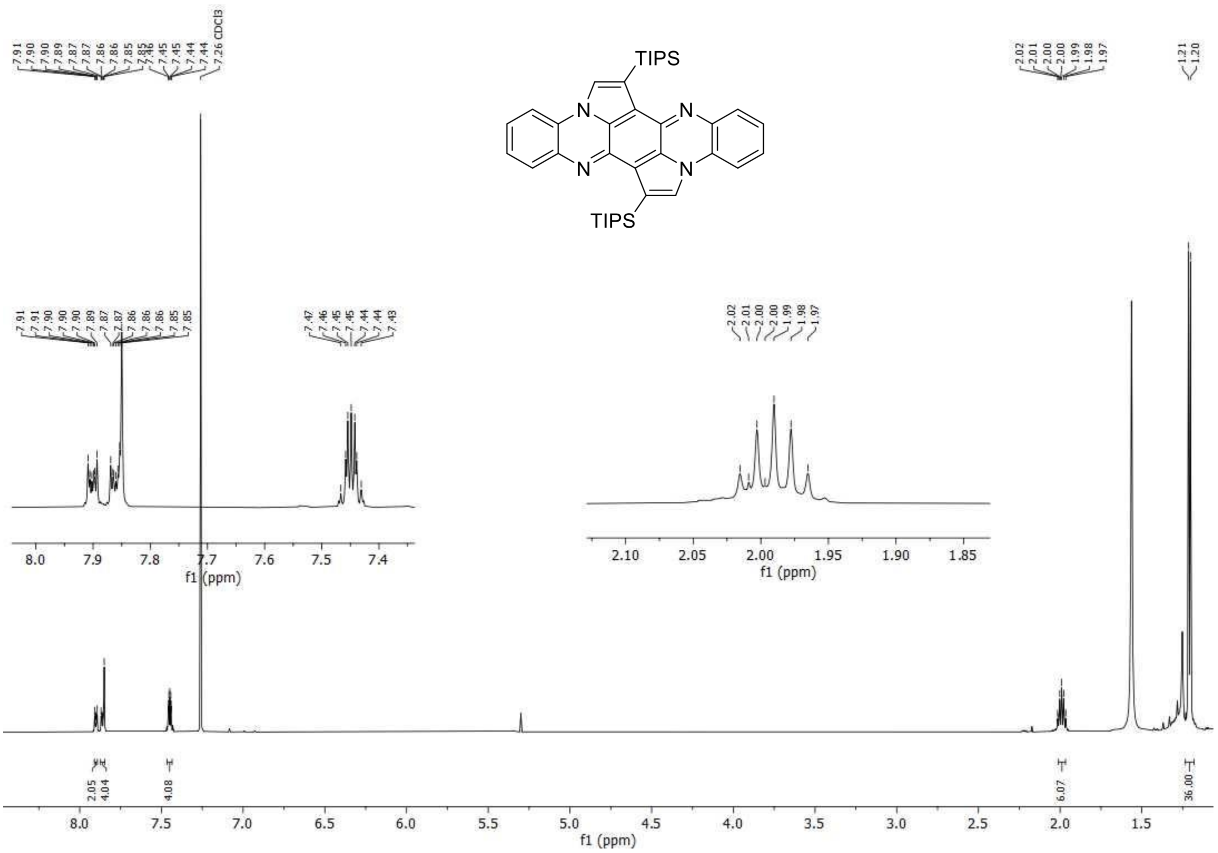

Figure S5. ^{1}H NMR spectrum (600 MHz, $CDCl_3$) of **3**.

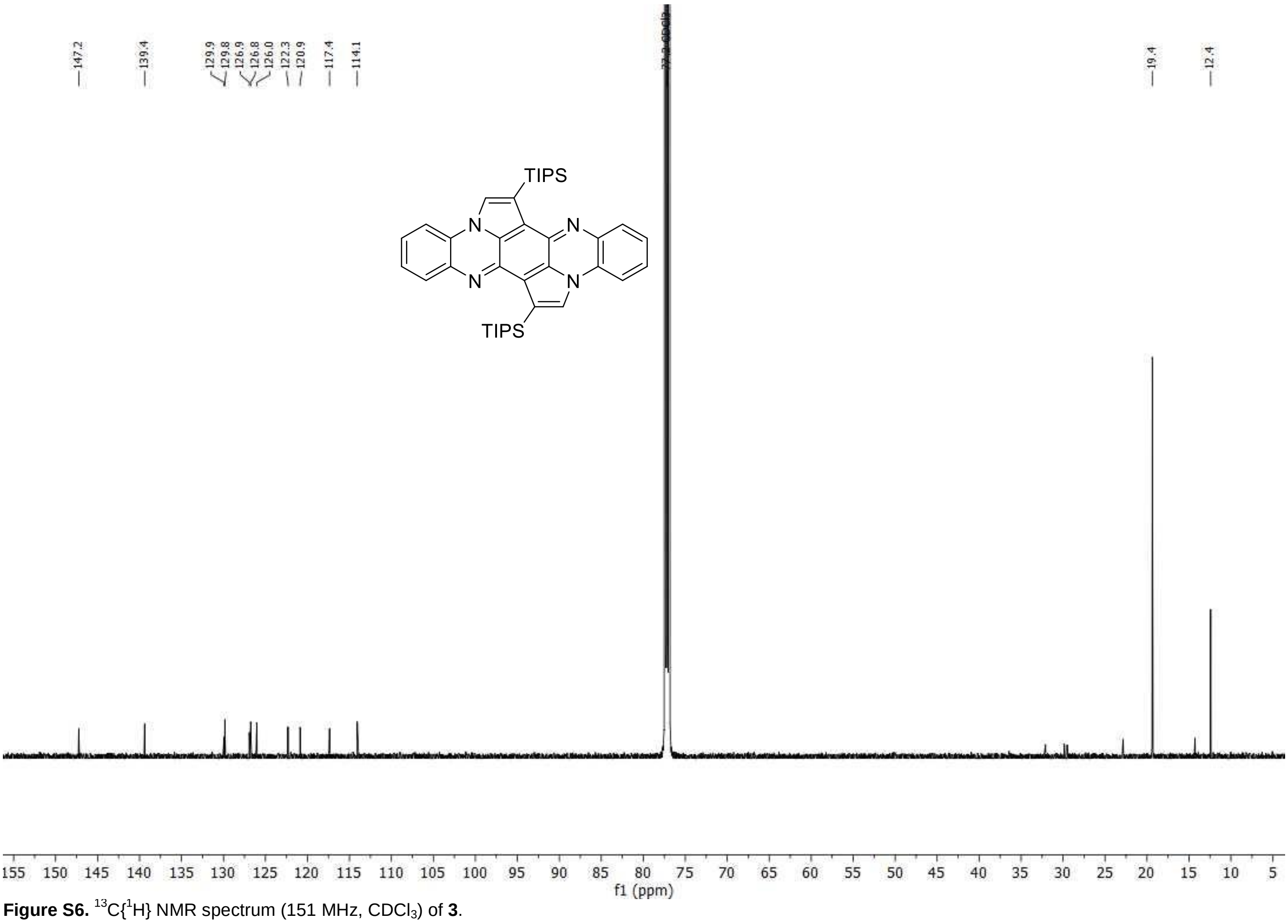

Figure S6. $^{13}C\{^{1}H\}$ NMR spectrum (151 MHz, $CDCl_3$) of **3**.

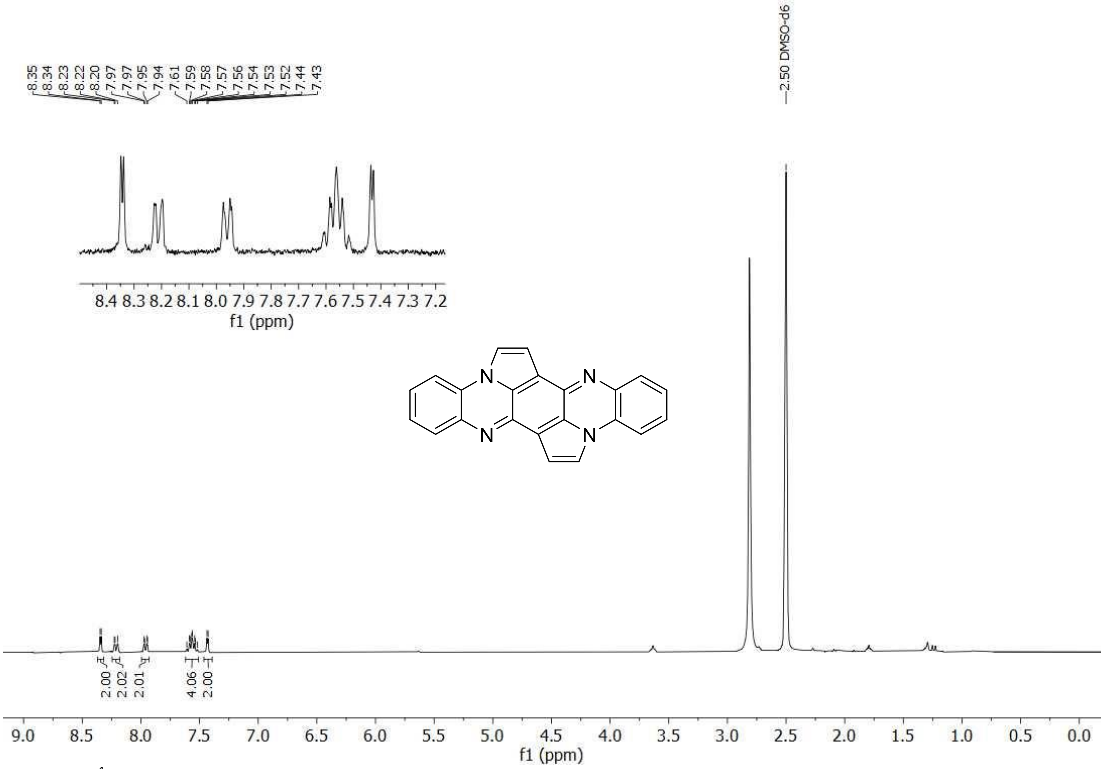

Figure S7. ^{1}H NMR spectrum (300 MHz, DMSO-d_6, 135 °C) of **4**.

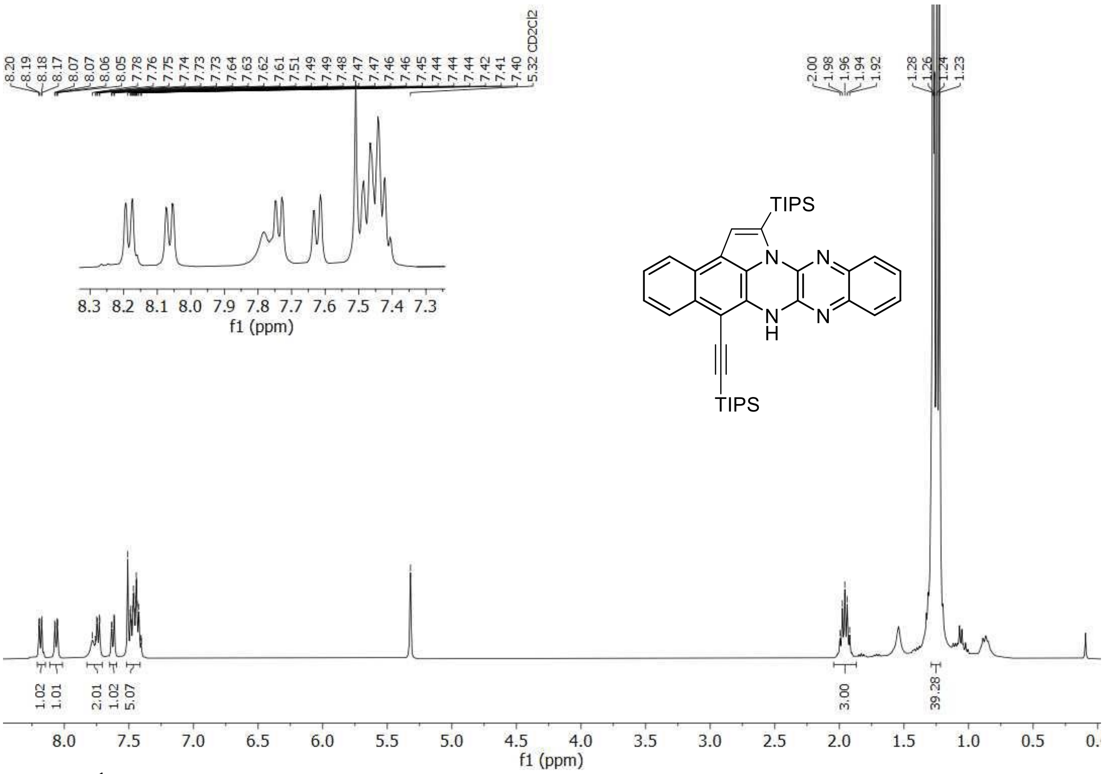

Figure S8. ^{1}H NMR spectrum (400 MHz, CD_2Cl_2) of **10**.

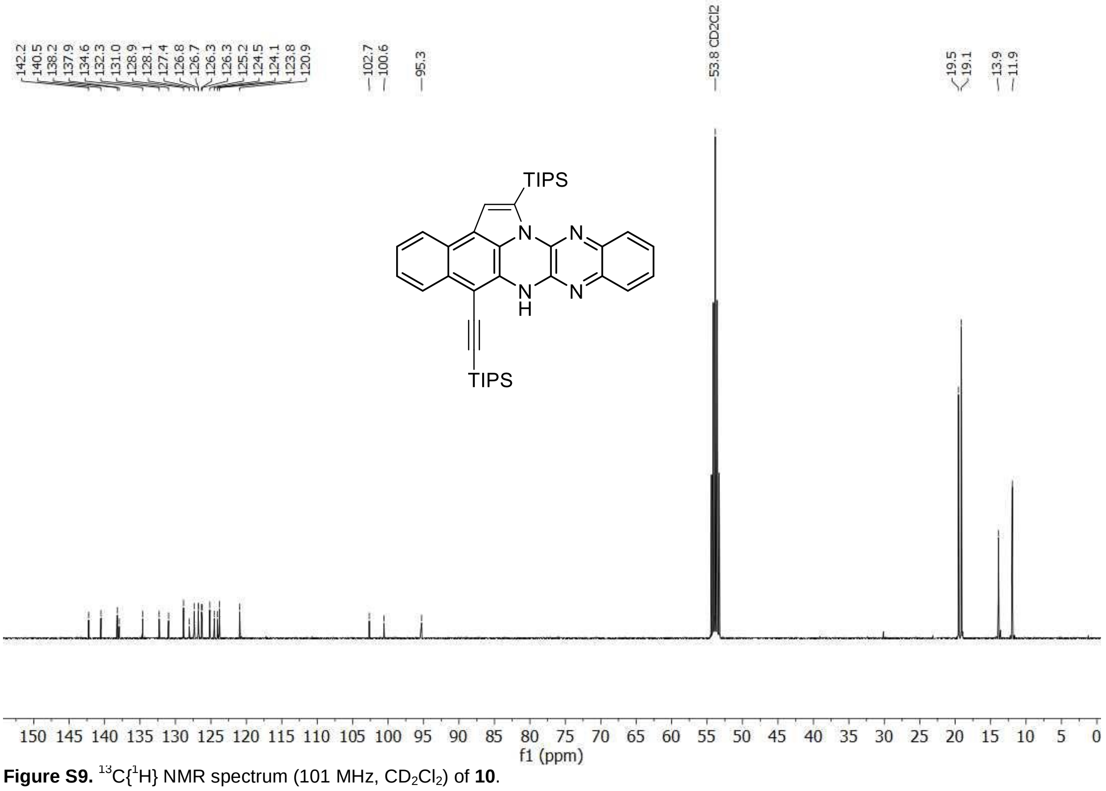

Figure S9. $^{13}C\{^1H\}$ NMR spectrum (101 MHz, CD_2Cl_2) of **10**.

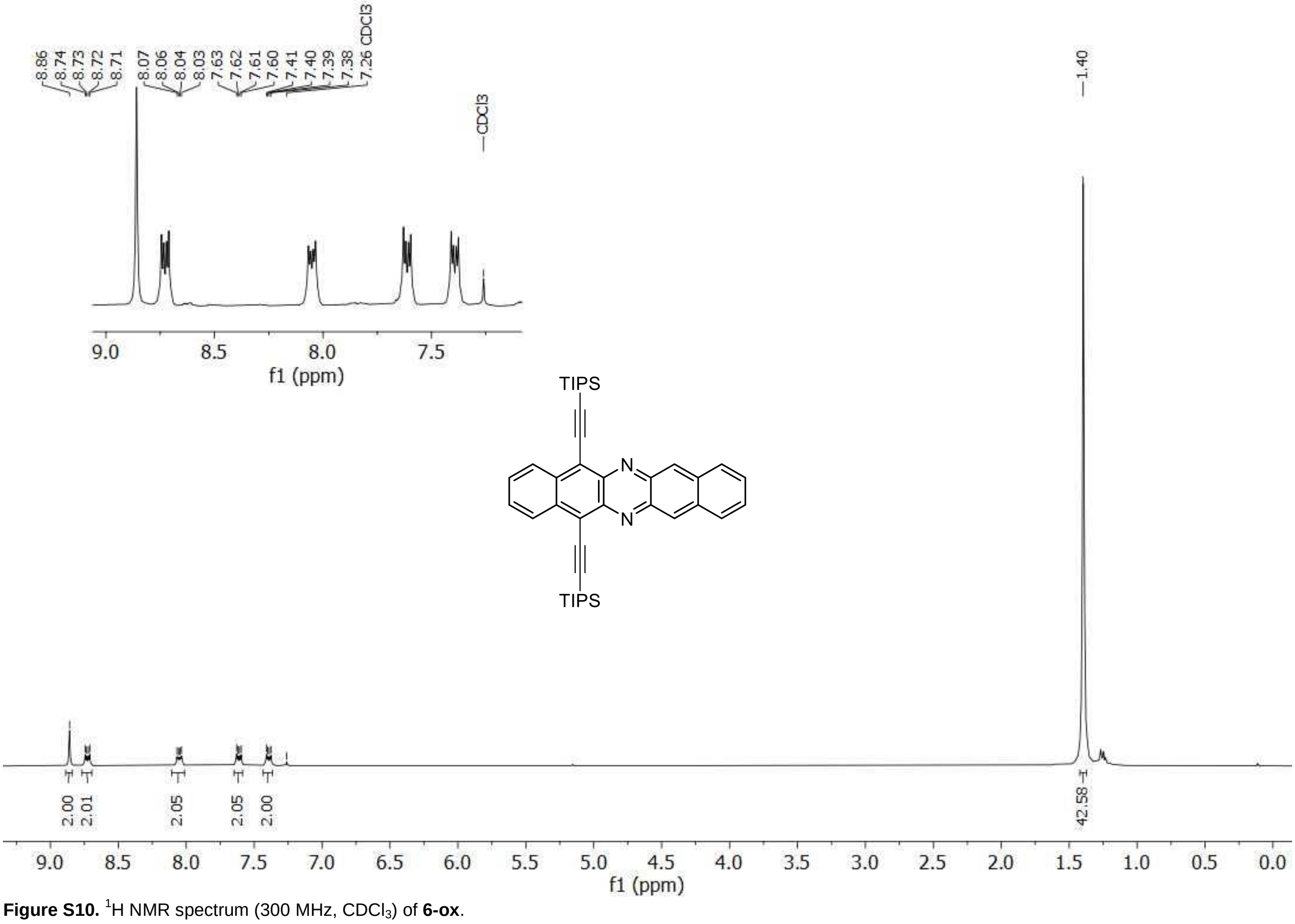

Figure S10. ^1H NMR spectrum (300 MHz, $CDCl_3$) of **6-ox**.

3 UV-Vis Spectra

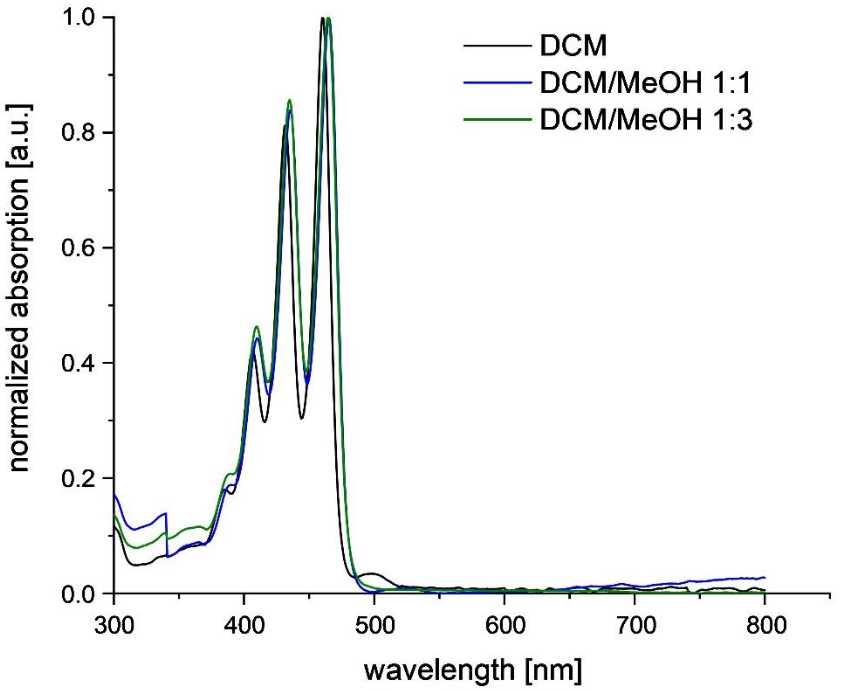

Figure S11. Normalized absorption spectra of **4** in DCM, DCM:MeOH 1:1 and DCM:MeOH 1:3.

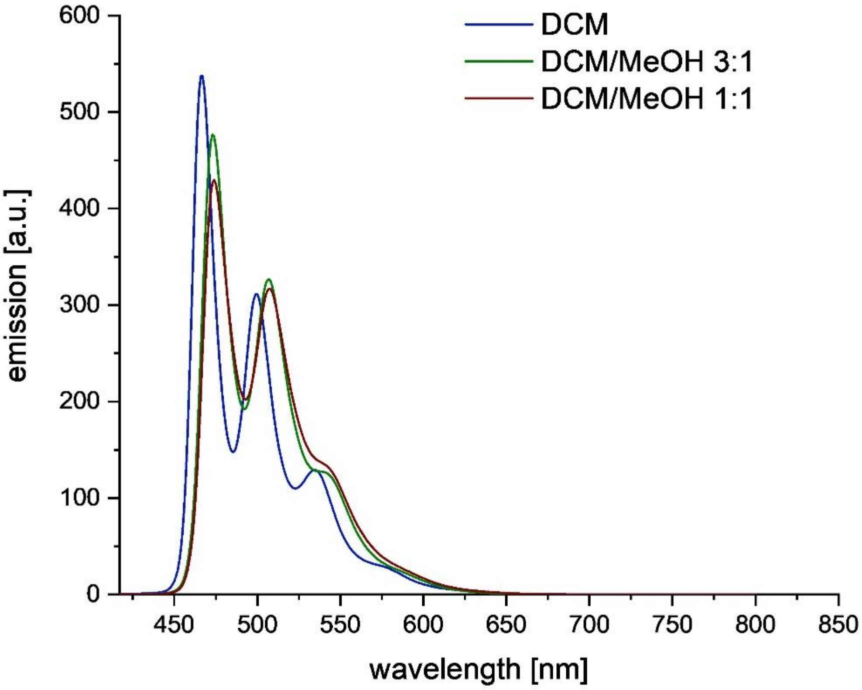

Figure S12. Non-normalized emission spectra of **4** in DCM, DCM:MeOH 3:1 and DCM:MeOH 1:1 (0.000015 mmol/l)

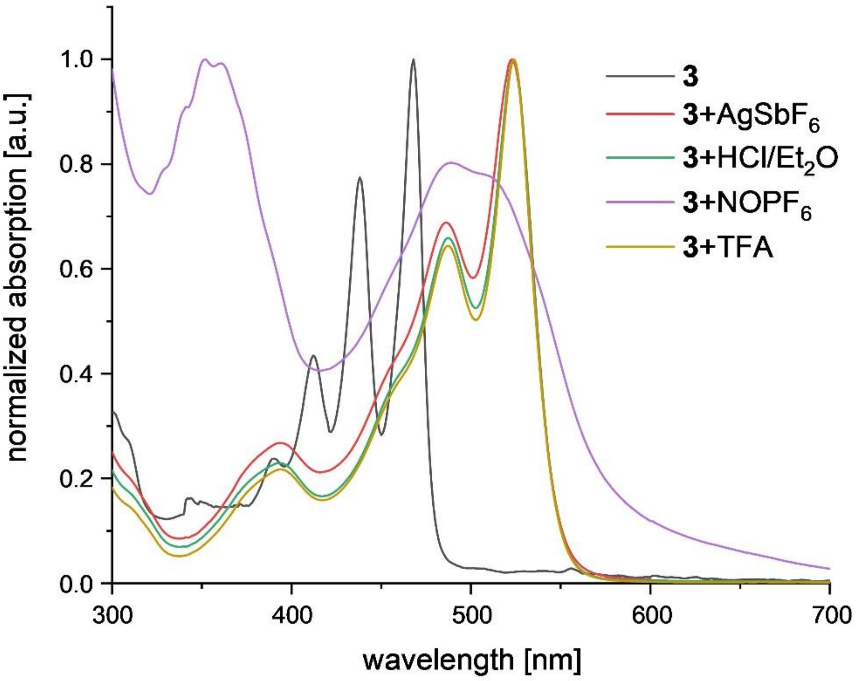

Figure S13. Normalized absorption spectra of **3** in DCM with different additives.

4 Crystallographic Data

Table S1. Crystal structure, crystal data and structure refinement of **1** (CCDC 2122131).

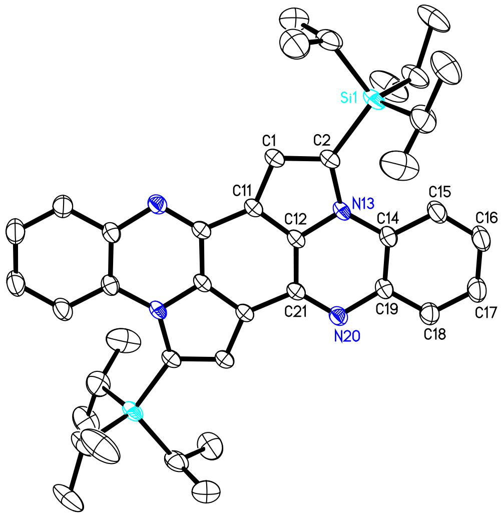

Identification code	tow25sq
Empirical formula	$C_{41.33}H_{54.67}Cl_{2.67}N_4Si_2$
Formula weight	758.27
Temperature	200(2) K
Wavelength	0.71073 Å
Crystal system	trigonal
Space group	R $\bar{3}$
Z	9
Unit cell dimensions	a = 21.5002(14) Å α = 90 deg.
	b = 21.5002(14) Å β = 90 deg.
	c = 23.1827(16) Å γ = 120 deg.
Volume	9280.7(14) $Å^3$
Density (calculated)	1.22 g/cm^3
Absorption coefficient	0.29 mm^{-1}
Crystal shape	polyhedron
Crystal size	0.310 x 0.190 x 0.160 mm^3
Crystal colour	yellow
Theta range for data collection	1.4 to 30.4 deg.
Index ranges	$-30 \leq h \leq 30$, $-27 \leq k \leq 30$, $-33 \leq l \leq 32$
Reflections collected	51126
Independent reflections	6227 (R(int) = 0.0548)
Observed reflections	4486 ($I > 2\sigma(I)$)
Absorption correction	Semi-empirical from equivalents
Max. and min. transmission	0.99 and 0.91
Refinement method	Full-matrix least-squares on F^2
Data/restraints/parameters	6227 / 189 / 208
Goodness-of-fit on F^2	1.03
Final R indices (I>2sigma(I))	R1 = 0.051, wR2 = 0.135
Largest diff. peak and hole	0.35 and -0.21 $eÅ^{-3}$

Table S2. Crystal structure, crystal data and structure refinement of **2** (CCDC 2122132).

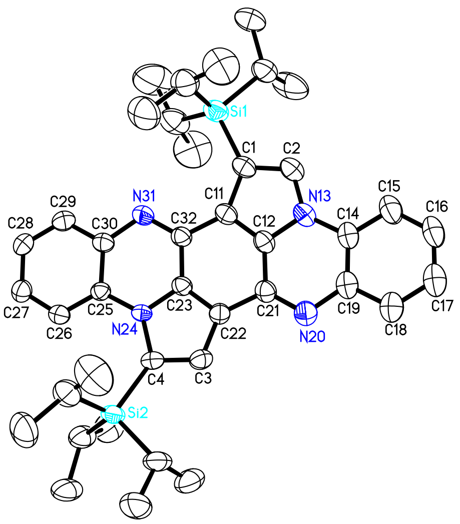

Identification code	tow26
Empirical formula	$C_{40}H_{52}N_4Si_2$
Formula weight	645.03
Temperature	200(2) K
Wavelength	0.71073 Å
Crystal system	monoclinic
Space group	$P2_1/n$
Z	4
Unit cell dimensions	a = 15.7737(15) Å α = 90 deg.
	b = 13.2662(13) Å β =94.824(3) deg.
	c = 17.4482(18) Å γ = 90 deg.
Volume	3638.2(6) $Å^3$
Density (calculated)	1.18 g/cm^3
Absorption coefficient	0.13 mm^{-1}
Crystal shape	polyhedron
Crystal size	0.220 x 0.150 x 0.080 mm^3
Crystal colour	orange
Theta range for data collection	1.7 to 20.5 deg.
Index ranges	$-15\leq h\leq 15$, $-13\leq k\leq 13$, $-16\leq l\leq 17$
Reflections collected	27169
Independent reflections	3623 (R(int) = 0.0852)
Observed reflections	2528 ($I > 2\sigma(I)$)
Absorption correction	Semi-empirical from equivalents
Max. and min. transmission	0.96 and 0.88
Refinement method	Full-matrix least-squares on F^2
Data/restraints/parameters	3623 / 408 / 415
Goodness-of-fit on F^2	1.04
Final R indices (I>2sigma(I))	R1 = 0.066, wR2 = 0.164
Largest diff. peak and hole	0.45 and -0.33 $eÅ^{-3}$

Table S3. Crystal structure, crystal data and structure refinement of **3** (CCDC 2122133)

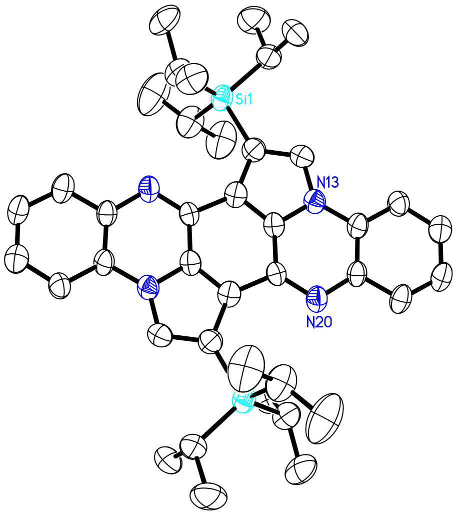

Identification code	mai23	
Empirical formula	$C_{40}H_{52}N_4Si_2$	
Formula weight	645.03	
Temperature	200(2) K	
Wavelength	1.54178 Å	
Crystal system	triclinic	
Space group	$P\bar{1}$	
Z	2	
Unit cell dimensions	a =8.3059(8) Å	α =84.647(8) deg.
	b =8.4109(8) Å	β =81.281(8) deg.
	c = 26.588(3) Å	γ =88.602(8) deg.
Volume	1827.9(3) $Å^3$	
Density (calculated)	1.17 g/cm^3	
Absorption coefficient	1.12 mm^{-1}	
Crystal shape	plate	
Crystal size	0.060 x 0.058 x 0.015 mm^3	
Crystal colour	yellow	
Theta range for data collection	3.4 to 54.2 deg.	
Index ranges	$-8 \le h \le 8$, $-5 \le k \le 8$, $-27 \le l \le 27$	
Reflections collected	12358	
Independent reflections	4414 (R(int) = 0.1665)	
Observed reflections	2389 ($I > 2\sigma(I)$)	
Absorption correction	Semi-empirical from equivalents	
Max. and min. transmission	1.60 and 0.57	
Refinement method	Full-matrix least-squares on F^2	
Data/restraints/parameters	4414 / 585 / 427	
Goodness-of-fit on F^2	1.12	
Final R indices (I>2sigma(I))	R1 = 0.121, wR2 = 0.220	
Largest diff. peak and hole	0.34 and -0.31 $eÅ^{-3}$	

Table S4. Crystal structure, crystal data and structure refinement of **4** (CCDC 2122134).

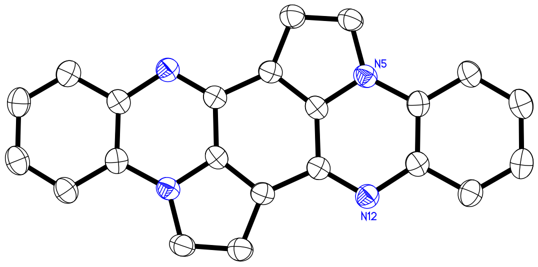

Identification code	hek15	
Empirical formula	$C_{24}H_{20}N_4O_2$	
Formula weight	396.44	
Temperature	200(2) K	
Wavelength	1.54178 Å	
Crystal system	Monoclinic	
Space group	$P2_1/n$	
Z	2	
Unit cell dimensions	a =9.1899(6) Å	α = 90 deg.
	b =4.7270(2) Å	β =98.365(6) deg.
	c = 21.9866(16) Å	γ = 90 deg.
Volume	944.95(10) $Å^3$	
Density (calculated)	1.39 g/cm^3	
Absorption coefficient	0.74 mm^{-1}	
Crystal shape	needle	
Crystal size	0.436 x 0.020 x 0.018 mm^3	
Crystal colour	brown	
Theta range for data collection	4.1 to 69.2 deg.	
Index ranges	$-10 \leq h \leq 11$, $-2 \leq k \leq 5$, $-26 \leq l \leq 24$	
Reflections collected	6680	
Independent reflections	1715 (R(int) = 0.0437)	
Observed reflections	1277 (I > 2\s(I))	
Absorption correction	Semi-empirical from equivalents	
Max. and min. transmission	1.00 and 0.56	
Refinement method	Full-matrix least-squares on F^2	
Data/restraints/parameters	1715 / 0 / 176	
Goodness-of-fit on F^2	1.01	
Final R indices ($I>2\sigma(I)$)	R1 = 0.040, wR2 = 0.097	
Largest diff. peak and hole	0.15 and -0.18 $eÅ^{-3}$	

5 Computational Details

5.1 Mechanism of the Cycloisomerization

Quantum chemical calculations (geometry optimization as well as frequency analysis) were conducted using wB97X-D3 functional in conjunction with the pc-1 basis set as implemented in the Q-Chem 5.2.2 software package.[6] Investigations of the reaction mechanism involving the explicit catalyst was done using the def2-ECP on the PBE-D3/def2-SV(P) level of theory as implemented in the ORCA 4.10 software package.[7] A def2-TZVPP basis set was used on gold, the alkyne moiety as well as the adjacent proton for better description of the important interactions within the crucial parts of the system regarding the possible mechanisms.

5.1.1 Cyclization Mode Through the Pyrazine-Substructure

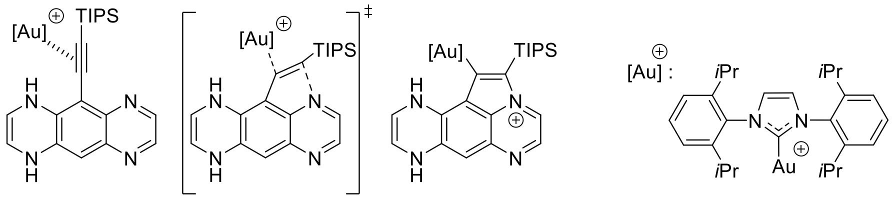

	C-a	C-b	C0
ΔG [kcal/mol]	0	19.2	-4.6

Figure S14. Cyclization via gold vinyl species.

For an alternative route through a gold vinylidene species, we were not able to find a stable minimum at this level of computation. The energy for the vinylidene complex **C-d** is about 20 kcal/mol higher than for the Au-alkyne complex **C-c**. The vinylidene pathway was therefore deemed unfavorable.

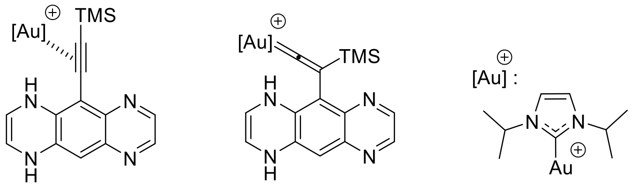

	C-c	C-d
ΔE [kcal/mol]:	0	~20

Figure S15. Cyclization via gold vinylidene species.

5.1.2 Calculated Thermodynamic Data

Molecule	H [kcal/mol]	S [kcal/mol*K]	G [kcal/mol]
**TIPS-TAP-H_2**	-1180132.85249553	140.95546414	-1180199.90365503
**1**	-1180195.18596277	125.52439032	-1180254.89670877
**2**	-1180200.93341253	124.36851875	-1180260.09432153
**3**	-1180206.69289568	126.62504111	-1180266.92721068
**5**	-1180191.48594875	129.39737587	-1180253.03903675
**6**	-1160005.67980367	142.50101881	-1160073.46616867
**7**	-1180148.90230180	141.08410349	-1180216.01465380
**8**	-1160057.36858798	129.95272134	-1160119.18584848
**9**	-1180205.35959835	123.65378600	-1180264.18051585
**B1**	-1180165.80921804	129.42937883	-1180227.37752954
**B2**	-1180160.70667857	128.02564120	-1180221.60724557
**C-a**	-1641401.92345661	93.40092087	-1641495.32437749
**C-b**	-1641384.46000228	91.62378228	-1641476.08378456
**C0**	-1641407.68007663	90.68874902	-1641498.36882566
**C1**	-1641407.54768478	90.61328479	-1641498.16096330
**C2**	-1641404.73006897	90.06117100	-1641494.79123997
**C3**	-1641423.28871667	90.91326547	-1641514.20197587
**C5**	-1641422.88721132	90.30409855	-1641513.19130987
**C6**	-1641410.29527687	90.00974036	-1641500.30501722
**C7**	-1641416.59106211	90.50948853	-1641507.10055064
**D1**	-1056684.90630517	59.41653053	-1056744.32282942
**D2**	-1056647.27651689	59.24816986	-1056706.52468676
**D3**	-1056687.51333524	60.47476798	-1056747.98810322

5.1.3 Coordinates of the Optimized Geometries

1:

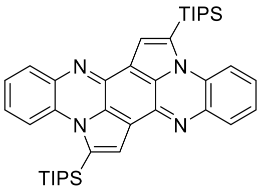

C	4.4374362214	-4.0242838219	-0.0035770765
C	5.2599649849	-2.8956105677	-0.001230708
C	4.708045932	-1.6281199198	0.0008231063
C	3.3239686251	-1.4531992622	0.0005793898
C	2.4786018711	-2.5907300142	-0.0021115501
C	3.0679658474	-3.8644855281	-0.0040270401
N	2.7017909479	-0.1913273357	0.0025379681
C	1.3352365487	-0.1621660561	0.0001590934
C	0.553064007	-1.3672567431	-0.0023272689
N	1.0912088528	-2.5440700094	-0.003127204
C	0.8933899356	1.1470214154	0.0021172173
C	-0.5401865036	1.4073035235	0.000810169
C	-1.3208507068	0.2001612073	-0.0019397597
C	-0.877167977	-1.1078592486	-0.0028874294
N	-1.0873203445	2.5806115972	0.0030138078
C	-2.4764128945	2.6192260299	0.0006103225
C	-3.3163785827	1.4770491204	-0.0025804615
N	-2.6897000722	0.2194404878	-0.0014799265
C	-3.0795071038	3.8867444268	0.0005591196
C	-4.4511077366	4.0333956651	-0.0034776827
C	-5.2654426543	2.8986497019	-0.0082365505
C	-4.7024451513	1.6365809702	-0.0080335882
C	2.056571421	1.9428654603	0.0060598535
C	3.1786740321	1.1236196317	0.0067373878
Si	4.9263536672	1.828261657	0.0149504287
C	5.8590505766	1.3639719268	-1.547157293
C	5.8480994757	1.3506547893	1.5794614772
C	4.7202079597	3.6901391814	0.0216612124
C	-5.8553034926	-1.3811511178	1.5698136221
C	-4.6912967149	-3.699404103	0.0246164146
C	-3.1645390877	-1.098792169	-0.0008192422
Si	-4.908935757	-1.8370213765	0.0130495787
C	-2.037346375	-1.9100736062	-0.0021390693
C	-5.8791713999	-1.40685163	-1.5365770991
H	4.8726452038	-5.0213814558	-0.0051743283
H	6.3424140359	-3.004544569	-0.0010850643
H	5.3636924379	-0.7690668185	0.0023596954
H	2.3907163627	-4.7160123274	-0.0060205883
H	-2.4128789895	4.7460702871	0.0034635523
H	-4.8960214898	5.0261054479	-0.0035572314

H	-6.3490211085	2.998179928	-0.0125840953
H	-5.3489669609	0.7689136191	-0.0130778523
H	2.0681851134	3.02612956	0.0086683491
H	6.8378606335	1.861704156	-1.566864963
H	5.2962942735	1.7088424846	-2.4243747625
H	6.0322412915	0.2903856203	-1.6803875833
H	6.8285650101	1.844669048	1.6084352232
H	5.2812271129	1.6918355716	2.455481779
H	6.0164432221	0.2755371595	1.7065478204
H	4.1815661212	4.0455479072	0.9090037107
H	5.7089412531	4.1684620572	0.0275445809
H	4.1888357077	4.0526413851	-0.8672025457
H	-6.8261589112	-1.8952511586	1.5748321763
H	-6.0505257943	-0.3107635568	1.6984175157
H	-5.3019771994	-1.717993024	2.4558821969
H	-4.1661288301	-4.0706194738	-0.8642715438
H	-5.6835112868	-4.1716549733	0.0328286067
H	-4.1565084038	-4.0588241078	0.9125897725
H	-2.0464514922	-2.9940875232	-0.001637836
H	-6.851605865	-1.9174918662	-1.5149086097
H	-5.3426463769	-1.762419241	-2.4256153154
H	-6.0735877299	-0.3385240272	-1.6826422919

2:

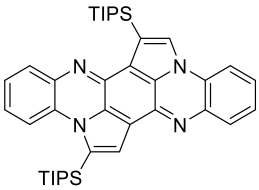

C	5.0471523722	-4.2670752449	0.0216120839
C	5.8382245463	-3.1139833613	0.0184818039
C	5.2495162862	-1.8625131128	0.0135735388
C	3.8588988148	-1.7529335602	0.0117931271
C	3.0399467061	-2.9067252834	0.0149828721
C	3.6711182638	-4.1596121843	0.0198741542
N	3.1992999648	-0.5176475426	0.0069563909
C	1.8365117452	-0.5000070255	0.0066553437
C	1.0810600069	-1.7211820891	0.0099181451
N	1.6487869846	-2.8860302247	0.0137268329
C	1.3986236512	0.8099128014	0.001525082
C	-0.0393523663	1.0425633225	0.0003019822
C	-0.8075343504	-0.1740753309	0.0042520996
C	-0.3537820325	-1.4801778818	0.0077710628
N	-0.6066576704	2.2071368669	-0.0050045092
C	-1.9938283831	2.2352267401	-0.0038996344
C	-2.8199294671	1.0842748563	0.0008889368
N	-2.1776884983	-0.1660637124	0.0018043963
C	-2.6069516571	3.4977883885	-0.0065104991
C	-3.9796958904	3.6313173279	-0.0031343768
C	-4.782281381	2.4883860746	0.0038556378

C	-4.2073819754	1.231430153	0.0061917293
C	2.5545229323	1.6462847167	-0.001849992
C	3.6400697793	0.7879725477	0.0017780706
Si	2.6014332319	3.5243297901	-0.0112924852
C	1.7469166072	4.1395298797	-1.5578031206
C	4.4134072046	4.0178465995	-0.0142727135
C	1.7465666059	4.1551294623	1.5287190946
C	-5.3324896782	-1.7810039061	-1.5693395154
C	-4.1604985888	-4.0951309239	-0.0255602749
C	-2.6418872671	-1.4877404538	0.0021448504
Si	-4.3837907525	-2.2338460829	-0.0129007923
C	-1.5086548454	-2.2906610735	0.0062505953
C	-5.3536913159	-1.8075616417	1.5382619
H	5.515480633	-5.2491231735	0.0254274352
H	6.9229367575	-3.1966096978	0.01994048
H	5.8647999779	-0.966372193	0.0112443676
H	3.027855489	-5.0372497371	0.0222322819
H	-1.9501138533	4.364861963	-0.0107870031
H	-4.4341514254	4.6196630955	-0.0051215411
H	-5.8667325279	2.5773391676	0.0081174496
H	-4.8469969426	0.359046024	0.0133761558
H	4.6993171825	1.0116393922	0.0008869844
H	1.7735916004	5.2350713169	-1.6285255051
H	2.2156950631	3.7311118781	-2.4621789172
H	0.6974678715	3.8200501875	-1.5462323654
H	4.9349052246	3.6415654746	0.8758597694
H	4.9338386103	3.6326800992	-0.9012440657
H	4.5253488622	5.1100706284	-0.0199142759
H	1.7740894592	5.2512482115	1.5890734254
H	0.6969019669	3.8362427018	1.5192879137
H	2.2143403896	3.7547437422	2.4371865354
H	-6.3056727078	-2.2906942342	-1.5689025742
H	-5.5236440118	-0.7106074175	-1.7036555443
H	-4.7834271303	-2.1250286975	-2.4552736807
H	-3.6370056937	-4.4660445905	0.8643892366
H	-3.622817335	-4.4523263518	-0.9126526927
H	-5.1517596637	-4.5693678158	-0.036892614
H	-1.5108112869	-3.3747418554	0.0065776789
H	-4.8163483119	-2.1660603132	2.425626962
H	-6.325974311	-2.318526051	1.5160503268
H	-5.5486947804	-0.7399759304	1.6886125643

3:

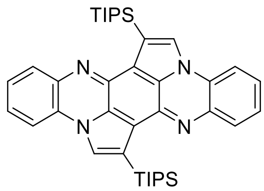

C	4.4374362214	-4.0242838219	-0.0035770765
C	5.2599649849	-2.8956105677	-0.001230708

C	4.708045932	-1.6281199198	0.0008231063
C	3.3239686251	-1.4531992622	0.0005793898
C	2.4786018711	-2.5907300142	-0.0021115501
C	3.0679658474	-3.8644855281	-0.0040270401
N	2.7017909479	-0.1913273357	0.0025379681
C	1.3352365487	-0.1621660561	0.0001590934
C	0.553064007	-1.3672567431	-0.0023272689
N	1.0912088528	-2.5440700094	-0.003127204
C	0.8933899356	1.1470214154	0.0021172173
C	-0.5401865036	1.4073035235	0.000810169
C	-1.3208507068	0.2001612073	-0.0019397597
C	-0.877167977	-1.1078592486	-0.0028874294
N	-1.0873203445	2.5806115972	0.0030138078
C	-2.4764128945	2.6192260299	0.0006103225
C	-3.3163785827	1.4770491204	-0.0025804615
N	-2.6897000722	0.2194404878	-0.0014799265
C	-3.0795071038	3.8867444268	0.0005591196
C	-4.4511077366	4.0333956651	-0.0034776827
C	-5.2654426543	2.8986497019	-0.0082365505
C	-4.7024451513	1.6365809702	-0.0080335882
C	2.056571421	1.9428654603	0.0060598535
C	3.1786740321	1.1236196317	0.0067373878
Si	4.9263536672	1.828261657	0.0149504287
C	5.8590505766	1.3639719268	-1.547157293
C	5.8480994757	1.3506547893	1.5794614772
C	4.7202079597	3.6901391814	0.0216612124
C	-5.8553034926	-1.3811511178	1.5698136221
C	-4.6912967149	-3.699404103	0.0246164146
C	-3.1645390877	-1.098792169	-0.0008192422
Si	-4.908935757	-1.8370213765	0.0130495787
C	-2.037346375	-1.9100736062	-0.0021390693
C	-5.8791713999	-1.40685163	-1.5365770991
H	4.8726452038	-5.0213814558	-0.0051743283
H	6.3424140359	-3.004544569	-0.0010850643
H	5.3636924379	-0.7690668185	0.0023596954
H	2.3907163627	-4.7160123274	-0.0060205883
H	-2.4128789895	4.7460702871	0.0034635523
H	-4.8960214898	5.0261054479	-0.0035572314
H	-6.3490211085	2.998179928	-0.0125840953
H	-5.3489669609	0.7689136191	-0.0130778523
H	2.0681851134	3.02612956	0.0086683491
H	6.8378606335	1.861704156	-1.566864963
H	5.2962942735	1.7088424846	-2.4243747625
H	6.0322412915	0.2903856203	-1.6803875833
H	6.8285650101	1.844669048	1.6084352232
H	5.2812271129	1.6918355716	2.455481779
H	6.0164432221	0.2755371595	1.7065478204
H	4.1815661212	4.0455479072	0.9090037107
H	5.7089412531	4.1684620572	0.0275445809
H	4.1888357077	4.0526413851	-0.8672025457

H	-6.8261589112	-1.8952511586	1.5748321763
H	-6.0505257943	-0.3107635568	1.6984175157
H	-5.3019771994	-1.717993024	2.4558821969
H	-4.1661288301	-4.0706194738	-0.8642715438
H	-5.6835112868	-4.1716549733	0.0328286067
H	-4.1565084038	-4.0588241078	0.9125897725
H	-2.0464514922	-2.9940875232	-0.001637836
H	-6.851605865	-1.9174918662	-1.5149086097
H	-5.3426463769	-1.762419241	-2.4256153154
H	-6.0735877299	-0.3385240272	-1.6826422919

5:

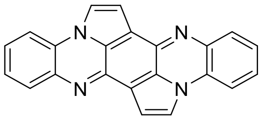

C	4.8487563464	-0.6768495365	-0.0441488294
C	4.8486102068	0.7096007668	-0.0402016998
C	3.6439969648	1.3963348461	-0.0311028571
C	2.4213367894	0.7355191383	-0.0252601118
C	2.4226924344	-0.6981296861	-0.028043957
C	3.6436213352	-1.3620882199	-0.0381842552
N	1.1960677271	1.4550002768	-0.0168865405
C	0.0671449882	0.7111489493	-0.0113256905
C	0.0713461615	-0.6723787802	-0.013410873
N	1.20018028	-1.4188351894	-0.020827879
C	-1.0755407971	1.4605204793	-0.0033362037
C	-2.3471088576	0.7481781133	0.0030343688
C	-2.340073738	-0.725359323	0.0008481371
C	-1.0669794621	-1.4261383929	-0.0075365455
N	-3.4795029973	1.4145259926	0.0104153911
C	-4.6321599527	0.7019335322	0.0156332006
C	-4.6230303038	-0.7160627701	0.0134635416
N	-3.4610104457	-1.4105877785	0.0061463321
C	-5.8760053325	1.3870946091	0.0231889955
C	-7.0480626006	0.6826519133	0.0280914845
C	-7.0378712637	-0.7363901506	0.0258595751
C	-5.8551467405	-1.4220286389	0.018804281
C	-0.5993130762	2.8028559722	-0.0041930258
C	0.7893176827	2.8098491367	-0.0125747486
Si	1.7704888901	4.4135871002	-0.0185839629
C	0.5049062669	5.7943221582	-0.0104860058
C	2.7953829901	4.5701287578	-1.5834979569
C	2.8151699432	4.57034626	1.5331723763
C	2.7993121598	-4.5912266937	1.5243241175
C	0.7955463856	-2.776650676	-0.019314807
C	-0.592546426	-2.7677463685	-0.0113690972
Si	1.7502426131	-4.4043329952	-0.0210022249
C	2.7792380248	-4.6024504072	-1.5783580569
C	0.4717124892	-5.7758860992	-0.0089461271
H	5.7821795395	-1.2345364897	-0.0516431104

H	5.7824842691	1.2663647144	-0.044301497
H	3.6524473515	2.4784875251	-0.0282845521
H	3.6480066445	-2.4456500065	-0.0417094885
H	-5.8547012245	2.4749364857	0.0246853536
H	-8.0002368849	1.2102285284	0.0336020331
H	-7.9819243248	-1.2785251068	0.0296945445
H	-5.8152431515	-2.5095490646	0.0168887811
H	-1.2235485211	3.6884775753	0.0005571484
H	1.0181803403	6.7653675738	-0.0127631044
H	-0.1336070798	5.7647831661	0.8812996688
H	-0.1436403742	5.7659138165	-0.8950406919
H	2.1353876889	4.5383923622	-2.4602063423
H	3.3243631944	5.5323700897	-1.6025226355
H	3.543633893	3.7810340936	-1.7195464617
H	3.3457992631	5.5317855306	1.5447296982
H	3.5638228236	3.780253839	1.6610149642
H	2.1659246919	4.5406049776	2.4179453399
H	3.3157382678	-5.5606619735	1.5156199211
H	2.1593535936	-4.5676095279	2.4159097958
H	3.5617695048	-3.8149712722	1.6554594967
H	-1.2174204488	-3.6535391542	-0.0082382792
H	3.2918482991	-5.573914355	-1.5714630096
H	3.5433064699	-3.8299212689	-1.7221440617
H	2.1287406424	-4.580003849	-2.4623065788
H	0.9880402742	-6.745850627	-0.0088880605
H	-0.176670558	-5.7553654277	-0.8938600444
H	-0.1668665809	-5.749726503	0.8828743572

6:

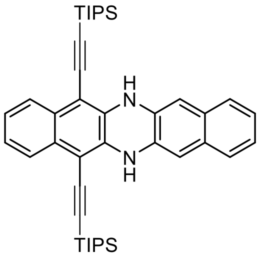

C	1.3449363899	-4.2071268351	0.3631962145
C	-0.0587617487	-4.4080214569	0.3997957852
C	-0.9174121528	-3.2773578423	0.3277936503
C	-0.4126006334	-2.0121170151	0.2267795166
C	1.0015987649	-1.807636876	0.195369432
C	1.8470853715	-2.8798969593	0.2597163819
N	-1.2303079453	-0.8917495928	0.1530639568
C	-0.7698156449	0.3990456307	0.0138536476
C	0.6436525524	0.6056377966	-0.010787104
N	1.4598361788	-0.4994843276	0.1002723508
C	-1.631891348	1.4697224728	-0.0998426402
C	-1.1212858643	2.8085847498	-0.2359363007
C	0.2773956832	3.0143516149	-0.2576105828
C	1.1554755997	1.8797252089	-0.1428040807

C	-1.9816293296	3.9209539288	-0.3506596105
C	-1.4728747723	5.1906939861	-0.4832848303
C	-0.0821071487	5.3953922158	-0.5061004844
C	0.7755708329	4.3270536438	-0.3952409792
C	2.5753509261	2.0129232121	-0.1558812317
C	3.7915197342	1.9909563854	-0.1503212257
C	-3.0324936756	1.1938887678	-0.0852414467
C	-4.200180355	0.8523957623	-0.0702653272
Si	5.6328231034	1.8599130503	-0.1187665631
C	6.2718937403	2.7363484318	1.4041009761
C	6.3216374784	2.6303144732	-1.6764429777
C	6.0302751304	0.029247351	-0.047631309
Si	-5.9642627616	0.3026035961	-0.0695518383
C	-5.9536647919	-1.5703043714	-0.0163084476
C	-6.7787689319	0.9173462115	-1.6362502341
C	-6.81166278	1.0034621574	1.4427754554
H	-1.9971378858	-3.4251529083	0.3520829811
H	2.9244383087	-2.717781444	0.2312838826
H	5.8438600998	2.3058088888	2.3177962072
H	7.3648765912	2.6584821653	1.4760442887
H	6.0125565628	3.8022813388	1.3884652201
H	5.9109203181	2.1495161519	-2.5727675417
H	7.4148813917	2.5349200847	-1.7141958853
H	6.077043345	3.6983293225	-1.733894839
H	5.6063988018	-0.4337681632	0.852971344
H	5.6279429474	-0.4987050038	-0.9218335536
H	7.1147724084	-0.1420064907	-0.0272251088
H	-5.4593702823	-1.9422360561	0.8904219977
H	-6.9757180058	-1.9719951195	-0.0219210415
H	-5.4294565372	-1.9911031643	-0.8839185211
H	-6.7384181794	2.0117644437	-1.7023265696
H	-7.8347774386	0.618682597	-1.6759553198
H	-6.2826007075	0.5140181055	-2.5275703122
H	-6.3370635435	0.6473402391	2.3654218788
H	-6.7690770093	2.0997084693	1.4505827491
H	-7.8691920969	0.7092285331	1.4744957352
H	-2.233986438	-1.021273143	0.1650314248
H	2.4577935292	-0.3354764124	0.0702643506
H	-3.0575214258	3.7539688524	-0.3325784447
H	1.8537379063	4.4776617245	-0.4134728579
H	-2.1491550171	6.0387913995	-0.5718379538
H	0.3175371245	6.4021416989	-0.6114920057
C	-0.5632785825	-5.7270383266	0.5040420239
C	2.1988964435	-5.3352156988	0.4290787376
C	1.6833704235	-6.6027915734	0.5284045635
C	0.2871052747	-6.8012514689	0.5668881333
H	-0.1147444522	-7.8098702481	0.6456710483
H	-1.6421468045	-5.8779046868	0.533326703
H	3.2773657367	-5.1827243281	0.3991862314
H	2.3515963448	-7.4606326673	0.5777133962

7:

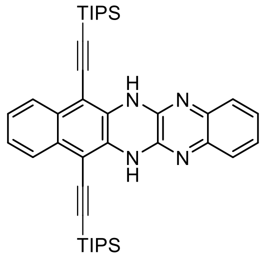

C	1.3591535113	-4.0364227003	0.4238360726
C	-0.0360275744	-4.2490809289	0.4553175576
N	-0.9159221273	-3.1939923719	0.3655697891
C	-0.4016563362	-2.015381613	0.2531621473
C	1.0302465922	-1.7957386214	0.2238668069
N	1.8787285037	-2.7659317853	0.3054198837
N	-1.2206512884	-0.9168798095	0.1566744094
C	-0.7663716666	0.3822482135	0.0087886554
C	0.641761679	0.5993751927	-0.0173754311
N	1.4728485497	-0.5012805958	0.1045636633
C	-1.639320215	1.4420392748	-0.1135902229
C	-1.1366570729	2.7796100953	-0.2642029143
C	0.2615502109	2.9956671205	-0.290199173
C	1.1483629188	1.873145191	-0.1641391078
C	-2.0060085347	3.8859032033	-0.39022983
C	-1.5057897724	5.1556218891	-0.5372583478
C	-0.1147683098	5.3705253138	-0.5641411064
C	0.75075654	4.3118943517	-0.4429852397
C	2.5685775435	2.0121663368	-0.1813629855
C	3.783861671	1.9781106407	-0.1769152609
C	-3.0392905553	1.1592578545	-0.0927101552
C	-4.2079957689	0.8246053049	-0.0684846278
Si	5.6252942193	1.7990694879	-0.1456066113
C	6.2717588121	2.5941590372	1.4179981577
C	6.3347032599	2.62495156	-1.6648718252
C	5.9734748954	-0.0406529557	-0.1541394168
Si	-5.97704192	0.2785891061	-0.0510111024
C	-5.9772269105	-1.5919662264	-0.0901522744
C	-6.8203156787	0.9817399906	-1.5645410397
C	-6.7756585062	0.915398958	1.5150123661
H	5.8377867045	2.1241013861	2.3091303317
H	7.3633750174	2.4963206334	1.4857941567
H	6.0285805821	3.6633519088	1.4548242815
H	5.9244447799	2.1865652057	-2.582835968
H	7.4263917507	2.5118963179	-1.6984373508
H	6.1094319408	3.6986240856	-1.6800678098
H	5.5225775014	-0.5388166539	0.7137193257
H	5.5728985178	-0.5199734955	-1.0562878594
H	7.0532397441	-0.2388075774	-0.1247906865
H	-5.4521012199	-2.0126705636	0.7766774301
H	-7.0026028406	-1.9849499133	-0.0781984514

H	-5.4867299927	-1.9744524265	-0.9940350787
H	-6.7705787503	2.0776627605	-1.5771666369
H	-7.8799570073	0.6948523166	-1.5925227899
H	-6.3508516013	0.6174597372	-2.4865928425
H	-6.280415771	0.5129245055	2.4072367435
H	-6.7247759506	2.0097856913	1.573039145
H	-7.8342083825	0.626825384	1.5613745312
H	-2.220681575	-1.0876586878	0.1726817637
H	2.4768074657	-0.3597789703	0.076960618
H	-3.0804307858	3.711184089	-0.3690141574
H	1.8278080275	4.4692673212	-0.4643869841
H	-2.1875009776	5.9982683376	-0.6347197863
H	0.2762268742	6.3792958652	-0.6811684736
C	-0.5381418875	-5.5575669341	0.5758558818
C	2.2282170406	-5.1392493518	0.5121885551
C	1.7186140235	-6.4131715666	0.6290279072
C	0.3281126574	-6.6238858921	0.6613970836
H	-0.064135133	-7.635139773	0.7531987614
H	-1.6180798779	-5.6901466013	0.597345468
H	3.299285381	-4.9498483197	0.4837009653
H	2.3942599411	-7.2638221409	0.6965555549

8:

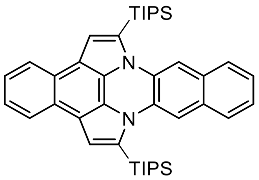

C	3.3827429106	-1.1312055194	-0.085154459
C	3.5609751734	0.2708266821	-0.1210186897
C	2.4143214977	1.1010374212	-0.1160581658
C	1.1415239754	0.6058394133	-0.066382666
C	0.9578916132	-0.8457310163	-0.0455179368
C	2.065283325	-1.64670586	-0.0555234061
N	0.0097422244	1.4613436156	-0.0546811131
C	-1.2058600994	0.8626532	-0.0986173146
C	-1.3760077287	-0.5044973708	-0.0892276497
N	-0.3499077889	-1.3914276269	-0.0357079104
C	-2.2525636947	1.7433861255	-0.094139308
C	-3.5998621702	1.1886280567	-0.1047747988
C	-3.7753764914	-0.2364119025	-0.0983047178
C	-2.6066054612	-1.0993696952	-0.0800770438
C	-4.7481206311	2.0002081526	-0.1132719114
C	-6.0165641856	1.456242123	-0.1155154592
C	-6.1849516254	0.0664591404	-0.1094103826
C	-5.0802850382	-0.7591698728	-0.1010994535
C	-1.6041221591	3.0181506231	-0.0411031191
C	-0.226125038	2.8510255262	-0.012727825
Si	0.9525584747	4.3046525196	0.1616196611
C	-0.127516058	5.82940912	0.3048162095

C	2.0353319939	4.5103952432	-1.3598007785
C	1.9697633469	4.1415506476	1.7303106637
C	0.8279500051	-4.6093645011	1.7028742721
C	-0.9254932793	-2.6806405561	0.0096491844
C	-2.3001966691	-2.4943955481	-0.0193181277
Si	-0.181062589	-4.4101381949	0.1325720451
C	0.8253540889	-4.8552371328	-1.389431798
C	-1.6191917391	-5.6123628763	0.2189998076
H	2.5605234778	2.1725058246	-0.1645543073
H	1.9377186151	-2.7232496345	-0.0514128474
H	-2.0945006885	3.9845858043	-0.0112739532
H	0.5011548004	6.723353447	0.4137004098
H	-0.7869847107	5.7836464595	1.180524318
H	-0.7522842174	5.9777920112	-0.5850278682
H	1.407674787	4.5516391402	-2.2595068894
H	2.5945044015	5.4539408511	-1.3031975539
H	2.7656291117	3.70850042	-1.517803268
H	2.6489418386	4.9967945858	1.8456594424
H	2.5755715152	3.2288764915	1.7740626606
H	1.3051632194	4.1258647315	2.6038869283
H	1.2427064887	-5.6246933628	1.763725656
H	0.1846821567	-4.4623962209	2.580094008
H	1.6633948089	-3.9059552644	1.7959969394
H	-3.0169025111	-3.3081401908	0.0099606929
H	1.2177302387	-5.8770953633	-1.2964806438
H	1.6771067173	-4.1935653317	-1.5851628885
H	0.1871373465	-4.8277487851	-2.2821461592
H	-1.2275838535	-6.6345963889	0.3152949892
H	-2.2394680623	-5.5894865948	-0.6860321146
H	-2.2702081366	-5.4326347806	1.0837142611
H	-5.2133195719	-1.8406830729	-0.0968433514
H	-4.6274893758	3.0825693192	-0.119071831
C	4.5218097574	-1.9758844913	-0.0925300802
C	4.875694184	0.7991483387	-0.1668176504
C	5.9598122804	-0.0376378874	-0.1735763671
C	5.7811607955	-1.4400730053	-0.1353414315
H	6.6517046815	-2.0932021918	-0.1408886129
H	4.3792818402	-3.0559298334	-0.0641656348
H	5.0104852926	1.8799160251	-0.1960969588
H	6.9657210953	0.3760851147	-0.2085056371
H	-6.8875442015	2.1092459095	-0.1224459009
H	-7.1849618251	-0.363796177	-0.1115169004

9:

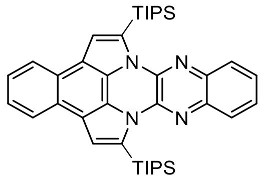

C	3.2284669137	-0.9965068159	-0.0066215991
C	3.3599817845	0.4068532679	-0.0058121815

N	2.2507372821	1.2105518841	-0.0044087861
C	1.0852691807	0.6593714948	-0.0037318854
C	0.9476526253	-0.8212892029	-0.004259039
N	1.9898609093	-1.5804945485	-0.0057953165
N	-0.064064178	1.4583193848	-0.0027742953
C	-1.2665506307	0.8234554735	-0.0042277099
C	-1.3913790033	-0.5443186449	-0.0045031119
N	-0.3282294732	-1.3947476247	-0.0032836819
C	-2.3304871895	1.6768046698	-0.0037005712
C	-3.6559855646	1.0732466487	-0.0043407082
C	-3.7851583366	-0.3607224083	-0.0046586403
C	-2.592138654	-1.1892298078	-0.0042893407
C	-4.8319353107	1.8461912998	-0.0044745664
C	-6.0805704018	1.2609189391	-0.0049700187
C	-6.2037619743	-0.1345854061	-0.005319943
C	-5.0743237154	-0.9242106378	-0.0051442934
C	-1.7059005866	2.976827544	-0.001599633
C	-0.3290827914	2.844376925	-0.0011267393
Si	0.9331353429	4.2398934912	0.0032879133
C	-0.0890310757	5.8139267938	0.007901879
C	1.9718416261	4.1728319623	-1.5514602182
C	1.9707677601	4.1630054544	1.5583345466
C	1.1336545128	-4.5060359523	1.5521747339
C	-0.8455341962	-2.7099005557	-0.00180955
C	-2.2221662595	-2.5813681318	-0.0026203496
Si	0.0951899186	-4.3461852492	0.0043744392
C	1.1291128334	-4.5227775731	-1.5445730613
C	-1.2192109059	-5.6885105313	0.0123838248
H	-2.2164379513	3.9338238243	-0.0003254758
H	0.5649000149	6.6962941768	0.0101572114
H	-0.7301004614	5.8822011306	0.8964036722
H	-0.7305737408	5.8872180705	-0.8798609987
H	1.3367669302	4.218429999	-2.4456228157
H	2.6745567401	5.0154473123	-1.598076024
H	2.5478362184	3.2417710259	-1.5944194915
H	2.6741825861	5.0047266714	1.6101859179
H	2.5460839405	3.2312942829	1.5963415931
H	1.3352331986	4.2040726367	2.4523903703
H	1.675170533	-5.461647443	1.5608943367
H	0.5069206468	-4.4717342383	2.4526100491
H	1.8689266743	-3.6965647023	1.6195656279
H	-2.9012319946	-3.4280953621	-0.0015098058
H	1.6677739761	-5.4800403624	-1.5462531553
H	1.8666400887	-3.7161733562	-1.6216430015
H	0.5000202824	-4.4946458783	-2.4435789836
H	-0.7334242944	-6.6741398815	0.016733801
H	-1.8624131587	-5.6513847578	-0.8763149673
H	-1.8612094018	-5.64262038	0.9015033796
H	-5.1722906219	-2.0093761567	-0.005456607
H	-4.7465254002	2.9318361702	-0.004250571

C	4.3797424376	-1.80818271	-0.008359691
C	4.6430536609	0.9872087778	-0.0067546745
C	5.7552055421	0.1801091229	-0.0084056962
C	5.6231351741	-1.2238583959	-0.0092157475
H	6.5150363225	-1.8471332764	-0.0105251762
H	4.248963333	-2.8886044001	-0.0090153791
H	4.7180293629	2.0726759661	-0.00617747
H	6.7472546332	0.6267682615	-0.0091252281
H	-6.9726213881	1.8847552552	-0.0051159657
H	-7.1895319885	-0.5963425895	-0.0057526681

B1:

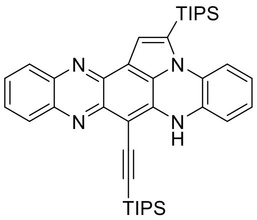

C	3.8931679978	-3.9053742433	0.0334663338
C	2.7537451331	-4.7000956647	0.022431044
C	1.4994425905	-4.1066006596	0.0183630938
C	1.3635745674	-2.718992832	0.022217385
C	2.5221601028	-1.9108676351	0.0276368676
C	3.7691146196	-2.5207704627	0.0373716544
N	0.0882352326	-2.1520035464	0.0234410023
C	-0.1093417597	-0.7988411637	0.0446591864
C	1.0662375885	-0.0038460568	0.0488497441
N	2.3434195264	-0.5029943	0.0292128552
C	-1.3306098099	-0.1660017646	0.0560537933
C	-1.3853220538	1.2926515814	0.0736533566
C	-0.1658894314	2.0835470561	0.0753161192
C	1.0906088872	1.3681391239	0.0600926447
N	-2.5685232227	1.873960848	0.0859084956
C	-2.5943853801	3.2274493708	0.1003720273
C	-1.4004941288	3.9950672032	0.1015109708
N	-0.1833478768	3.3982031873	0.0883935971
C	-3.8496626727	3.896046337	0.1145539052
C	-3.8984877666	5.2616390392	0.1293434101
C	-2.7015587183	6.0295092984	0.1304623284
C	-1.4813445189	5.4139104321	0.1168485183
C	2.4621094774	1.7294447624	0.0438634927
C	3.2362072328	0.5785780799	0.0231441085
C	-2.5157598008	-0.9581996419	0.0439883517
C	-3.4540171477	-1.7345895655	0.0275839028
Si	5.1236358705	0.681200374	-0.0522493817
C	5.5231775715	2.5103038423	-0.1004831759
C	5.790610029	-0.0905686382	-1.6276198742
C	5.9308583776	-0.0306274667	1.486249115
Si	-4.9634318154	-2.7927640524	-0.005506433
C	-4.4320446736	-4.5627430476	0.3072058522

C	-5.7604365392	-2.6377337087	-1.6907134888
C	-6.125499635	-2.2047692952	1.3361576419
H	4.882512375	-4.3548845546	0.040847204
H	2.8358210757	-5.7840715203	0.019419492
H	0.5992758558	-4.7190220421	0.0137276811
H	4.6605110107	-1.9088517626	0.0518066607
H	-4.7455182369	3.279750355	0.1133228228
H	5.1673631516	3.038665468	0.7928593819
H	6.6129712601	2.6428924763	-0.1453283187
H	5.0974539184	3.0071190253	-0.9811711994
H	5.6225267311	-1.1689579587	-1.7250298944
H	6.8735653278	0.0820806808	-1.6962798735
H	5.3264437217	0.3896260674	-2.4990947717
H	5.5390931639	0.476846557	2.377572141
H	5.7856024507	-1.10607293	1.637677417
H	7.0136487416	0.1534660457	1.4543383839
H	-3.9342730472	-4.6621971589	1.2804231813
H	-5.2935753889	-5.2437192239	0.3034734444
H	-3.7334075648	-4.9110965636	-0.4646467694
H	-6.0343851987	-1.5961565155	-1.9004426607
H	-6.6716696358	-3.2467954648	-1.7601520212
H	-5.0764338272	-2.9639825527	-2.4841111823
H	-5.6577293289	-2.2788893924	2.3257117718
H	-6.4017668574	-1.154335524	1.180810434
H	-7.0492163023	-2.7983556574	1.3525263023
H	-4.8593103706	5.771589148	0.1403381745
H	-2.7634453824	7.1155500398	0.1423132258
H	-0.5483705899	5.9726986984	0.1173374023
H	2.8399398989	2.7439700511	0.0423986068
H	-0.7272826277	-2.7539774517	0.0207404612

B2:

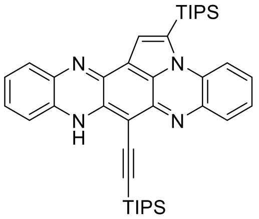

C	4.2296957005	3.701647021	0.1660552052
C	3.1367250187	4.5697356878	0.1333713654
C	1.8589057997	4.05226909	0.0922965551
C	1.6282426458	2.6681431773	0.0762657744
C	2.7471591857	1.7991033469	0.0938176675
C	4.0346447465	2.3332209416	0.1472564078
N	0.3046774647	2.2559560856	0.0522268868
C	0.095273461	0.9760006785	0.0610795477
C	1.1684897746	0.0346472065	0.0770591671
N	2.4763016021	0.4173527071	0.0700508299
C	-1.2322549996	0.3835914726	0.0405576886
C	-1.3741206821	-0.9909603385	0.0405157167

C	-0.2372344172	-1.9466550261	0.0536608192
C	1.0746754689	-1.3364808098	0.0653901903
N	-2.6067452042	-1.5655641047	0.0228986372
C	-2.8117408988	-2.9305462332	0.0163691854
C	-1.6752029862	-3.7552641536	0.0298477007
N	-0.3930694226	-3.2285897116	0.0482439949
C	-4.0944889497	-3.4804749932	-0.0049120175
C	-4.242033374	-4.8560635601	-0.0121583471
C	-3.1199732664	-5.6922892493	0.003251305
C	-1.8508126554	-5.1440996229	0.0241374177
C	2.4050038644	-1.8113955191	0.0431110076
C	3.2770310056	-0.7317991916	0.0410114189
C	-2.4005328689	1.1954447793	0.0125165952
C	-3.465323394	1.783296997	-0.0173201924
Si	5.1448385404	-0.9451956893	-0.102277525
C	5.4293319976	-2.7907760703	-0.2412270949
C	6.0442183907	-0.3467663074	1.4342804543
C	5.7816350985	-0.1363952173	-1.6721553602
Si	-5.0373043085	2.7474500275	-0.0671363612
C	-5.2202352443	3.5209037717	-1.7780583706
C	-4.9681696314	4.0731021902	1.2673833562
C	-6.4536639607	1.5406232073	0.2631189546
H	5.2435240292	4.0951826705	0.2074382556
H	3.2896540596	5.647250576	0.1470027341
H	0.9789510037	4.6923492014	0.0737582206
H	4.8952301811	1.6793478981	0.1837185497
H	-4.9646733476	-2.8253237654	-0.0157517167
H	4.9323908773	-3.221656562	-1.1190044437
H	6.5048633467	-2.9916296922	-0.3433582851
H	5.0806128267	-3.3317870119	0.647615214
H	5.9889423458	0.7323869317	1.6122671627
H	7.1078683022	-0.6157778753	1.3764416163
H	5.6276585466	-0.8402478002	2.3215835248
H	5.2708751144	-0.5703605921	-2.5415996053
H	5.6314766096	0.948345004	-1.7193723291
H	6.8568203533	-0.3276999143	-1.7924576302
H	-5.2612238932	2.7492931492	-2.567204793
H	-6.1430857069	4.1144116722	-1.8471652398
H	-4.3741567821	4.1935753556	-2.007955055
H	-4.849716894	3.62825356	2.27348583
H	-5.8885660324	4.6716961271	1.2712167821
H	-4.1185068815	4.7620791169	1.1045954155
H	-6.5044140724	0.7483178445	-0.5069171323
H	-6.3500308502	1.053753621	1.2504286257
H	-7.4201162361	2.0630448982	0.255048431
H	-5.2406292023	-5.2872998728	-0.0270979813
H	-3.2471538133	-6.7726243364	-0.0019403372
H	-0.956708823	-5.7630481062	0.0355203654
H	2.6942940414	-2.8555195407	0.0184840343
H	-3.4072784054	-0.9385377782	0.0139390436

C-a:

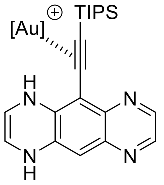

Au	4.490673000	-10.646394000	-5.138794000
C	2.552426000	-11.481025000	-6.220837000
C	2.480467000	-11.354012000	-4.970927000
C	6.416751000	-10.073091000	-5.177580000
N	7.370983000	-10.592782000	-6.006575000
C	8.606915000	-10.003861000	-5.769417000
H	9.499417000	-10.284500000	-6.338714000
C	8.416669000	-9.088834000	-4.764508000
H	9.107234000	-8.399394000	-4.267378000
N	7.071386000	-9.150466000	-4.416555000
C	7.075610000	-11.564956000	-7.031248000
C	6.814417000	-11.090170000	-8.339894000
C	6.458552000	-12.047063000	-9.308509000
H	6.233265000	-11.719850000	-10.335542000
C	6.383638000	-13.408070000	-8.983575000
H	6.105802000	-14.141012000	-9.757449000
C	6.661953000	-13.844842000	-7.682306000
H	6.603099000	-14.919664000	-7.447878000
C	7.013996000	-12.931279000	-6.670384000
C	6.863323000	-9.606210000	-8.685771000
H	7.395467000	-9.081795000	-7.862845000
C	7.306554000	-13.418097000	-5.255582000
H	7.593184000	-12.536102000	-4.643027000
C	6.443509000	-8.327720000	-3.410787000
C	6.567397000	-8.719077000	-2.054729000
C	5.969866000	-7.885482000	-1.092379000
H	6.042829000	-8.147478000	-0.025831000
C	5.279817000	-6.725206000	-1.471505000
H	4.821828000	-6.085551000	-0.699842000
C	5.162373000	-6.378278000	-2.822285000
H	4.608591000	-5.468348000	-3.103755000
C	5.744201000	-7.172063000	-3.830237000
C	7.255733000	-10.023082000	-1.662558000
H	7.991410000	-10.271460000	-2.459032000
C	5.595154000	-6.785618000	-5.297304000
H	6.180817000	-7.508030000	-5.906159000
C	7.645273000	-9.325157000	-9.979668000
H	7.741897000	-8.228659000	-10.135954000
H	7.128589000	-9.738299000	-10.873767000
H	8.667605000	-9.760585000	-9.944000000
C	5.441224000	-9.019538000	-8.748118000
H	4.884181000	-9.201087000	-7.803763000
H	4.858605000	-9.476656000	-9.578184000
H	5.479844000	-7.921497000	-8.922476000
C	8.492005000	-14.399670000	-5.229224000
H	8.730317000	-14.691971000	-4.182995000
H	9.402690000	-13.946468000	-5.677421000
H	8.265403000	-15.330708000	-5.794429000

C	6.051891000	-14.025995000	-4.602943000
H	6.265084000	-14.341375000	-3.557854000
H	5.696902000	-14.922063000	-5.160822000
H	5.226426000	-13.279254000	-4.572822000
C	8.033688000	-9.933048000	-0.341102000
H	8.614460000	-10.866478000	-0.176452000
H	7.356241000	-9.813754000	0.532809000
H	8.745510000	-9.079297000	-0.341795000
C	6.227760000	-11.172890000	-1.624183000
H	6.729848000	-12.142501000	-1.409140000
H	5.688875000	-11.267258000	-2.593526000
H	5.467256000	-10.994649000	-0.831719000
C	6.168526000	-5.385293000	-5.579960000
H	6.108972000	-5.157408000	-6.666960000
H	7.233373000	-5.311186000	-5.268880000
H	5.602740000	-4.592988000	-5.042020000
C	4.127238000	-6.900571000	-5.749994000
H	4.032399000	-6.648279000	-6.828965000
H	3.469922000	-6.209376000	-5.177265000
H	3.741219000	-7.935118000	-5.609864000
Si	1.495622000	-11.460783000	-3.370458000
C	1.415281000	-9.679715000	-2.686243000
H	0.707327000	-9.728055000	-1.823541000
C	0.838185000	-8.707719000	-3.731945000
H	-0.188621000	-8.989192000	-4.052133000
H	0.784819000	-7.674283000	-3.318746000
H	1.472225000	-8.670673000	-4.647151000
C	2.774816000	-9.192787000	-2.163217000
H	3.171183000	-9.827812000	-1.342071000
H	3.544737000	-9.179357000	-2.971618000
H	2.701586000	-8.152365000	-1.774440000
C	-0.247469000	-12.092742000	-3.858964000
H	-0.735953000	-11.195419000	-4.307400000
C	-0.252242000	-13.199012000	-4.926444000
H	0.252336000	-12.875244000	-5.863545000
H	0.250638000	-14.125054000	-4.568794000
H	-1.296947000	-13.477968000	-5.194984000
C	-1.059242000	-12.498267000	-2.614118000
H	-1.059767000	-11.709684000	-1.828858000
H	-2.121794000	-12.694849000	-2.885301000
H	-0.662880000	-13.430965000	-2.154350000
C	2.527289000	-12.620363000	-2.248403000
H	3.546883000	-12.168981000	-2.299133000
C	2.083508000	-12.610866000	-0.775026000
H	2.042763000	-11.583025000	-0.352580000
H	1.076999000	-13.064125000	-0.644055000
H	2.792033000	-13.199671000	-0.147745000
C	2.624833000	-14.053056000	-2.797869000
H	2.924896000	-14.069846000	-3.870895000
H	3.379397000	-14.648522000	-2.233836000
H	1.653279000	-14.588135000	-2.715042000
C	2.691944000	-12.295172000	-10.377497000
C	2.930171000	-13.311358000	-9.467714000
N	3.205635000	-14.626558000	-9.842229000
H	3.186240000	-14.852504000	-10.837567000
C	3.452582000	-15.635768000	-8.919844000

C	3.446003000	-15.358071000	-7.591174000
N	3.171481000	-14.052022000	-7.173696000
H	3.177938000	-13.821901000	-6.173516000
C	2.902666000	-13.033391000	-8.046252000
C	2.597096000	-11.728313000	-7.595372000
C	2.346692000	-10.658432000	-8.545316000
N	2.084163000	-9.418636000	-8.091483000
C	1.882364000	-8.474040000	-9.018668000
C	1.948665000	-8.757869000	-10.400710000
N	2.209719000	-9.982305000	-10.868415000
C	2.408871000	-10.955551000	-9.948196000
H	1.664462000	-7.452467000	-8.657738000
H	1.784881000	-7.958764000	-11.147024000
H	3.630247000	-16.106139000	-6.810949000
H	3.643373000	-16.641223000	-9.316705000
H	2.715915000	-12.484821000	-11.461090000

C-b:

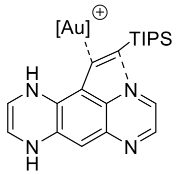

Au	-2.394805000	-2.753927000	-2.792616000
C	-1.540510000	-0.850905000	-2.840436000
C	-1.238718000	-0.646691000	-1.607367000
H	-0.682187000	2.803024000	-6.695820000
C	-0.886658000	2.024169000	-5.946730000
C	-1.353352000	0.774004000	-6.319103000
N	-1.579472000	0.425280000	-7.646920000
C	-1.998525000	-0.840920000	-8.039508000
H	-2.099890000	-1.024325000	-9.116698000
H	-2.596868000	-2.800240000	-7.330463000
C	-2.260065000	-1.782606000	-7.096941000
N	-2.105737000	-1.456822000	-5.750389000
C	-1.628732000	-0.245004000	-5.316192000
C	-1.386815000	0.036800000	-3.963220000
C	-0.874848000	1.328925000	-3.579324000
N	-0.615490000	1.562959000	-2.282550000
C	-0.158890000	2.778417000	-1.941573000
H	0.032334000	2.976170000	-0.875385000
H	0.424853000	4.768023000	-2.625093000
C	0.053350000	3.770297000	-2.920740000
N	-0.174125000	3.560017000	-4.221175000
C	-0.638091000	2.342034000	-4.574442000
C	-3.260917000	-4.504257000	-3.313130000
N	-2.632081000	-5.640904000	-3.748970000
C	-3.536135000	-6.491084000	-4.374045000
H	-3.227975000	-7.459810000	-4.781166000
C	-4.763384000	-5.880225000	-4.312011000
H	-5.751058000	-6.201264000	-4.658676000
N	-4.575386000	-4.670765000	-3.653985000
C	-1.286557000	-6.019735000	-3.373775000
C	-1.150700000	-6.905994000	-2.274429000
C	0.155707000	-7.272353000	-1.900753000
H	0.301941000	-7.961946000	-1.054106000

C	1.270786000	-6.772925000	-2.584345000
H	2.284948000	-7.070831000	-2.273184000
C	1.101594000	-5.898879000	-3.664965000
H	1.987817000	-5.520735000	-4.198007000
C	-0.181310000	-5.502050000	-4.088886000
C	-2.347607000	-7.483205000	-1.524557000
H	-3.264332000	-6.969946000	-1.883074000
C	-0.338979000	-4.592679000	-5.300305000
H	-1.392083000	-4.236539000	-5.314458000
C	-5.644267000	-3.796460000	-3.220530000
C	-6.166723000	-2.844913000	-4.125828000
C	-7.249912000	-2.061122000	-3.682900000
H	-7.691209000	-1.313785000	-4.360109000
C	-7.768062000	-2.210656000	-2.392089000
H	-8.615838000	-1.587015000	-2.065765000
C	-7.205115000	-3.139074000	-1.506333000
H	-7.612973000	-3.229001000	-0.488426000
C	-6.128131000	-3.956642000	-1.894741000
C	-5.603930000	-2.664430000	-5.529806000
H	-4.593590000	-3.132289000	-5.545337000
C	-5.535513000	-4.983218000	-0.933237000
H	-4.457167000	-5.089739000	-1.185258000
C	-2.514553000	-8.983523000	-1.834196000
H	-3.420241000	-9.386908000	-1.329966000
H	-1.637398000	-9.567099000	-1.476990000
H	-2.616223000	-9.169007000	-2.925925000
C	-2.268613000	-7.233458000	-0.009123000
H	-2.171933000	-6.149885000	0.214350000
H	-1.405520000	-7.758587000	0.455693000
H	-3.190775000	-7.607832000	0.487789000
C	-0.111663000	-5.380520000	-6.605158000
H	-0.271489000	-4.723541000	-7.489135000
H	-0.805434000	-6.246080000	-6.684171000
H	0.926991000	-5.775452000	-6.657422000
C	0.570426000	-3.354581000	-5.223088000
H	0.372950000	-2.676489000	-6.081195000
H	1.645917000	-3.632306000	-5.265064000
H	0.400246000	-2.784658000	-4.282135000
C	-6.468689000	-3.398647000	-6.573110000
H	-6.035467000	-3.285039000	-7.591625000
H	-7.500383000	-2.982983000	-6.592414000
H	-6.548987000	-4.485350000	-6.352825000
C	-5.433318000	-1.181002000	-5.899350000
H	-4.902223000	-1.085881000	-6.871254000
H	-4.845818000	-0.635937000	-5.128770000
H	-6.414398000	-0.669907000	-6.008852000
C	-6.197228000	-6.364743000	-1.117317000
H	-5.727070000	-7.112764000	-0.440892000
H	-6.099573000	-6.740692000	-2.157884000
H	-7.281436000	-6.315021000	-0.873121000
C	-5.603843000	-4.549922000	0.539118000
H	-5.001970000	-5.245620000	1.163188000
H	-6.643564000	-4.575545000	0.933202000
H	-5.202682000	-3.524363000	0.684941000
Si	-0.555239000	-0.897656000	0.118885000
C	-0.796726000	-2.753064000	0.586423000

H	-0.332802000	-2.777011000	1.603418000
C	-0.018608000	-3.741507000	-0.294186000
H	1.069132000	-3.519070000	-0.324697000
H	-0.131587000	-4.783062000	0.083785000
H	-0.392333000	-3.738829000	-1.346268000
C	-2.269862000	-3.155517000	0.733811000
H	-2.836201000	-2.481961000	1.412150000
H	-2.788691000	-3.147237000	-0.255307000
H	-2.364202000	-4.188030000	1.140991000
C	1.346813000	-0.594468000	0.043258000
H	1.732651000	-1.394627000	0.721615000
C	1.921142000	-0.849318000	-1.362683000
H	1.592376000	-1.819973000	-1.793676000
H	1.600883000	-0.052741000	-2.068581000
H	3.035109000	-0.849573000	-1.334184000
C	1.836448000	0.751391000	0.599475000
H	1.481135000	0.942049000	1.634559000
H	2.950334000	0.776687000	0.620903000
H	1.506513000	1.599331000	-0.037344000
C	-1.581548000	0.136527000	1.363126000
H	-2.613806000	-0.265958000	1.224585000
C	-1.159576000	-0.148300000	2.817148000
H	-1.223264000	-1.227307000	3.078447000
H	-0.116993000	0.184351000	3.019287000
H	-1.818543000	0.401248000	3.527799000
C	-1.646290000	1.643932000	1.075756000
H	-2.005415000	1.851649000	0.045691000
H	-2.343977000	2.143777000	1.786575000
H	-0.653688000	2.128908000	1.201136000
H	-2.303472000	-2.158984000	-5.016959000
H	-1.380424000	1.127317000	-8.360825000

C0:

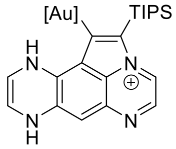

Au	-2.360660000	-2.778284000	-2.900350000
C	-1.427172000	-0.967918000	-2.794553000
C	-0.833104000	-0.239735000	-1.755125000
H	-0.296050000	2.698456000	-6.591990000
C	-0.573971000	1.910040000	-5.877311000
C	-1.203321000	0.745521000	-6.300887000
N	-1.506871000	0.504641000	-7.641989000
C	-2.089342000	-0.663109000	-8.095423000
H	-2.247940000	-0.755009000	-9.177954000
H	-2.903592000	-2.590047000	-7.502238000
C	-2.440903000	-1.639186000	-7.211436000
N	-2.203271000	-1.428120000	-5.859606000
C	-1.589019000	-0.322349000	-5.368513000
C	-1.286342000	-0.162927000	-4.000195000
C	-0.631884000	1.027133000	-3.647792000
N	-0.362826000	1.000624000	-2.307164000
C	0.274301000	2.094795000	-1.781788000
H	0.488844000	2.103456000	-0.707660000
H	1.121140000	4.024527000	-2.215221000
C	0.612699000	3.143956000	-2.644624000
N	0.375421000	3.174320000	-3.976436000
C	-0.253295000	2.094973000	-4.485996000
C	-3.290607000	-4.531914000	-3.376570000
N	-2.683729000	-5.679053000	-3.817933000
C	-3.612345000	-6.555923000	-4.367607000
H	-3.318390000	-7.531533000	-4.768658000
C	-4.838129000	-5.953050000	-4.250775000
H	-5.841307000	-6.289485000	-4.532030000
N	-4.621443000	-4.723537000	-3.635330000
C	-1.312009000	-6.023300000	-3.519757000
C	-1.082385000	-6.841400000	-2.384667000
C	0.253907000	-7.163200000	-2.081855000
H	0.472598000	-7.799088000	-1.209196000
C	1.308245000	-6.687475000	-2.870670000
H	2.347509000	-6.949132000	-2.614449000
C	1.047109000	-5.883721000	-3.987470000
H	1.887263000	-5.525465000	-4.602506000
C	-0.269781000	-5.532500000	-4.340363000
C	-2.217691000	-7.399353000	-1.531965000
H	-3.160748000	-6.903730000	-1.843976000
C	-0.539651000	-4.704706000	-5.590556000
H	-1.585911000	-4.333426000	-5.522484000
C	-5.676937000	-3.849970000	-3.173548000
C	-6.253841000	-2.929852000	-4.078196000
C	-7.328990000	-2.149288000	-3.610972000
H	-7.812200000	-1.428066000	-4.288099000
C	-7.789170000	-2.271277000	-2.295573000
H	-8.632097000	-1.651715000	-1.949396000
C	-7.174360000	-3.167820000	-1.411649000
H	-7.536998000	-3.236227000	-0.375212000
C	-6.102866000	-3.980699000	-1.824929000

C	-5.757282000	-2.783226000	-5.511283000
H	-4.744960000	-3.245958000	-5.559394000
C	-5.452090000	-4.971063000	-0.862829000
H	-4.378559000	-5.046668000	-1.144840000
C	-2.392049000	-8.910145000	-1.781595000
H	-3.256307000	-9.303682000	-1.202240000
H	-1.485869000	-9.474972000	-1.469189000
H	-2.568851000	-9.130688000	-2.857059000
C	-2.037596000	-7.101342000	-0.034365000
H	-1.932119000	-6.010819000	0.147817000
H	-1.142408000	-7.608564000	0.387787000
H	-2.922092000	-7.462973000	0.535328000
C	-0.450033000	-5.586434000	-6.851337000
H	-0.688155000	-4.993596000	-7.762920000
H	-1.158019000	-6.442441000	-6.799613000
H	0.574088000	-6.003419000	-6.973642000
C	0.381377000	-3.477869000	-5.691696000
H	0.110970000	-2.863936000	-6.578754000
H	1.446783000	-3.770676000	-5.813219000
H	0.300171000	-2.841248000	-4.782471000
C	-6.663802000	-3.550267000	-6.493671000
H	-6.281462000	-3.462860000	-7.535158000
H	-7.698702000	-3.142578000	-6.474836000
H	-6.723999000	-4.630698000	-6.239978000
C	-5.617691000	-1.308281000	-5.925157000
H	-5.156771000	-1.229535000	-6.933797000
H	-4.984372000	-0.743883000	-5.206180000
H	-6.605552000	-0.801774000	-5.980207000
C	-6.073609000	-6.376625000	-0.999934000
H	-5.560165000	-7.093578000	-0.321241000
H	-5.992181000	-6.773316000	-2.033813000
H	-7.151505000	-6.356147000	-0.725135000
C	-5.495875000	-4.514179000	0.603119000
H	-4.853244000	-5.177224000	1.221975000
H	-6.523355000	-4.569939000	1.025580000
H	-5.127220000	-3.473276000	0.720833000
Si	-0.520261000	-0.668088000	0.082664000
C	-0.814287000	-2.531068000	0.460735000
H	-0.424006000	-2.584781000	1.507671000
C	0.035751000	-3.495996000	-0.383441000
H	1.118000000	-3.245151000	-0.354599000
H	-0.068174000	-4.542872000	-0.017532000
H	-0.282960000	-3.492473000	-1.452538000
C	-2.287380000	-2.958070000	0.511877000
H	-2.894753000	-2.324548000	1.192700000
H	-2.762599000	-2.910320000	-0.498347000
H	-2.385543000	-4.010143000	0.864811000
C	1.355451000	-0.395241000	0.507570000
H	1.545678000	-1.289406000	1.149578000
C	2.278757000	-0.526027000	-0.719314000
H	2.028934000	-1.407141000	-1.349178000
H	2.223711000	0.371077000	-1.374730000
H	3.342192000	-0.627484000	-0.401727000
C	1.736823000	0.825317000	1.363184000
H	1.135957000	0.897450000	2.293992000
H	2.808511000	0.766374000	1.663326000

H	1.620626000	1.789491000	0.819489000
C	-1.780079000	0.319770000	1.152363000
H	-2.757816000	-0.068326000	0.775290000
C	-1.687318000	-0.027285000	2.650002000
H	-1.754367000	-1.119982000	2.843118000
H	-0.734859000	0.330719000	3.099217000
H	-2.514648000	0.459266000	3.216537000
C	-1.794004000	1.841717000	0.935808000
H	-1.970436000	2.114913000	-0.127767000
H	-2.609610000	2.312952000	1.531904000
H	-0.843492000	2.318347000	1.264215000
H	-2.449308000	-2.148200000	-5.142910000
H	-1.249154000	1.223802000	-8.321024000

C1:

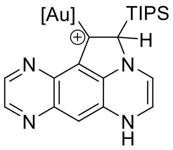

Au	-2.611885000	-2.724909000	-2.716120000
C	-1.814914000	-0.870274000	-2.682072000
C	-1.544763000	0.069411000	-1.578680000
H	-0.332877000	2.498104000	-6.574694000
C	-0.625581000	1.786704000	-5.789140000
C	-0.960043000	0.442684000	-6.210182000
N	-0.882473000	0.155292000	-7.532269000
C	-1.197511000	-1.085508000	-7.905890000
H	-1.137291000	-1.316959000	-8.985613000
H	-1.850306000	-3.097055000	-7.312757000
C	-1.590087000	-2.075651000	-6.979318000
N	-1.669041000	-1.821435000	-5.668891000
C	-1.369992000	-0.574352000	-5.267179000
C	-1.437833000	-0.196078000	-3.864302000
C	-1.081902000	1.131715000	-3.526084000
N	-1.168559000	1.333316000	-2.190452000
C	-0.960260000	2.613869000	-1.664200000
H	-1.122877000	2.768447000	-0.592875000
H	-0.420115000	4.635581000	-2.167766000
C	-0.588757000	3.609532000	-2.521842000
N	-0.413859000	3.385885000	-3.877873000
C	-0.684076000	2.142581000	-4.453054000
H	-0.136917000	4.162526000	-4.481321000
C	-3.495743000	-4.525720000	-3.039099000
N	-2.924604000	-5.764142000	-3.145008000
C	-3.859913000	-6.709730000	-3.558502000
H	-3.594812000	-7.764306000	-3.687571000
C	-5.047218000	-6.044432000	-3.717952000
H	-6.041670000	-6.393347000	-4.014716000
N	-4.801596000	-4.712869000	-3.400117000
C	-1.576826000	-6.087676000	-2.736396000
C	-1.405788000	-6.633661000	-1.440071000
C	-0.093889000	-6.956018000	-1.042858000
H	0.081877000	-7.380406000	-0.042424000
C	0.988497000	-6.750030000	-1.905805000
H	2.007769000	-7.014109000	-1.580214000
C	0.784579000	-6.211415000	-3.182956000

H	1.650369000	-6.054742000	-3.842386000
C	-0.502663000	-5.861369000	-3.633094000
C	-2.578934000	-6.819129000	-0.481940000
H	-3.507141000	-6.900780000	-1.088334000
C	-0.729571000	-5.283961000	-5.028421000
H	-1.499793000	-4.484948000	-4.931694000
C	-5.821865000	-3.693081000	-3.323191000
C	-6.015197000	-2.820663000	-4.423466000
C	-7.047435000	-1.871034000	-4.309248000
H	-7.236025000	-1.172886000	-5.138108000
C	-7.842238000	-1.795047000	-3.156888000
H	-8.649205000	-1.046800000	-3.097351000
C	-7.609663000	-2.657005000	-2.080022000
H	-8.232718000	-2.578122000	-1.174676000
C	-6.585878000	-3.622738000	-2.134506000
C	-5.158218000	-2.917831000	-5.681881000
H	-4.135338000	-3.218785000	-5.361497000
C	-6.334294000	-4.540314000	-0.943690000
H	-5.418355000	-5.133494000	-1.152383000
C	-2.481704000	-8.100835000	0.360087000
H	-3.422233000	-8.250229000	0.933812000
H	-1.653805000	-8.052233000	1.101324000
H	-2.319115000	-8.998191000	-0.275583000
C	-2.736259000	-5.574733000	0.412049000
H	-2.887441000	-4.658219000	-0.197929000
H	-1.828994000	-5.425718000	1.039088000
H	-3.609886000	-5.686923000	1.091498000
C	-1.271857000	-6.357352000	-5.994987000
H	-1.455322000	-5.915375000	-6.999882000
H	-2.229240000	-6.796746000	-5.643048000
H	-0.538159000	-7.185085000	-6.115458000
C	0.523138000	-4.623469000	-5.621349000
H	0.269390000	-4.121860000	-6.578803000
H	1.318848000	-5.367211000	-5.847530000
H	0.943996000	-3.854638000	-4.939006000
C	-5.696673000	-4.002961000	-6.636631000
H	-5.045771000	-4.087554000	-7.535273000
H	-6.724373000	-3.750390000	-6.980252000
H	-5.735349000	-5.002483000	-6.152997000
C	-5.016617000	-1.577138000	-6.416333000
H	-4.288023000	-1.676462000	-7.249042000
H	-4.650665000	-0.777840000	-5.735754000
H	-5.977535000	-1.242667000	-6.865586000
C	-7.493198000	-5.536408000	-0.753533000
H	-7.280888000	-6.227272000	0.092215000
H	-7.655181000	-6.149585000	-1.667084000
H	-8.446844000	-5.008770000	-0.529708000
C	-6.060487000	-3.735705000	0.339003000
H	-5.810850000	-4.420873000	1.178832000
H	-6.945815000	-3.138104000	0.648962000
H	-5.206585000	-3.038380000	0.196584000
Si	-0.089331000	-0.824482000	-0.485375000
C	1.353003000	-1.495756000	-1.553754000
H	2.186506000	-1.504636000	-0.807640000
C	1.788242000	-0.587337000	-2.720359000
H	1.860331000	0.486173000	-2.441752000

H	2.787772000	-0.899293000	-3.099666000
H	1.079846000	-0.672019000	-3.570938000
C	1.147479000	-2.939953000	-2.037208000
H	1.007787000	-3.660574000	-1.203478000
H	0.255259000	-3.028335000	-2.698063000
H	2.031816000	-3.282946000	-2.621675000
C	0.639058000	0.414745000	0.792552000
H	1.202665000	-0.313654000	1.427971000
C	1.685918000	1.406431000	0.253967000
H	2.520491000	0.888221000	-0.264216000
H	1.256447000	2.142420000	-0.459429000
H	2.132814000	1.985546000	1.094748000
C	-0.391431000	1.085943000	1.716155000
H	-0.996497000	0.343155000	2.277614000
H	0.118453000	1.730286000	2.468599000
H	-1.108361000	1.738773000	1.168630000
C	-0.990175000	-2.242870000	0.435715000
H	-1.085925000	-3.043068000	-0.335549000
C	-0.093293000	-2.780529000	1.567541000
H	0.937590000	-3.018090000	1.220764000
H	-0.008014000	-2.057811000	2.409543000
H	-0.522182000	-3.718560000	1.988157000
C	-2.408744000	-1.925862000	0.934031000
H	-3.107409000	-1.740009000	0.087794000
H	-2.821368000	-2.790110000	1.502600000
H	-2.440419000	-1.047415000	1.615383000
H	-2.335996000	0.191063000	-0.816242000

C2:

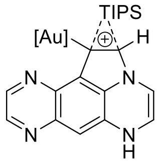

Au	-2.502224000	-2.788947000	-2.594706000
C	-1.821205000	-0.877390000	-2.415847000
C	-1.913796000	0.069468000	-1.332927000
H	-0.914695000	2.932319000	-6.104920000
C	-1.078570000	2.133612000	-5.367481000
C	-1.070899000	0.774651000	-5.852836000
N	-0.863175000	0.575870000	-7.179697000
C	-0.867163000	-0.681863000	-7.615007000
H	-0.699720000	-0.842196000	-8.696504000
H	-1.069609000	-2.815558000	-7.139861000
C	-1.070393000	-1.782135000	-6.750071000
N	-1.269836000	-1.621901000	-5.439859000
C	-1.282447000	-0.357374000	-4.972843000
C	-1.504775000	-0.075008000	-3.571493000
C	-1.515156000	1.270418000	-3.176681000
N	-1.779510000	1.373322000	-1.833954000
C	-1.927624000	2.646909000	-1.257343000
H	-2.225395000	2.712082000	-0.205550000
H	-1.828355000	4.756154000	-1.649728000
C	-1.719717000	3.738219000	-2.048155000
N	-1.374196000	3.634201000	-3.387955000
C	-1.304324000	2.394828000	-4.025391000

H	-1.244511000	4.484684000	-3.938293000
C	-3.414030000	-4.547698000	-3.025544000
N	-2.923100000	-5.821743000	-3.105276000
C	-3.901350000	-6.706327000	-3.551143000
H	-3.701730000	-7.777020000	-3.663520000
C	-5.035262000	-5.965344000	-3.760136000
H	-6.038765000	-6.250028000	-4.092874000
N	-4.714315000	-4.651760000	-3.438647000
C	-1.604011000	-6.225021000	-2.679775000
C	-1.462025000	-6.719504000	-1.360182000
C	-0.171905000	-7.113284000	-0.956448000
H	-0.016056000	-7.499647000	0.062690000
C	0.913646000	-7.031187000	-1.837121000
H	1.914343000	-7.351682000	-1.504721000
C	0.735603000	-6.548993000	-3.140337000
H	1.601753000	-6.493828000	-3.816305000
C	-0.527913000	-6.127069000	-3.595121000
C	-2.641452000	-6.792142000	-0.394969000
H	-3.575021000	-6.808654000	-0.999050000
C	-0.737483000	-5.630867000	-5.022811000
H	-1.555907000	-4.876692000	-4.996284000
C	-5.661054000	-3.561543000	-3.413606000
C	-5.746194000	-2.693935000	-4.531138000
C	-6.703101000	-1.664309000	-4.465925000
H	-6.806600000	-0.966428000	-5.309959000
C	-7.528751000	-1.508887000	-3.343374000
H	-8.275402000	-0.698512000	-3.321772000
C	-7.404208000	-2.371376000	-2.248870000
H	-8.050857000	-2.230722000	-1.367754000
C	-6.459291000	-3.415829000	-2.255166000
C	-4.857681000	-2.879330000	-5.757264000
H	-3.876434000	-3.264996000	-5.398913000
C	-6.316763000	-4.328531000	-1.042792000
H	-5.471566000	-5.024811000	-1.231290000
C	-2.641135000	-8.066512000	0.464323000
H	-3.585228000	-8.131809000	1.047886000
H	-1.804579000	-8.075476000	1.197275000
H	-2.557475000	-8.982171000	-0.160395000
C	-2.697991000	-5.526883000	0.481199000
H	-2.791887000	-4.612826000	-0.143977000
H	-1.775001000	-5.431852000	1.096507000
H	-3.569997000	-5.565222000	1.171058000
C	-1.188280000	-6.790001000	-5.936094000
H	-1.377016000	-6.421813000	-6.968904000
H	-2.122411000	-7.268484000	-5.571901000
H	-0.402235000	-7.575599000	-5.989654000
C	0.497025000	-4.932854000	-5.611014000
H	0.258199000	-4.532414000	-6.619200000
H	1.350872000	-5.633085000	-5.742530000
H	0.828869000	-4.083774000	-4.976400000
C	-5.456280000	-3.927128000	-6.718325000
H	-4.787039000	-4.078518000	-7.594187000
H	-6.447032000	-3.591383000	-7.097298000
H	-5.596707000	-4.913188000	-6.226038000
C	-4.577666000	-1.566213000	-6.501937000
H	-3.822271000	-1.736943000	-7.298329000

H	-4.181282000	-0.785522000	-5.817083000
H	-5.488542000	-1.166156000	-6.999362000
C	-7.578536000	-5.185581000	-0.833649000
H	-7.443503000	-5.876145000	0.027937000
H	-7.806298000	-5.797546000	-1.733844000
H	-8.468993000	-4.552547000	-0.623527000
C	-5.957799000	-3.525115000	0.219683000
H	-5.791378000	-4.209528000	1.080526000
H	-6.769600000	-2.819880000	0.503954000
H	-5.028072000	-2.935940000	0.061972000
Si	-0.108657000	-1.017167000	-0.713118000
C	1.245145000	-1.803889000	-1.832961000
H	2.138587000	-1.625977000	-1.183020000
C	1.514814000	-1.140099000	-3.193086000
H	1.458974000	-0.030549000	-3.174025000
H	2.529048000	-1.419834000	-3.557710000
H	0.783277000	-1.500894000	-3.944351000
C	1.081696000	-3.319372000	-2.010131000
H	1.092977000	-3.881393000	-1.052641000
H	0.126005000	-3.561793000	-2.529466000
H	1.909398000	-3.723173000	-2.635921000
C	0.776754000	0.397156000	0.253837000
H	1.595177000	-0.206882000	0.721613000
C	1.451558000	1.465385000	-0.622655000
H	2.154731000	1.022168000	-1.358862000
H	0.711344000	2.076028000	-1.183271000
H	2.039333000	2.168383000	0.011507000
C	-0.031835000	1.014090000	1.403123000
H	-0.395322000	0.251277000	2.123380000
H	0.595513000	1.735751000	1.975192000
H	-0.916815000	1.579665000	1.041437000
C	-0.805154000	-2.334777000	0.493140000
H	-0.952457000	-3.223803000	-0.161532000
C	0.277304000	-2.693859000	1.532267000
H	1.261207000	-2.925518000	1.067239000
H	0.438040000	-1.875711000	2.268438000
H	-0.032562000	-3.595722000	2.108662000
C	-2.154180000	-2.039747000	1.163152000
H	-2.975894000	-1.972839000	0.416160000
H	-2.426466000	-2.863709000	1.861134000
H	-2.140976000	-1.101225000	1.760672000
H	-2.463503000	-0.062013000	-0.396444000

C3:

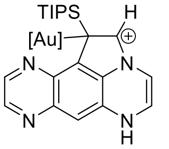

Au	-2.375068000	-2.888561000	-2.273477000
C	-1.411824000	-1.002792000	-1.790832000
C	-2.499760000	-0.466088000	-1.009438000
H	-2.806698000	2.080366000	-6.038572000
C	-2.493588000	1.443559000	-5.199140000
C	-1.358089000	0.587176000	-5.398146000
N	-0.763211000	0.598578000	-6.621738000
C	0.267758000	-0.217213000	-6.809619000

H	0.747875000	-0.204599000	-7.806051000
H	1.603222000	-1.755280000	-5.973833000
C	0.746796000	-1.080130000	-5.791764000
N	0.188871000	-1.113723000	-4.583609000
C	-0.856689000	-0.292234000	-4.355493000
C	-1.537632000	-0.285070000	-3.084360000
C	-2.638522000	0.567020000	-2.976929000
N	-3.202874000	0.451107000	-1.709983000
C	-4.332122000	1.228313000	-1.389515000
H	-4.758543000	1.124713000	-0.385434000
H	-5.693606000	2.706600000	-2.136602000
C	-4.821671000	2.070582000	-2.343133000
N	-4.270934000	2.179341000	-3.608079000
C	-3.152818000	1.431572000	-3.978529000
H	-4.667795000	2.844073000	-4.273516000
C	-3.451499000	-4.476720000	-2.877742000
N	-3.127802000	-5.795893000	-3.011192000
C	-4.213505000	-6.527564000	-3.480290000
H	-4.147385000	-7.607237000	-3.650698000
C	-5.246221000	-5.639811000	-3.648324000
H	-6.274529000	-5.777028000	-3.999108000
N	-4.757378000	-4.393361000	-3.278378000
C	-1.806133000	-6.335255000	-2.798640000
C	-1.482349000	-6.847538000	-1.520963000
C	-0.180920000	-7.355618000	-1.347517000
H	0.118345000	-7.758893000	-0.367497000
C	0.735798000	-7.365956000	-2.407251000
H	1.747124000	-7.774809000	-2.251056000
C	0.375573000	-6.863370000	-3.664306000
H	1.107700000	-6.883745000	-4.486832000
C	-0.905635000	-6.327218000	-3.891248000
C	-2.481400000	-6.818685000	-0.369872000
H	-3.494534000	-6.677844000	-0.807003000
C	-1.311959000	-5.820383000	-5.271067000
H	-2.244632000	-5.227027000	-5.154224000
C	-5.456871000	-3.137397000	-3.399158000
C	-5.361927000	-2.445162000	-4.630873000
C	-5.980259000	-1.182492000	-4.705010000
H	-5.922764000	-0.607520000	-5.642885000
C	-6.670344000	-0.652324000	-3.607095000
H	-7.153820000	0.334674000	-3.689072000
C	-6.762964000	-1.373958000	-2.409721000
H	-7.319366000	-0.947984000	-1.559306000
C	-6.156160000	-2.637249000	-2.276430000
C	-4.605994000	-3.012919000	-5.827106000
H	-4.364241000	-4.074700000	-5.604637000
C	-6.242861000	-3.407280000	-0.964540000
H	-5.751101000	-4.392509000	-1.115511000
C	-2.510705000	-8.130434000	0.431125000
H	-3.329186000	-8.100750000	1.183030000
H	-1.562345000	-8.298651000	0.987165000
H	-2.679390000	-9.008850000	-0.228900000
C	-2.211580000	-5.607741000	0.542243000
H	-2.244792000	-4.659107000	-0.038042000
H	-1.206672000	-5.681890000	1.014846000
H	-2.970178000	-5.546373000	1.354016000

C	-1.632723000	-7.006989000	-6.201738000
H	-1.976358000	-6.643632000	-7.195511000
H	-2.429688000	-7.656427000	-5.777892000
H	-0.732399000	-7.640913000	-6.361297000
C	-0.268814000	-4.883548000	-5.901753000
H	-0.652596000	-4.484400000	-6.866203000
H	0.686007000	-5.410494000	-6.120412000
H	-0.045098000	-4.022309000	-5.237468000
C	-5.457454000	-3.004985000	-7.108569000
H	-4.910470000	-3.510929000	-7.933871000
H	-5.682898000	-1.970349000	-7.449154000
H	-6.424541000	-3.532808000	-6.960013000
C	-3.269242000	-2.278111000	-6.032919000
H	-2.706120000	-2.721254000	-6.883691000
H	-2.632066000	-2.345365000	-5.123591000
H	-3.432065000	-1.200522000	-6.254711000
C	-7.701664000	-3.678791000	-0.556512000
H	-7.736055000	-4.299087000	0.365762000
H	-8.252840000	-4.218770000	-1.356772000
H	-8.251697000	-2.735205000	-0.344972000
C	-5.465146000	-2.683934000	0.149560000
H	-5.492184000	-3.270696000	1.094040000
H	-5.898759000	-1.680252000	0.360165000
H	-4.401094000	-2.548737000	-0.144968000
Si	0.222351000	-1.351447000	-0.781394000
C	1.561717000	-2.443653000	-1.621896000
H	2.053420000	-2.875988000	-0.716788000
C	2.664747000	-1.726930000	-2.415291000
H	3.175237000	-0.945381000	-1.811558000
H	3.444352000	-2.458480000	-2.731732000
H	2.258059000	-1.251443000	-3.331168000
C	0.960366000	-3.616468000	-2.407185000
H	0.239685000	-4.214199000	-1.803510000
H	0.430595000	-3.242890000	-3.309489000
H	1.755589000	-4.319570000	-2.746403000
C	0.878336000	0.406931000	-0.354113000
H	1.880229000	0.215722000	0.101581000
C	1.074977000	1.299709000	-1.591865000
H	1.684000000	0.815720000	-2.383155000
H	0.096014000	1.572183000	-2.047008000
H	1.579587000	2.253173000	-1.312340000
C	0.010515000	1.130983000	0.687560000
H	-0.084075000	0.566788000	1.639589000
H	0.437696000	2.130612000	0.933516000
H	-1.018915000	1.312927000	0.298427000
C	-0.157128000	-2.375113000	0.818389000
H	-0.121851000	-3.423221000	0.434791000
C	1.014027000	-2.217011000	1.811512000
H	2.009498000	-2.357933000	1.336002000
H	1.015155000	-1.212606000	2.289497000
H	0.934594000	-2.967910000	2.630872000
C	-1.495377000	-2.220818000	1.555219000
H	-2.361199000	-2.497457000	0.916552000
H	-1.528568000	-2.898062000	2.439892000
H	-1.651616000	-1.187355000	1.937962000
H	-2.754778000	-0.669829000	0.026831000

C5:

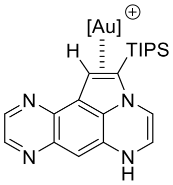

Au	-2.336658000	-2.544782000	-2.882701000
C	-1.761959000	-0.396447000	-3.363648000
C	-1.064281000	-0.736001000	-2.148939000
H	2.378876000	-1.127824000	-6.672079000
C	1.510381000	-0.950466000	-6.021696000
C	0.290782000	-0.510641000	-6.649015000
N	0.275840000	-0.355594000	-7.999699000
C	-0.863902000	0.058100000	-8.549482000
H	-0.875622000	0.183864000	-9.648454000
H	-2.955227000	0.678954000	-8.276055000
C	-2.027528000	0.330512000	-7.786550000
N	-2.053775000	0.173615000	-6.462262000
C	-0.909637000	-0.245556000	-5.882420000
C	-0.838183000	-0.466821000	-4.464095000
C	0.375435000	-0.874808000	-3.909820000
N	0.254220000	-1.026058000	-2.539305000
C	1.382601000	-1.469698000	-1.825585000
H	1.298041000	-1.585278000	-0.742298000
H	3.440481000	-2.047864000	-1.981675000
C	2.536809000	-1.713855000	-2.509262000
N	2.649177000	-1.557459000	-3.882352000
C	1.563631000	-1.136116000	-4.647251000
H	3.540149000	-1.751559000	-4.340614000
C	-3.000378000	-4.412243000	-3.200632000
N	-2.278393000	-5.384237000	-3.836561000
C	-3.050913000	-6.522526000	-4.030509000
H	-2.657448000	-7.408078000	-4.540611000
C	-4.287486000	-6.253948000	-3.499687000
H	-5.201042000	-6.855715000	-3.448385000
N	-4.233395000	-4.961232000	-2.991060000
C	-0.947885000	-5.165991000	-4.355789000
C	0.157069000	-5.286584000	-3.475328000
C	1.427419000	-5.004604000	-4.010366000
H	2.315783000	-5.087430000	-3.366998000
C	1.580069000	-4.619413000	-5.350262000
H	2.585413000	-4.400789000	-5.746023000
C	0.467768000	-4.513699000	-6.192157000
H	0.605078000	-4.202215000	-7.239544000
C	-0.828049000	-4.793242000	-5.715416000
C	-0.026202000	-5.736346000	-2.028809000
H	-0.960190000	-5.260758000	-1.652463000
C	-2.036735000	-4.657998000	-6.635033000
H	-2.920455000	-5.076638000	-6.107599000
C	-5.333800000	-4.257879000	-2.377882000
C	-5.999766000	-3.269273000	-3.140771000
C	-7.030883000	-2.555954000	-2.497293000
H	-7.574974000	-1.774812000	-3.051893000
C	-7.391017000	-2.842689000	-1.175861000
H	-8.204852000	-2.276423000	-0.694776000
C	-6.737900000	-3.860086000	-0.464738000

H	-7.051290000	-4.084199000	0.565714000
C	-5.689334000	-4.594567000	-1.046969000
C	-5.699753000	-3.023905000	-4.616506000
H	-4.805204000	-3.621895000	-4.892856000
C	-4.949031000	-5.685992000	-0.275319000
H	-4.653216000	-6.470015000	-1.006733000
C	-0.216897000	-7.265372000	-1.945975000
H	-0.381240000	-7.577847000	-0.891109000
H	0.686614000	-7.792665000	-2.324233000
H	-1.088021000	-7.613326000	-2.540598000
C	1.113118000	-5.295568000	-1.099401000
H	1.327710000	-4.210234000	-1.200216000
H	2.052239000	-5.855861000	-1.304783000
H	0.837567000	-5.496455000	-0.041845000
C	-1.867274000	-5.462192000	-7.935725000
H	-2.797944000	-5.410178000	-8.541928000
H	-1.648296000	-6.532576000	-7.729388000
H	-1.041377000	-5.061588000	-8.563761000
C	-2.345855000	-3.178216000	-6.919605000
H	-3.264722000	-3.083464000	-7.539371000
H	-1.511192000	-2.692018000	-7.470911000
H	-2.504685000	-2.611154000	-5.977308000
C	-6.867088000	-3.528231000	-5.487792000
H	-6.624322000	-3.407466000	-6.566677000
H	-7.800151000	-2.957838000	-5.283850000
H	-7.080618000	-4.603030000	-5.298754000
C	-5.364386000	-1.554854000	-4.918791000
H	-5.110362000	-1.425066000	-5.992818000
H	-4.484558000	-1.220324000	-4.328199000
H	-6.214984000	-0.876941000	-4.687008000
C	-5.814007000	-6.375112000	0.790249000
H	-5.273304000	-7.251921000	1.207506000
H	-6.778823000	-6.733576000	0.370602000
H	-6.039627000	-5.697494000	1.642921000
C	-3.647057000	-5.145454000	0.348030000
H	-3.078997000	-5.968874000	0.834933000
H	-3.874299000	-4.380178000	1.121778000
H	-2.987010000	-4.673834000	-0.411742000
Si	-1.589991000	-0.108308000	-0.404715000
C	-0.910390000	1.690148000	-0.307624000
H	-1.028831000	1.956953000	0.770993000
C	-1.706232000	2.714713000	-1.132020000
H	-2.758928000	2.805795000	-0.790260000
H	-1.245204000	3.725635000	-1.047463000
H	-1.717533000	2.453574000	-2.215390000
C	0.587906000	1.765611000	-0.650250000
H	1.206201000	1.056898000	-0.056433000
H	0.767372000	1.549584000	-1.727683000
H	0.983437000	2.788375000	-0.454519000
C	-3.501540000	-0.121306000	-0.181800000
H	-3.614163000	0.617040000	0.650794000
C	-4.358259000	0.380700000	-1.360127000
H	-3.976540000	1.309806000	-1.830909000
H	-4.442648000	-0.401746000	-2.146700000
H	-5.396662000	0.585030000	-1.013465000
C	-4.070080000	-1.464784000	0.304592000

H	-3.621106000	-1.805752000	1.259943000
H	-5.169758000	-1.386215000	0.462085000
H	-3.913559000	-2.266045000	-0.453119000
C	-0.702938000	-1.126170000	0.963171000
H	0.370053000	-0.836007000	0.857966000
C	-1.127149000	-0.670618000	2.373547000
H	-0.981858000	0.421212000	2.525591000
H	-2.195819000	-0.898413000	2.580842000
H	-0.525487000	-1.193980000	3.151429000
C	-0.798486000	-2.653348000	0.807467000
H	-0.553965000	-2.997810000	-0.222129000
H	-0.104084000	-3.170418000	1.509412000
H	-1.821240000	-3.020169000	1.030151000
H	-2.722582000	0.114261000	-3.397126000

C6:

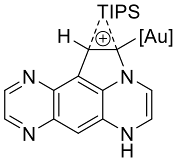

Au	-2.336412000	-3.107077000	-2.863743000
C	-1.836935000	-0.073507000	-3.671276000
C	-1.356501000	-1.334726000	-3.168580000
H	2.721362000	1.416302000	-6.046676000
C	1.765994000	1.142549000	-5.576529000
C	0.654109000	2.039490000	-5.766095000
N	0.850812000	3.160125000	-6.509988000
C	-0.191746000	3.968946000	-6.677214000
H	-0.029823000	4.880570000	-7.282416000
H	-2.310379000	4.396171000	-6.269423000
C	-1.464794000	3.701991000	-6.113354000
N	-1.691381000	2.615240000	-5.374494000
C	-0.650179000	1.776466000	-5.192099000
C	-0.791152000	0.574679000	-4.410303000
C	0.317104000	-0.266641000	-4.288479000
N	-0.012655000	-1.385188000	-3.547850000
C	0.966925000	-2.379974000	-3.365530000
H	0.671436000	-3.306835000	-2.860862000
H	2.997027000	-2.925374000	-3.779265000
C	2.209452000	-2.166856000	-3.883203000
N	2.550831000	-1.014960000	-4.580169000
C	1.605245000	-0.024557000	-4.841776000
H	3.497574000	-0.911054000	-4.947415000
C	-3.147082000	-4.957045000	-2.982675000
N	-2.488592000	-6.037105000	-3.501200000
C	-3.338857000	-7.128423000	-3.626699000
H	-2.993284000	-8.088657000	-4.024032000
C	-4.567427000	-6.718974000	-3.170792000
H	-5.525192000	-7.244326000	-3.091832000
N	-4.424381000	-5.392376000	-2.779159000
C	-1.087223000	-6.012153000	-3.841678000
C	-0.157757000	-6.442390000	-2.864618000
C	1.207181000	-6.391242000	-3.209312000
H	1.962067000	-6.727558000	-2.480298000
C	1.616059000	-5.929509000	-4.467884000

H	2.688730000	-5.908908000	-4.721898000
C	0.669414000	-5.499830000	-5.407398000
H	1.005894000	-5.135398000	-6.391222000
C	-0.708042000	-5.528794000	-5.117121000
C	-0.594059000	-6.956166000	-1.497803000
H	-1.695284000	-6.831430000	-1.420806000
C	-1.725060000	-5.056933000	-6.149303000
H	-2.733591000	-5.115242000	-5.685874000
C	-5.461797000	-4.552099000	-2.233852000
C	-6.124899000	-3.658542000	-3.106193000
C	-7.081489000	-2.798409000	-2.532010000
H	-7.620630000	-2.082627000	-3.173256000
C	-7.363506000	-2.847356000	-1.161742000
H	-8.117000000	-2.166674000	-0.733314000
C	-6.707977000	-3.768553000	-0.331409000
H	-6.960125000	-3.805214000	0.739192000
C	-5.739218000	-4.648721000	-0.847369000
C	-5.863862000	-3.636489000	-4.608998000
H	-5.111245000	-4.422387000	-4.836696000
C	-5.011403000	-5.655019000	0.042802000
H	-4.743002000	-6.525933000	-0.595607000
C	-0.290919000	-8.459113000	-1.350789000
H	-0.656971000	-8.835981000	-0.370548000
H	0.802271000	-8.659083000	-1.403568000
H	-0.780444000	-9.052865000	-2.153594000
C	0.027744000	-6.136166000	-0.354253000
H	-0.220871000	-5.057766000	-0.454594000
H	1.135639000	-6.235002000	-0.329685000
H	-0.360518000	-6.487527000	0.626802000
C	-1.732361000	-5.978790000	-7.382761000
H	-2.512959000	-5.654131000	-8.105321000
H	-1.940566000	-7.033737000	-7.099746000
H	-0.753651000	-5.958036000	-7.911468000
C	-1.495763000	-3.585076000	-6.538668000
H	-2.287279000	-3.244826000	-7.241926000
H	-0.513727000	-3.439053000	-7.040473000
H	-1.528371000	-2.930166000	-5.639662000
C	-7.140989000	-3.990564000	-5.394148000
H	-6.921844000	-4.048645000	-6.482956000
H	-7.934211000	-3.223780000	-5.252239000
H	-7.556895000	-4.969970000	-5.071929000
C	-5.266788000	-2.297728000	-5.077518000
H	-5.090735000	-2.316824000	-6.175721000
H	-4.290641000	-2.103623000	-4.578670000
H	-5.945765000	-1.444582000	-4.857191000
C	-5.886763000	-6.191375000	1.186123000
H	-5.366291000	-7.026791000	1.702585000
H	-6.863291000	-6.570534000	0.814439000
H	-6.089998000	-5.412355000	1.953582000
C	-3.688939000	-5.082500000	0.592771000
H	-3.153391000	-5.855994000	1.186948000
H	-3.882060000	-4.212008000	1.257129000
H	-3.012163000	-4.746231000	-0.222233000
Si	-1.666327000	0.055225000	-1.383090000
C	-1.341168000	1.951820000	-1.325878000
H	-1.446570000	2.100964000	-0.220757000

C	-2.419362000	2.815662000	-1.998399000
H	-3.435825000	2.599288000	-1.604137000
H	-2.218513000	3.893796000	-1.800910000
H	-2.439292000	2.685936000	-3.103214000
C	0.078483000	2.394583000	-1.712395000
H	0.869420000	1.819672000	-1.183519000
H	0.261873000	2.290946000	-2.802177000
H	0.226526000	3.469418000	-1.460337000
C	-3.457002000	-0.235672000	-0.744717000
H	-3.579866000	0.647133000	-0.070111000
C	-4.563889000	-0.158298000	-1.809380000
H	-4.531078000	0.777234000	-2.407443000
H	-4.510080000	-1.025725000	-2.504064000
H	-5.563658000	-0.205966000	-1.322026000
C	-3.628698000	-1.502847000	0.103385000
H	-2.948931000	-1.528029000	0.981056000
H	-4.673525000	-1.576134000	0.481925000
H	-3.440286000	-2.415746000	-0.505935000
C	-0.236764000	-0.745813000	-0.353766000
H	0.668525000	-0.535784000	-0.972206000
C	-0.072790000	-0.000242000	0.989851000
H	0.180427000	1.072902000	0.867374000
H	-0.994203000	-0.065295000	1.611557000
H	0.748961000	-0.465656000	1.580745000
C	-0.290887000	-2.258945000	-0.099796000
H	-0.593564000	-2.838728000	-0.999362000
H	0.705206000	-2.632374000	0.229506000
H	-1.017638000	-2.512558000	0.700511000
H	-2.892434000	0.196838000	-3.706126000

C7:

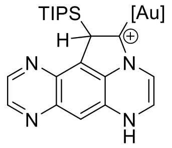

Au	-2.280422000	-3.241175000	-3.045468000
C	-1.570080000	-0.101032000	-3.388222000
C	-1.226839000	-1.543408000	-3.373963000
H	3.091029000	1.337663000	-5.727682000
C	2.124220000	1.083560000	-5.269858000
C	1.122485000	2.107075000	-5.217476000
N	1.423785000	3.326587000	-5.745289000
C	0.460785000	4.237605000	-5.757654000
H	0.708191000	5.230168000	-6.178660000
H	-1.638451000	4.743834000	-5.321847000
C	-0.847427000	3.973715000	-5.263581000
N	-1.167349000	2.808798000	-4.712718000
C	-0.194775000	1.867083000	-4.651915000
C	-0.441661000	0.578905000	-4.077353000
C	0.544358000	-0.381744000	-4.226865000
N	0.052591000	-1.629336000	-3.808482000
C	0.911374000	-2.740518000	-3.923477000
H	0.520683000	-3.719005000	-3.617086000
H	2.851707000	-3.403154000	-4.531351000
C	2.164321000	-2.551982000	-4.429467000
N	2.641307000	-1.322219000	-4.847224000

C	1.829307000	-0.191042000	-4.799482000
H	3.582220000	-1.248800000	-5.236332000
C	-3.174898000	-5.067492000	-3.034845000
N	-2.562855000	-6.195589000	-3.509598000
C	-3.438327000	-7.273896000	-3.525706000
H	-3.130625000	-8.264502000	-3.876821000
C	-4.636032000	-6.808127000	-3.044134000
H	-5.600217000	-7.304992000	-2.892357000
N	-4.450076000	-5.462883000	-2.746265000
C	-1.192736000	-6.229470000	-3.960659000
C	-0.181692000	-6.531008000	-3.016638000
C	1.147540000	-6.553328000	-3.483025000
H	1.963021000	-6.786998000	-2.780139000
C	1.445137000	-6.291545000	-4.827786000
H	2.490721000	-6.332142000	-5.175558000
C	0.421113000	-5.983150000	-5.733249000
H	0.670337000	-5.769026000	-6.784888000
C	-0.924737000	-5.941746000	-5.320690000
C	-0.492986000	-6.767457000	-1.544013000
H	-1.594582000	-6.872077000	-1.440884000
C	-2.022851000	-5.561936000	-6.306231000
H	-2.997405000	-5.636288000	-5.777115000
C	-5.464299000	-4.577141000	-2.229337000
C	-6.115244000	-3.707981000	-3.135205000
C	-7.068725000	-2.821620000	-2.596767000
H	-7.598160000	-2.124278000	-3.265756000
C	-7.361256000	-2.822630000	-1.228267000
H	-8.112405000	-2.122517000	-0.828051000
C	-6.718794000	-3.720939000	-0.363367000
H	-6.979858000	-3.720591000	0.705531000
C	-5.754280000	-4.626176000	-0.842384000
C	-5.855775000	-3.743251000	-4.638109000
H	-5.096270000	-4.530159000	-4.836842000
C	-5.046376000	-5.611905000	0.086337000
H	-4.825792000	-6.525544000	-0.509090000
C	0.140363000	-8.061568000	-1.006183000
H	-0.179601000	-8.236167000	0.044227000
H	1.251568000	-8.011835000	-1.007779000
H	-0.161458000	-8.944738000	-1.610166000
C	-0.075545000	-5.539756000	-0.712158000
H	-0.578821000	-4.618990000	-1.082586000
H	1.023823000	-5.373523000	-0.763994000
H	-0.351256000	-5.677581000	0.356313000
C	-2.078044000	-6.525430000	-7.504725000
H	-2.924060000	-6.259034000	-8.175410000
H	-2.217683000	-7.577752000	-7.174152000
H	-1.145807000	-6.481799000	-8.110200000
C	-1.866191000	-4.096741000	-6.756151000
H	-2.708050000	-3.802528000	-7.421073000
H	-0.918419000	-3.942927000	-7.318368000
H	-1.862775000	-3.413618000	-5.876608000
C	-7.131490000	-4.140556000	-5.404977000
H	-6.915105000	-4.238209000	-6.491606000
H	-7.932404000	-3.377340000	-5.289450000
H	-7.535993000	-5.111204000	-5.043961000
C	-5.272591000	-2.418839000	-5.160719000

H	-5.099080000	-2.480081000	-6.257799000
H	-4.297496000	-2.196872000	-4.671351000
H	-5.957771000	-1.563317000	-4.971459000
C	-5.912732000	-6.053384000	1.275671000
H	-5.410888000	-6.880191000	1.823377000
H	-6.911807000	-6.413797000	0.947731000
H	-6.066915000	-5.228262000	2.005489000
C	-3.691112000	-5.062773000	0.575739000
H	-3.167175000	-5.826987000	1.191829000
H	-3.836438000	-4.156496000	1.202916000
H	-3.025579000	-4.786964000	-0.269725000
Si	-1.712405000	0.389420000	-1.412191000
C	-1.749648000	2.295299000	-1.170560000
H	-1.987565000	2.319324000	-0.076489000
C	-2.904427000	3.013615000	-1.889982000
H	-3.895855000	2.601614000	-1.602678000
H	-2.911385000	4.094237000	-1.616388000
H	-2.795338000	2.949095000	-2.994106000
C	-0.406191000	3.022464000	-1.347011000
H	0.413304000	2.554854000	-0.759475000
H	-0.082439000	3.052979000	-2.406583000
H	-0.494575000	4.078711000	-1.003591000
C	-3.439030000	-0.238135000	-0.827539000
H	-3.732026000	0.566136000	-0.109755000
C	-4.494026000	-0.263473000	-1.946899000
H	-4.532422000	0.678540000	-2.535568000
H	-4.304401000	-1.104753000	-2.650195000
H	-5.508377000	-0.433884000	-1.520912000
C	-3.452096000	-1.568401000	-0.063482000
H	-2.797022000	-1.558706000	0.833644000
H	-4.486044000	-1.811775000	0.272767000
H	-3.122568000	-2.407904000	-0.719212000
C	-0.196830000	-0.262742000	-0.414826000
H	0.681380000	0.216780000	-0.911192000
C	-0.272964000	0.290842000	1.027283000
H	-0.307019000	1.399426000	1.069458000
H	-1.166408000	-0.094295000	1.568234000
H	0.621177000	-0.031850000	1.608099000
C	0.067237000	-1.777243000	-0.368016000
H	0.258766000	-2.215563000	-1.366815000
H	0.964299000	-1.985850000	0.259949000
H	-0.781776000	-2.334947000	0.079260000
H	-2.594631000	0.145193000	-3.713058000

D1:

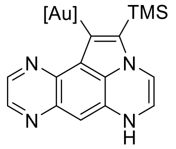

Si	-2.862132000	0.517902000	1.731618000
H	-0.457149000	0.188252000	-5.375302000
C	-1.981436000	-1.259057000	-0.412829000
C	-2.304137000	0.053836000	-0.008012000
C	-0.764348000	-0.146661000	-4.373981000
C	-0.664267000	-1.550698000	-4.080905000
N	-0.196346000	-2.377071000	-5.052886000

C	-0.114142000	-3.677086000	-4.763988000
H	0.271147000	-4.346385000	-5.557907000
H	-0.430346000	-5.284778000	-3.303860000
C	-0.497682000	-4.199795000	-3.513076000
N	-0.961180000	-3.415219000	-2.533216000
C	-1.050884000	-2.093808000	-2.780649000
C	-1.529911000	-1.165681000	-1.779520000
C	-1.594090000	0.185341000	-2.146204000
N	-2.062694000	0.928349000	-1.079253000
C	-2.199917000	2.312362000	-1.251467000
H	-2.578236000	2.902389000	-0.411831000
H	-1.960474000	3.950720000	-2.623618000
C	-1.865176000	2.870322000	-2.450321000
N	-1.388792000	2.112397000	-3.517446000
C	-1.233263000	0.732529000	-3.402718000
C	-3.451767000	2.322268000	1.906933000
H	-3.852931000	2.460082000	2.936917000
H	-4.270468000	2.574520000	1.196924000
H	-2.628128000	3.059225000	1.777264000
C	-4.315043000	-0.591305000	2.239824000
H	-5.223745000	-0.332272000	1.651986000
H	-4.074438000	-1.659234000	2.035730000
H	-4.551027000	-0.473871000	3.321633000
C	-1.391657000	0.294160000	2.915217000
H	-0.611198000	1.059834000	2.706888000
H	-1.702653000	0.395088000	3.979776000
H	-0.927374000	-0.705313000	2.775130000
Au	-2.105151000	-2.991735000	0.641331000
C	-2.245806000	-4.770813000	1.654968000
N	-1.751180000	-5.092307000	2.894822000
C	-2.053330000	-6.402371000	3.233748000
H	-1.746325000	-6.845950000	4.187341000
C	-2.755234000	-6.927172000	2.180488000
H	-3.179752000	-7.925029000	2.024431000
N	-2.864424000	-5.920859000	1.233615000
C	-1.040066000	-4.170729000	3.811373000
H	-0.722958000	-4.819973000	4.656274000
C	-2.003209000	-3.108833000	4.351242000
C	0.215611000	-3.586288000	3.159765000
H	0.892490000	-4.393834000	2.806663000
H	-0.055707000	-2.953237000	2.284481000
H	0.762752000	-2.960144000	3.896802000
H	-2.382982000	-2.473668000	3.523164000
H	-1.480847000	-2.454979000	5.082180000
H	-2.871613000	-3.583203000	4.857887000
C	-3.535112000	-6.122214000	-0.074767000
H	-3.975293000	-7.140054000	0.006793000
C	-2.512977000	-6.114921000	-1.213567000
C	-4.676012000	-5.122318000	-0.280832000
H	-5.394847000	-5.153937000	0.566401000
H	-4.276472000	-4.085983000	-0.366692000
H	-5.219992000	-5.368815000	-1.217862000
H	-2.042633000	-5.110095000	-1.324313000
H	-3.019042000	-6.366883000	-2.170850000
H	-1.716024000	-6.869028000	-1.031893000
H	-1.143690000	2.571541000	-4.394002000

D2:

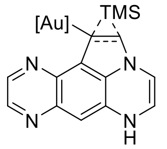

Si	-2.812340000	-0.000187000	1.228308000
H	0.722044000	0.897177000	-4.146565000
C	-2.421314000	-1.256180000	-0.321402000
C	-3.644186000	-0.582744000	-0.741971000
C	0.116401000	0.422188000	-3.360828000
C	0.785177000	-0.471207000	-2.456261000
N	2.117462000	-0.684643000	-2.628072000
C	2.715732000	-1.515677000	-1.775895000
H	3.801176000	-1.685411000	-1.914940000
H	2.541861000	-2.870986000	-0.056985000
C	2.018339000	-2.171798000	-0.736935000
N	0.711320000	-1.988979000	-0.537324000
C	0.071413000	-1.139025000	-1.371131000
C	-1.331618000	-0.859783000	-1.228075000
C	-1.913721000	0.002975000	-2.150231000
N	-3.264159000	0.165440000	-1.846885000
C	-4.002561000	1.071025000	-2.621675000
H	-5.056513000	1.207327000	-2.349240000
H	-3.938160000	2.421115000	-4.296798000
C	-3.393996000	1.711884000	-3.658325000
N	-2.046826000	1.510131000	-3.977474000
C	-1.246247000	0.667025000	-3.214473000
C	-2.994790000	1.851425000	0.799224000
H	-2.760932000	2.471256000	1.695025000
H	-4.019658000	2.096887000	0.447927000
H	-2.279439000	2.140567000	-0.003225000
C	-4.168383000	-0.553317000	2.436320000
H	-5.150747000	-0.630625000	1.926052000
H	-3.924445000	-1.536136000	2.896397000
H	-4.246724000	0.197982000	3.256122000
C	-1.153812000	-0.058671000	2.187205000
H	-0.299585000	0.203919000	1.525015000
H	-1.173300000	0.662812000	3.036479000
H	-0.960470000	-1.076910000	2.588856000
Au	-2.492050000	-3.082063000	0.632295000
C	-2.669183000	-4.946169000	1.420877000
N	-1.735617000	-5.710988000	2.069357000
C	-2.262448000	-6.941404000	2.430429000
H	-1.672355000	-7.696861000	2.960874000
C	-3.563160000	-6.955664000	1.997713000
H	-4.337906000	-7.726646000	2.072720000
N	-3.792589000	-5.733160000	1.386264000
C	-0.342569000	-5.306307000	2.380185000
H	0.084896000	-6.198973000	2.886576000
C	-0.322471000	-4.132699000	3.362978000
C	0.462374000	-5.033515000	1.107203000
H	0.374343000	-5.879577000	0.391695000
H	0.127551000	-4.095929000	0.606844000
H	1.535266000	-4.913085000	1.373293000
H	-0.759057000	-3.226100000	2.889430000
H	0.725425000	-3.902780000	3.651906000

H	-0.899860000	-4.368518000	4.282973000
C	-5.092349000	-5.367581000	0.769774000
H	-5.737752000	-6.250510000	0.968580000
C	-4.955284000	-5.195955000	-0.746283000
C	-5.714590000	-4.147084000	1.454055000
H	-5.781741000	-4.294969000	2.553582000
H	-5.111130000	-3.233573000	1.254118000
H	-6.738731000	-3.980409000	1.056518000
H	-4.317453000	-4.313992000	-0.979893000
H	-5.957934000	-5.030310000	-1.195642000
H	-4.502399000	-6.097767000	-1.212119000
H	-1.628688000	2.038152000	-4.742857000

D3:

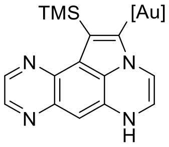

Si	-1.191355000	-0.345298000	1.986116000
H	0.651681000	1.494177000	-4.415063000
C	-1.182610000	-0.737702000	0.143332000
C	-1.744136000	-1.868205000	-0.494850000
C	0.295091000	1.074966000	-3.463066000
C	0.566266000	1.816890000	-2.263000000
N	1.245376000	2.989480000	-2.374113000
C	1.489261000	3.666301000	-1.251289000
H	2.042847000	4.620676000	-1.348421000
H	1.287090000	3.790148000	0.931726000
C	1.071646000	3.208832000	0.015463000
N	0.401938000	2.060648000	0.162469000
C	0.134389000	1.344572000	-0.948830000
C	-0.584750000	0.091126000	-0.883144000
C	-0.805025000	-0.564828000	-2.100446000
N	-1.498908000	-1.736232000	-1.862685000
C	-1.823838000	-2.549734000	-2.955271000
H	-2.374867000	-3.475028000	-2.743356000
H	-1.680124000	-2.766250000	-5.091762000
C	-1.446812000	-2.159911000	-4.206025000
N	-0.746502000	-0.975425000	-4.436738000
C	-0.397787000	-0.129249000	-3.384643000
C	-2.110051000	1.284526000	2.309415000
H	-2.106617000	1.532303000	3.395380000
H	-3.168437000	1.218290000	1.970998000
H	-1.610815000	2.108633000	1.756364000
C	-2.079947000	-1.685475000	3.011104000
H	-3.148274000	-1.800952000	2.725330000
H	-1.593114000	-2.681255000	2.922872000
H	-2.046320000	-1.385342000	4.084114000
C	0.584870000	-0.242995000	2.649045000
H	1.140170000	0.549865000	2.103827000
H	0.588154000	0.003845000	3.735228000
H	1.117548000	-1.210548000	2.510567000
Au	-2.744710000	-3.479962000	0.224314000
C	-3.752204000	-5.101675000	0.974104000
N	-3.226087000	-6.290838000	1.407363000
C	-4.220182000	-7.140497000	1.867736000

H	-4.001525000	-8.140303000	2.258832000
C	-5.407617000	-6.471236000	1.721298000
H	-6.434992000	-6.768748000	1.958723000
N	-5.101427000	-5.233871000	1.176072000
C	-1.789419000	-6.659323000	1.386703000
H	-1.768084000	-7.683345000	1.818285000
C	-0.963763000	-5.735273000	2.285777000
C	-1.261102000	-6.724554000	-0.049523000
H	-1.875661000	-7.410843000	-0.671400000
H	-1.279337000	-5.712183000	-0.512668000
H	-0.212604000	-7.092333000	-0.047968000
H	-0.952785000	-4.701302000	1.874063000
H	0.083202000	-6.103330000	2.340528000
H	-1.380678000	-5.698212000	3.315072000
C	-6.128305000	-4.213858000	0.851655000
H	-7.084988000	-4.686781000	1.162193000
C	-6.181409000	-3.950562000	-0.656300000
C	-5.926644000	-2.938492000	1.674238000
H	-5.877468000	-3.164667000	2.761003000
H	-4.981386000	-2.431043000	1.378223000
H	-6.771964000	-2.239843000	1.495546000
H	-5.229820000	-3.485135000	-0.999203000
H	-7.016886000	-3.255469000	-0.887445000
H	-6.339492000	-4.894090000	-1.222134000
H	-0.483815000	-0.721827000	-5.388258000

5.2 Calculations of the Optoelectronic Properties

Calculations were performed using Gaussian 16. TMS groups were used instead of TIPS groups to simplify calculations. First, the gas-phase ground-state equilibrium geometry of the molecules was optimized at the B3LYP/def2-SVP level of theory. Afterwards, the received geometries were refined using the B3LYP/def2-TZVP level of theory. FMO calculations were performed starting from the optimized geometries on the B3LYP/def2-TZVP level of theory.[8]

5.2.1 FMO Calculations

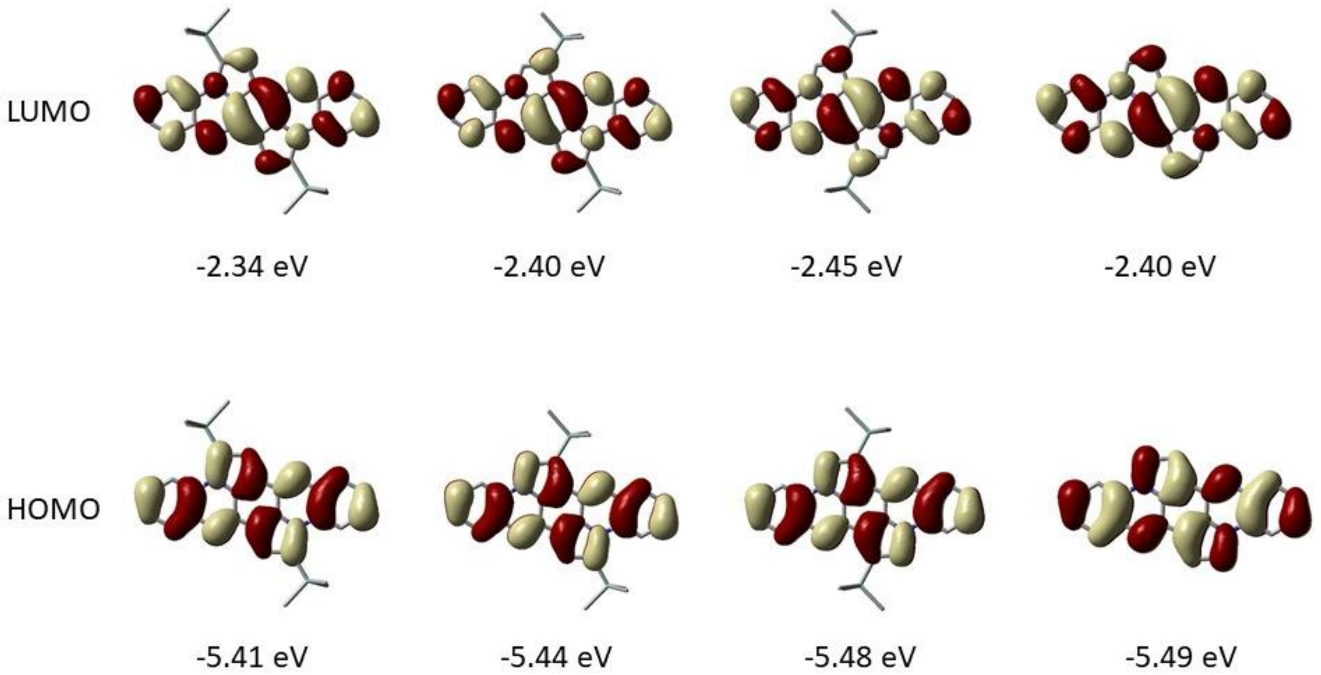

Figure S15. Calculated FMOs. From left to right: **1, 2, 3, 4**.

5.2.2 Calculated and Measured Bond Lengths

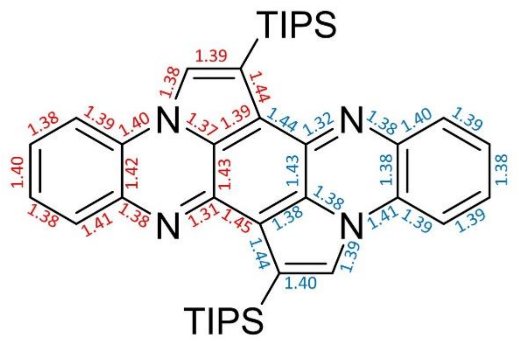

Figure S16. Comparison of calculated (red) and measured (blue) bond lengths of **3**. For calculations TMS groups were used

5.2.3 Coordinates of the Optimized Geometries

TIPSTAP-H$_2$*:

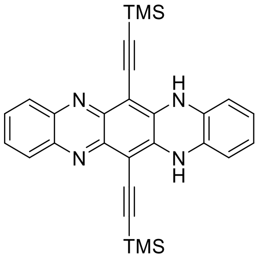

C	-0.71003500	6.07606400	0.00080900
C	0.70630700	6.07650100	0.00082100
C	1.40450700	4.89863600	0.00067900
C	0.71372300	3.66144300	0.00051900
C	-0.71595800	3.66100200	0.00050700
C	-1.40750700	4.89776700	0.00065600
N	1.40676400	2.50536900	0.00038100
C	0.72210100	1.37551100	0.00023600
C	-0.72292400	1.37506500	0.00022500
N	-1.40828400	2.50449900	0.00035900
C	1.43028800	0.11793200	0.00009000
C	0.71892600	-1.06952900	-0.00005300
C	-0.71824400	-1.06997200	-0.00006400
C	-1.43033700	0.11705000	0.00006700
N	1.35485800	-2.28480900	-0.00019100
C	0.70311500	-3.51185500	-0.00034500
C	-0.70092700	-3.51228800	-0.00035300
N	-1.35342700	-2.28564400	-0.00020800
C	1.39257700	-4.71889800	-0.00048800
C	0.69644000	-5.92323500	-0.00063900
C	-0.69276400	-5.92366300	-0.00064500
C	-1.38964300	-4.71975600	-0.00050300
C	2.84396700	0.07048000	0.00010600
C	4.05329500	-0.05355600	0.00008300
C	-2.84398700	0.06872400	0.00005500
C	-4.05325400	-0.05590200	0.00001500
Si	5.89149100	-0.10883300	-0.00010900
C	6.51573700	0.75865300	1.54389200
C	6.51546600	0.76167600	-1.54251200
C	6.43196000	-1.91066700	-0.00189100
Si	-5.89145600	-0.11092900	-0.00013900
C	-6.51533500	0.75822100	-1.54335100
C	-6.51558400	0.75805900	1.54306200
C	-6.43214900	-1.91269700	-0.00027600
H	-1.24125500	7.01958900	0.00092300
H	1.23694400	7.02035500	0.00094300
H	2.48652000	4.87476500	0.00068400
H	-2.48950600	4.87322700	0.00064500
H	2.36485100	-2.26229000	-0.00019400
H	-2.36343300	-2.26374700	-0.00022000
H	2.47598700	-4.71109600	-0.00048300
H	1.24318500	-6.85655000	-0.00075000
H	-1.23893300	-6.85731600	-0.00076200
H	-2.47305800	-4.71262200	-0.00050700
H	6.15314600	0.26569300	2.44818000

H	7.60852500	0.75923100	1.57472000
H	6.17677100	1.79611600	1.57203100
H	6.15303200	0.27026000	-2.44770200
H	7.60825400	0.76266300	-1.57333900
H	6.17616100	1.79907800	-1.56874300
H	7.52235400	-1.98691400	-0.00220300
H	6.06310100	-2.43638700	0.88168800
H	6.06269500	-2.43476500	-0.88626100
H	-6.15290300	0.26596300	-2.44808500
H	-7.60812200	0.75924100	-1.57421400
H	-6.17597400	1.79558000	-1.57051400
H	-6.15312700	0.26583000	2.44780100
H	-7.60837400	0.75889300	1.57385000
H	-6.17640100	1.79547500	1.57028700
H	-6.06306900	-2.43761200	-0.88424100
H	-6.06324100	-2.43769500	0.88371100
H	-7.52255300	-1.98880400	-0.00038600

TIPSTAP*:

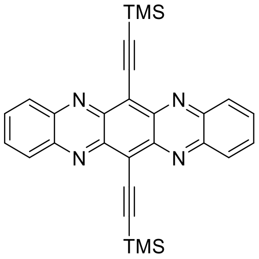

C	0.71557900	5.93072900	-0.00032200
C	-0.71536300	5.93075600	-0.00032600
C	-1.41618600	4.76605900	-0.00026100
C	-0.72473600	3.51465400	-0.00018800
C	0.72486400	3.51462800	-0.00018400
C	1.41636000	4.76600800	-0.00025400
N	-1.40888000	2.38318000	-0.00012800
C	-0.72465300	1.22496500	-0.00006600
C	0.72469700	1.22493900	-0.00006100
N	1.40896700	2.38312800	-0.00011900
C	-1.44780400	0.00494100	-0.00000900
C	-0.72477900	-1.21523200	0.00005200
C	0.72473500	-1.21525900	0.00005600
C	1.44780400	0.00488800	-0.00000100
N	-1.40895700	-2.37348800	0.00010800
C	-0.72483100	-3.50496800	0.00016700
C	0.72470200	-3.50499500	0.00017200
N	1.40887000	-2.37354000	0.00011700
C	-1.41621700	-4.75640100	0.00023000
C	-0.71556400	-5.92118500	0.00029300
C	0.71534600	-5.92121100	0.00029700
C	1.41604200	-4.75645300	0.00023900
C	2.85872800	0.00512200	0.00000800
C	4.07273100	0.00498300	0.00001000
C	-2.85872800	0.00522600	-0.00001300
C	-4.07273000	0.00512100	-0.00001600
Si	-5.91013200	-0.00560600	0.00001200
C	-6.49820400	-0.90117300	1.54399800
C	-6.49824500	-0.90469000	-1.54191100

C	-6.52133000	1.77065100	-0.00199900
Si	5.91013300	-0.00572400	0.00000300
C	6.49823700	-0.90096800	1.54416500
C	6.52131400	1.77053800	-0.00239000
C	6.49823000	-0.90512800	-1.54174000
H	1.23911100	6.87842700	-0.00037700
H	-1.23886000	6.87847200	-0.00038300
H	-2.49781400	4.74268300	-0.00026500
H	2.49798700	4.74259200	-0.00025200
H	-2.49784900	-4.73306400	0.00022500
H	-1.23911900	-6.86886500	0.00034000
H	1.23886600	-6.86891000	0.00034800
H	2.49767500	-4.73315600	0.00024000
H	-6.14799800	-0.39880500	2.44799100
H	-7.59031900	-0.93617400	1.57953400
H	-6.12659700	-1.92755400	1.56763000
H	-6.14809300	-0.40436200	-2.44705600
H	-7.59035900	-0.93980900	-1.57732500
H	-6.12659800	-1.93110700	-1.56323100
H	-7.61391300	1.80662300	-0.00222400
H	-6.16644700	2.30835400	0.87957600
H	-6.16614500	2.30646900	-0.88460100
H	6.12664100	-1.92734800	1.56800000
H	7.59035200	-0.93595000	1.57969700
H	6.14803400	-0.39842800	2.44806200
H	6.16627500	2.30611600	-0.88519600
H	7.61389600	1.80651900	-0.00243800
H	6.16627400	2.30847500	0.87898000
H	7.59034900	-0.93987500	-1.57737900
H	6.12694100	-1.93168400	-1.56262600
H	6.14770400	-0.40523600	-2.44698000

1*:

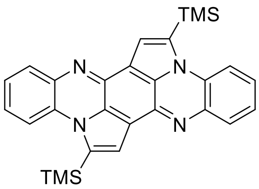

C	-5.20779400	3.00558700	-0.00001800
C	-4.36761900	4.12058700	-0.00005800
C	-3.00077500	3.94257500	-0.00007700
C	-2.42373900	2.66068300	-0.00006600
C	-3.29474100	1.53072000	-0.00003600
C	-4.67500300	1.72874000	-0.00000400
N	-1.04739100	2.58547700	-0.00006600
C	-0.51945700	1.39178000	-0.00003600
C	-1.32313200	0.21244500	-0.00001100
N	-2.69625400	0.26165900	-0.00001900
C	0.90069300	1.10912700	-0.00001000
C	1.32313400	-0.21244600	0.00003500
C	0.51945900	-1.39178100	0.00005400
C	-0.90069100	-1.10912900	0.00002400
N	2.69625600	-0.26166000	0.00003200
C	3.29474200	-1.53072300	0.00004300
C	2.42373900	-2.66068500	0.00005800
N	1.04739200	-2.58547800	0.00006000

C	4.67500400	-1.72874500	0.00002800
C	5.20779400	-3.00559400	0.00003500
C	4.36761700	-4.12059200	0.00005300
C	3.00077400	-3.94257800	0.00006100
C	-2.07753100	-1.88632200	-0.00002300
C	-3.19394800	-1.05558900	0.00001300
C	2.07753200	1.88632100	-0.00000500
C	3.19395000	1.05558800	0.00000600
Si	-4.93849600	-1.78074500	0.00000700
C	-4.73182700	-3.65128200	0.00000400
C	-5.89738500	-1.32828300	1.55917300
C	-5.89736600	-1.32827500	-1.55916700
Si	4.93849600	1.78074900	-0.00000700
C	5.89740400	1.32817200	-1.55912700
C	4.73181900	3.65128400	-0.00014100
C	5.89735000	1.32840400	1.55921500
H	-6.28269100	3.13197700	0.00000600
H	-4.78673600	5.11835700	-0.00006700
H	-2.32072500	4.78395300	-0.00009900
H	-5.34153300	0.88446000	0.00003700
H	5.34153600	-0.88446700	0.00001100
H	6.28269000	-3.13198500	0.00002400
H	4.78673300	-5.11836300	0.00005500
H	2.32072300	-4.78395500	0.00007200
H	-2.11150300	-2.96220400	-0.00003800
H	2.11150400	2.96220300	-0.00002100
H	-5.72045100	-4.11799900	0.00002200
H	-4.20119200	-4.00917600	-0.88418600
H	-4.20116100	-4.00917800	0.88417400
H	-6.87413100	-1.81986300	1.54611500
H	-6.06762500	-0.25977800	1.69097700
H	-5.36050400	-1.68456900	2.44159500
H	-5.36046300	-1.68453300	-2.44158800
H	-6.87410200	-1.81987400	-1.54613500
H	-6.06762500	-0.25977100	-1.69095300
H	6.87414100	1.81976900	-1.54610700
H	6.06766200	0.25965700	-1.69083300
H	5.36052200	1.68436900	-2.44158500
H	5.72044200	4.11800600	-0.00012800
H	4.20112800	4.00923600	0.88399200
H	4.20120600	4.00911800	-0.88436800
H	5.36044700	1.68475300	2.44159900
H	6.06759200	0.25991000	1.69110400
H	6.87409300	1.81998700	1.54614300

2*:

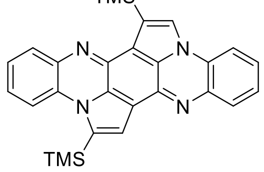

C	5.61885700	-3.44028200	0.00010300
C	4.76689100	-4.54860400	0.00013500
C	3.39836400	-4.37038400	0.00012900
C	2.82808500	-3.08594200	0.00008500
C	3.71443700	-1.97327400	0.00005200

C	5.09622700	-2.15916800	0.00006400
N	1.45115700	-2.97966300	0.00009100
C	0.94052600	-1.77592200	0.00005700
C	1.76034800	-0.60861100	0.00001200
N	3.12529300	-0.70803800	0.00001400
C	-0.47078600	-1.45942600	0.00008900
C	-0.85409200	-0.12490800	0.00008700
C	-0.02034500	1.03819200	0.00004200
C	1.39740900	0.73259300	-0.00001100
N	-2.22698600	-0.04129400	0.00010000
C	-2.79836500	1.23941000	0.00011600
C	-1.90360700	2.34918200	0.00006500
N	-0.52987700	2.24142600	0.00002000
C	-4.17400500	1.46730800	0.00018500
C	-4.67918000	2.75524800	0.00018900
C	-3.81497200	3.85158000	0.00012300
C	-2.45236400	3.64367400	0.00006300
C	2.61162300	1.49957500	-0.00013000
C	3.64080900	0.57142600	-0.00000300
C	-1.66748800	-2.20475300	0.00013800
C	-2.76027800	-1.34449500	0.00010900
Si	2.88105000	3.36428600	-0.00030500
C	2.13264000	4.12472500	-1.54688000
C	2.13250400	4.12501000	1.54606600
C	4.74335600	3.66793700	-0.00025500
Si	-4.52292000	-2.02646400	0.00004200
C	-5.47021700	-1.55168300	-1.55953900
C	-4.36158100	-3.90137300	-0.00004300
C	-5.47029500	-1.55185100	1.55962900
H	6.69204700	-3.57926700	0.00011000
H	5.18167600	-5.54824600	0.00016800
H	2.71954500	-5.21285900	0.00015600
H	5.75760700	-1.30334900	0.00004100
H	-4.85867900	0.63788900	0.00024600
H	-5.75104500	2.90492500	0.00024500
H	-4.21193600	4.85832700	0.00012200
H	-1.75650000	4.47194900	0.00001900
H	4.70384400	0.73475300	0.00000500
H	-1.72956100	-3.27932500	0.00015900
H	2.28647400	5.20715700	-1.55950400
H	2.58733400	3.71235200	-2.45060600
H	1.05986300	3.93169200	-1.58586300
H	2.28636100	5.20743900	1.55851600
H	2.58709700	3.71278300	2.44990900
H	1.05971800	3.93201100	1.58497700
H	5.22694100	3.24646100	-0.88482300
H	4.94621000	4.74201700	-0.00036400
H	5.22686600	3.24665400	0.88444500
H	-6.45781800	-2.02107300	-1.54685700
H	-5.61628600	-0.47971100	-1.69190500
H	-4.94114600	-1.92041800	-2.44156200
H	-5.36127000	-4.34383300	-0.00007500
H	-3.83988300	-4.27210800	0.88415500
H	-3.83986100	-4.27202600	-0.88426300
H	-4.94130100	-1.92074800	2.44163100
H	-5.61631200	-0.47989100	1.69215000

H	-6.45792100	-2.02118400	1.54681400

3*:

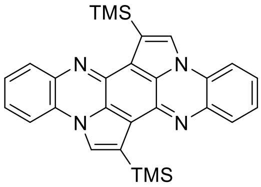

C	-3.66274500	-4.75879000	-0.00002800
C	-4.66308400	-3.78238700	-0.00002500
C	-4.32509600	-2.44428000	-0.00001900
C	-2.98217400	-2.02947600	-0.00001500
C	-1.98235800	-3.03975300	-0.00001700
C	-2.32915700	-4.39028200	-0.00002400
N	-2.71196800	-0.67535300	-0.00001100
C	-1.45719100	-0.30225000	-0.00001000
C	-0.39345600	-1.25851700	-0.00001000
N	-0.65766400	-2.60245200	-0.00001400
C	-0.98393300	1.06681800	-0.00000700
C	0.39345600	1.25851700	-0.00001100
C	1.45719100	0.30225000	-0.00001200
C	0.98393300	-1.06681800	-0.00000800
N	0.65766300	2.60245200	-0.00001400
C	1.98235800	3.03975300	-0.00001900
C	2.98217400	2.02947700	-0.00001800
N	2.71196800	0.67535300	-0.00001400
C	2.32915700	4.39028200	-0.00002600
C	3.66274500	4.75879000	-0.00003100
C	4.66308400	3.78238700	-0.00002900
C	4.32509600	2.44428000	-0.00002300
C	1.59608000	-2.36544200	0.00000400
C	0.54864100	-3.27160900	-0.00001800
C	-1.59608000	2.36544200	0.00000400
C	-0.54864100	3.27160900	-0.00001700
Si	3.41459400	-2.85849300	0.00002800
C	4.25863700	-2.20475400	-1.54590000
C	4.25858100	-2.20479000	1.54600200
C	3.49071500	-4.74363000	0.00000300
Si	-3.41459400	2.85849300	0.00003000
C	-4.25857900	2.20479000	1.54600400
C	-3.49071500	4.74363000	0.00000500
C	-4.25863900	2.20475500	-1.54589800
H	-3.92699800	-5.80815400	-0.00003300
H	-5.70461400	-4.07648300	-0.00002800
H	-5.08401700	-1.67324600	-0.00001800
H	-1.55720000	-5.14775000	-0.00002700
H	1.55720000	5.14775000	-0.00002800
H	3.92699800	5.80815400	-0.00003600
H	5.70461400	4.07648300	-0.00003300
H	5.08401700	1.67324600	-0.00002300
H	0.57915500	-4.34666600	-0.00002400
H	-0.57915500	4.34666600	-0.00002200
H	5.31471100	-2.48754900	-1.55971500
H	3.79412800	-2.60435400	-2.45036000
H	4.19626000	-1.11635900	-1.58236900
H	5.31464000	-2.48763600	1.55988400

H	3.79399700	-2.60436200	2.45043700
H	4.19625100	-1.11639200	1.58246200
H	3.01372000	-5.17241900	-0.88463700
H	4.53216300	-5.07549200	-0.00001500
H	3.01374100	-5.17244500	0.88464100
H	-5.31463500	2.48764700	1.55989400
H	-4.19626100	1.11639100	1.58245600
H	-3.79398500	2.60435000	2.45043800
H	-4.53216300	5.07549200	-0.00002100
H	-3.01371300	5.17241900	-0.88463100
H	-3.01374800	5.17244500	0.88464700
H	-3.79413700	2.60436200	-2.45035800
H	-4.19625300	1.11636000	-1.58237200
H	-5.31471500	2.48754100	-1.55970700

4*:

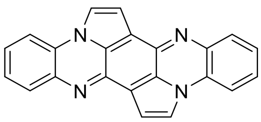

C	5.99418300	-0.39070900	0.00002200
C	5.92071600	1.00530000	0.00002300
C	4.69256000	1.63403900	0.00001300
C	3.49375500	0.89978300	0.00000200
C	3.59364800	-0.51957000	0.00000000
C	4.83737100	-1.14949500	0.00001100
N	2.30041200	1.59342900	-0.00000500
C	1.19578600	0.89396800	-0.00001100
C	1.21006000	-0.53401200	-0.00001500
N	2.39142600	-1.22839000	-0.00001300
C	-0.14827700	1.43337300	-0.00001600
C	-1.21006000	0.53401200	-0.00002000
C	-1.19578600	-0.89396800	-0.00001400
C	0.14827700	-1.43337300	-0.00001500
N	-2.39142600	1.22839000	-0.00001100
C	-3.59364800	0.51957100	0.00000000
C	-3.49375500	-0.89978300	0.00000100
N	-2.30041200	-1.59342900	-0.00000400
C	-4.83737100	1.14949500	0.00000900
C	-5.99418300	0.39070900	0.00001900
C	-5.92071600	-1.00530000	0.00002000
C	-4.69256000	-1.63403900	0.00001200
C	0.72783400	-2.72833100	-0.00000800
C	2.09753900	-2.57768000	-0.00000700
C	-0.72783400	2.72833100	-0.00001100
C	-2.09753900	2.57768000	0.00000200
H	6.95692100	-0.88467100	0.00003200
H	6.82908400	1.59352300	0.00003100
H	4.61032500	2.71276300	0.00001300
H	4.89722100	-2.22936300	0.00001200
H	-4.89722100	2.22936300	0.00000800
H	-6.95692100	0.88467100	0.00002600
H	-6.82908400	-1.59352400	0.00002800
H	-4.61032400	-2.71276300	0.00001300
H	0.20125200	-3.66773800	-0.00000600
H	2.87276300	-3.32215200	-0.00001500
H	-0.20125200	3.66773800	-0.00001300

H	-2.87276300	3.32215200	0.00000800

3*$^{2+}$:

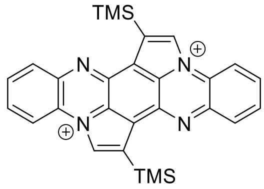

C	-2.46398600	5.41607800	-0.00009300
C	-3.69034000	4.69062300	-0.00009800
C	-3.68541000	3.33167100	-0.00008100
C	-2.44850100	2.61168400	-0.00005600
C	-1.19927700	3.38943100	-0.00005100
C	-1.24176700	4.78750900	-0.00007100
N	-2.48729000	1.28815500	-0.00004200
C	-1.32410700	0.60464000	-0.00002100
C	-0.09595400	1.30448000	-0.00001500
N	-0.05140900	2.66782200	-0.00003100
C	-1.21491900	-0.80940300	-0.00000700
C	0.09595400	-1.30448000	0.00001400
C	1.32410700	-0.60464000	0.00002000
C	1.21491900	0.80940300	0.00000600
N	0.05140900	-2.66782200	0.00003000
C	1.19927700	-3.38943100	0.00005000
C	2.44850100	-2.61168400	0.00005500
N	2.48729000	-1.28815500	0.00004100
C	1.24176700	-4.78750900	0.00006900
C	2.46398600	-5.41607800	0.00009100
C	3.69034000	-4.69062300	0.00009500
C	3.68541000	-3.33167100	0.00007800
C	2.12502200	1.95438100	0.00000500
C	1.31710100	3.05168500	-0.00002000
C	-2.12502200	-1.95438100	-0.00000500
C	-1.31710100	-3.05168500	0.00001900
Si	4.04855800	2.06000100	0.00000100
C	4.66078100	1.22451500	1.55893200
C	4.46527300	3.88428900	0.00001900
C	4.66072300	1.22453400	-1.55896600
Si	-4.04855800	-2.06000100	0.00000100
C	-4.66078500	-1.22451300	-1.55892800
C	-4.66072000	-1.22453600	1.55897100
C	-4.46527300	-3.88428900	-0.00001800
H	-2.49787900	6.49825300	-0.00010800
H	-4.62395400	5.23657000	-0.00011600
H	-4.59756200	2.75139100	-0.00008500
H	-0.33053700	5.36781500	-0.00006800
H	0.33053700	-5.36781500	0.00006700
H	2.49787900	-6.49825300	0.00010600
H	4.62395400	-5.23657000	0.00011300
H	4.59756200	-2.75139100	0.00008300
H	1.57813700	4.09516100	-0.00003600
H	-1.57813700	-4.09516100	0.00003500
H	4.38359400	0.17032900	1.59553400
H	5.75143500	1.28251200	1.59948400
H	4.27603300	1.71222800	2.45682400

H	4.09249400	4.40042800	-0.88764900
H	5.55156000	4.00343100	0.00020600
H	4.09219100	4.40047500	0.88753200
H	4.38388200	0.17025100	-1.59536500
H	4.27561500	1.71199700	-2.45683900
H	5.75134900	1.28289200	-1.59976600
H	-4.27605500	-1.71223800	-2.45682100
H	-5.75144000	-1.28249100	-1.59946700
H	-4.38357900	-0.17033300	-1.59553900
H	-4.27559100	-1.71198600	2.45684200
H	-5.75134400	-1.28291400	1.59978400
H	-4.38389800	-0.17024700	1.59535900
H	-5.55156000	-4.00343100	-0.00021900
H	-4.09218000	-4.40047800	-0.88752500
H	-4.09250500	-4.40042500	0.88765600

6 References

[1] G. R. Fulmer, A. J. M. Miller, N. H. Sherden, H. E. Gottlieb, A. Nudelman, B. M. Stoltz, J. E. Bercaw, K. I. Goldberg, *Organometallics* **2010**, *29*, 2176–2179.
[2] S. Miao, A. L. Appleton, N. Berger, S. Barlow, S. R. Marder, K. I. Hardcastle, U. H. F. Bunz, *Chem. Eur. J.* **2009**, *15*, 4990–4993.
[3] J. U. Engelhart, B. D. Lindner, O. Tverskoy, F. Rominger, U. H. F. Bunz, *Chem. Eur. J.* **2013**, *19*, 15089–15092.
[4] a) O. Tverskoy, F. Rominger, A. Peters, H. J. Himmel, U. H. F. Bunz, *Angew. Chem.* **2011**,*123*, 3619–3622; *Angew. Chem., Int. Ed.* **2011**, *50*, 3557–3560.
[5] J. U. Engelhart, B. D. Lindner, O. Tverskoy, F. Rominger, U. H. F. Bunz, *Chem. Eur. J.* **2013**, *19*, 15089–15092.
[6] E. Epifanovsky, A. T. B. Gilbert, X. Feng, J. Lee, Y. Mao, N. Mardirossian, P. Pokhilko, A. F. White, M. P. Coons, A. L. Dempwolff, Z. Gan, D. Hait, P. R. Horn, L. D. Jacobson, I. Kaliman, J. Kussmann, A. W. Lange, K. U. Lao, D. S. Levine, J. Liu, S. C. McKenzie, A. F. Morrison, K. D. Nanda, F. Plasser, D. R. Rehn, M. L. Vidal, Z.-Q. You, Y. Zhu, B. Alam, B. J. Albrecht, A. Aldossary, E. Alguire, J. H. Andersen, V. Athavale, D. Barton, K. Begam, A. Behn, N. Bellonzi, Y. A. Bernard, E. J. Berquist, H. G. A. Burton, A. Carreras, K. Carter-Fenk, R. Chakraborty, A. D. Chien, K. D. Closser, V. Cofer-Shabica, S. Dasgupta, M. de Wergifosse, J. Deng, M. Diedenhofen, H. Do, S. Ehlert, P.-T. Fang, S. Fatehi, Q. Feng, T. Friedhoff, J. Gayvert, Q. Ge, G. Gidofalvi, M. Goldey, J. Gomes, C. E. González-Espinoza, S. Gulania, A. O. Gunina, M. W. D. Hanson-Heine, P. H. P. Harbach, A. Hauser, M. F. Herbst, M. Hernández Vera, M. Hodecker, Z. C. Holden, S. Houck, X. Huang, K. Hui, B. C. Huynh, M. Ivanov, Á. Jász, H. Ji, H. Jiang, B. Kaduk, S. Kähler, K. Khistyaev, J. Kim, G. Kis, P. Klunzinger, Z. Koczor-Benda, J. H. Koh, D. Kosenkov, L. Koulias, T. Kowalczyk, C. M. Krauter, K. Kue, A. Kunitsa, T. Kus, I. Ladjánszki, A. Landau, K. V. Lawler, D. Lefrancois, S. Lehtola, R. R. Li, Y.-P. Li, J. Liang, M. Liebenthal, H.-H. Lin, Y.-S. Lin, F. Liu, K.-Y. Liu, M. Loipersberger, A. Luenser, A. Manjanath, P. Manohar, E. Mansoor, S. F. Manzer, S.-P. Mao, A. V. Marenich, T. Markovich, S. Mason, S. A. Maurer, P. F. McLaughlin, M. F. S. J. Menger, J.-M. Mewes, S. A. Mewes, P. Morgante, J. W. Mullinax, K. J. Oosterbaan, G. Paran, A. C. Paul, S. K. Paul, F. Pavošević, Z. Pei, S. Prager, E. I. Proynov, Á. Rák, E. Ramos-Cordoba, B. Rana, A. E. Rask, A. Rettig, R. M. Richard, F. Rob, E. Rossomme, T. Scheele, M. Scheurer, M. Schneider, N. Sergueev, S. M. Sharada, W. Skomorowski, D. W. Small, C. J. Stein, Y.-C. Su, E. J. Sundstrom, Z. Tao, J. Thirman, G. J. Tornai, T. Tsuchimochi, N. M. Tubman, S. P. Veccham, O. Vydrov, J. Wenzel, J. Witte, A. Yamada, K. Yao, S. Yeganeh, S. R. Yost, A. Zech, I. Y. Zhang, X. Zhang, Y. Zhang, D. Zuev, A. Aspuru-Guzik, A. T. Bell, N. A. Besley, K. B. Bravaya, B. R. Brooks, D. Casanova, J.-D. Chai, S. Coriani, C. J. Cramer, G. Cserey, A. E. DePrince, R. A. DiStasio, A. Dreuw, B. D. Dunietz, T. R. Furlani, W. A. Goddard, S. Hammes-Schiffer, T. Head-Gordon, W. J. Hehre, C.-P. Hsu, T.-C. Jagau, Y. Jung, A. Klamt, J. Kong, D. S. Lambrecht, W. Liang, N. J. Mayhall, C. W. McCurdy, J. B. Neaton, C. Ochsenfeld, J. A. Parkhill, R. Peverati, V. A. Rassolov, Y. Shao, L. V. Slipchenko, T. Stauch, R. P. Steele, J. E. Subotnik, A. J. W. Thom, A. Tkatchenko, D. G. Truhlar, T. van Voorhis, T. A. Wesolowski, K. B. Whaley, H. L. Woodcock, P. M. Zimmerman, S. Faraji, P. M. W. Gill, M. Head-Gordon, J. M. Herbert, A. I. Krylov, *J. Chem. Phys.* **2021**, *155*, 84801.
[7] F. Neese, *WIREs Comput. Mol. Sci.* **2012**, *2*, 73–78.
[8] *Gaussian 16, Revision B.01,* M. J. Frisch, G. W. Trucks, H. B. Schlegel, G. E. Scuseria, M. A. Robb, J. R. Cheeseman, G. Scalmani, V. Barone, G. A. Petersson, H. Nakatsuji, X. Li, M. Caricato, A. V. Marenich, J. Bloino, B. G. Janesko, R. Gomperts, B. Mennucci, H. P. Hratchian, J. V. Ortiz, A. F. Izmaylov, J. L. Sonnenberg, D. Williams-Young, F. Ding, F. Lipparini, F. Egidi, J. Goings, B. Peng, A. Petrone, T. Henderson, D. Ranasinghe, V. G. Zakrzewski, J. Gao, N. Rega, G. Zheng, W. Liang, M. Hada, M. Ehara, K. Toyota, R. Fukuda, J. Hasegawa, M. Ishida, T. Nakajima, Y. Honda, O. Kitao, H. Nakai, T. Vreven, K. Throssell, J. A. Montgomery, Jr., J. E. Peralta, F. Ogliaro, M. J. Bearpark, J. J. Heyd, E. N. Brothers, K. N. Kudin, V. N. Staroverov, T. A. Keith, R. Kobayashi, J. Normand, K.

Raghavachari, A. P. Rendell, J. C. Burant, S. S. Iyengar, J. Tomasi, M. Cossi, J. M. Millam, M. Klene, C. Adamo, R. Cammi, J. W. Ochterski, R. L. Martin, K. Morokuma, O. Farkas, J. B. Foresman, and D. J. Fox, Gaussian, Inc., Wallingford CT, **2016**.
